# Subtype heterogeneity and epigenetic convergence in neuroendocrine prostate cancer

**DOI:** 10.1038/s41467-021-26042-z

**Published:** 2021-10-01

**Authors:** Paloma Cejas, Yingtian Xie, Alba Font-Tello, Klothilda Lim, Sudeepa Syamala, Xintao Qiu, Alok K. Tewari, Neel Shah, Holly M. Nguyen, Radhika A. Patel, Lisha Brown, Ilsa Coleman, Wenzel M. Hackeng, Lodewijk Brosens, Koen M. A. Dreijerink, Leigh Ellis, Sarah Abou Alaiwi, Ji-Heui Seo, Sylvan Baca, Himisha Beltran, Francesca Khani, Mark Pomerantz, Alessandra Dall’Agnese, Jett Crowdis, Eliezer M. Van Allen, Joaquim Bellmunt, Colm Morrisey, Peter S. Nelson, James DeCaprio, Anna Farago, Nicholas Dyson, Benjamin Drapkin, X. Shirley Liu, Matthew Freedman, Michael C. Haffner, Eva Corey, Myles Brown, Henry W. Long

**Affiliations:** 1 Department of Medical Oncology, Dana-Farber Cancer Institute, Brigham and Women’s Hospital, and Harvard Medical School, Boston, MA USA; 2 Center for Functional Cancer Epigenetics, Dana-Farber Cancer Institute, Boston, MA USA; 3 Translational Oncology Laboratory, Hospital La Paz Institute for Health Research (IdiPAZ) and CIBERONC, La Paz University Hospital, Madrid, Spain; 4 Broad Institute of MIT and Harvard, Cambridge, MA USA; 5 Department of Urology, University of Washington, Seattle, WA USA; 6 Divisions of Human Biology and Clinical Research, Fred Hutchinson Cancer Research Center, Seattle, WA USA; 7 Department of Pathology, University Medical Center Utrecht, Utrecht University, Utrecht, The Netherlands; 8 Department of Endocrinology, Amsterdam UMC, Amsterdam, The Netherlands; 9 Department of Oncologic Pathology, Dana-Farber Cancer Institute and Harvard Medical School, Boston, MA USA; 10 Weill Cornell Medical Center, Department of Pathology and Laboratory Medicine, New York Presbyterian Hospital, New York, NY USA; 11 Whitehead Institute for Biomedical Research, 455 Main Street, Cambridge, MA 02142 USA; 12 Beth Israel Deaconess Medical Center and PSMAR-IMIM Lab. Harvard Medical School, Boston, Massachusetts USA; 13 Massachusetts General Hospital Cancer Center, Boston, MA USA; 14 Nancy B. and Jake L. Hamon Center for Therapeutic Oncology Research, Dallas, TX USA; 15 Harold C. Simmons Comprehensive Cancer Center, University of Texas Southwestern Medical Center, Dallas, TX USA; 16 Department of Internal Medicine, University of Texas Southwestern Medical Center, Dallas, TX USA; 17 Department of Data Science, Dana-Farber Cancer Institute, Harvard T.H. Chan School of Public Health, Boston, MA USA; 18 Division of Clinical Research, Fred Hutchinson Cancer Research Center, Seattle, WA USA; 19 Department of Pathology, University of Washington, Seattle, WA USA

**Keywords:** Cancer genomics, Neuroendocrine cancer, Epigenetics

## Abstract

Neuroendocrine carcinomas (NEC) are tumors expressing markers of neuronal differentiation that can arise at different anatomic sites but have strong histological and clinical similarities. Here we report the chromatin landscapes of a range of human NECs and show convergence to the activation of a common epigenetic program. With a particular focus on treatment emergent neuroendocrine prostate cancer (NEPC), we analyze cell lines, patient-derived xenograft (PDX) models and human clinical samples to show the existence of two distinct NEPC subtypes based on the expression of the neuronal transcription factors ASCL1 and NEUROD1. While in cell lines and PDX models these subtypes are mutually exclusive, single-cell analysis of human clinical samples exhibits a more complex tumor structure with subtypes coexisting as separate sub-populations within the same tumor. These tumor sub-populations differ genetically and epigenetically contributing to intra- and inter-tumoral heterogeneity in human metastases. Overall, our results provide a deeper understanding of the shared clinicopathological characteristics shown by NECs. Furthermore, the intratumoral heterogeneity of human NEPCs suggests the requirement of simultaneous targeting of coexisting tumor populations as a therapeutic strategy.

## Abstract (web summary)

Neuroendocrine carcinomas (NECs) arise from different anatomic sites, but have similar histological and clinical features. Here, the authors show that the epigenetic landscape of a range of NECs converges towards a common epigenetic state, while distinct subtypes occur within neuroendocrine prostate cancer contributing to intratumor heterogeneity in clinical samples.

## Introduction

Neuroendocrine carcinomas (NECs) are high-grade tumors that can arise in the lung, colon, prostate, or bladder among other anatomic sites. NECs are characterized by aggressive clinical behavior and poor prognosis^1^. Histomorphologically, NEC comprises a group of tumors that can have features of small-cell carcinoma and show expression of neuroendocrine (NE) markers including SYP, CHGA, and INSM1^2^. Given these common characteristics, NECs constitute a unique clinicopathological entity despite their distinct anatomical origins^1^. From a genetic standpoint, NECs are often characterized by genomic aberrations in *RB1* and *TP53*^3^. The association of these genetic alterations with NEC etiology is exemplified in Merkel Cell Carcinoma (MCC). MCC is frequently caused by clonal integration of Merkel cell polyomavirus DNA, which causes persistent expression of viral T antigens that interfere with RB1^4^ (MCC Polyomavirus-positive). Gastrointestinal NEC (GINEC) also typically harbor *TP53* and *RB1* alterations, and are clinically aggressive and highly proliferative in contrast to the well-differentiated GI carcinoids that, while also showing a NE phenotype, are typically clinically indolent^1^ and not associated with *TP53* and *RB1* alterations.

NEC can emerge either de novo or as a result of therapeutic pressure^5,6^. Small-cell lung cancer (SCLC) most often occurs de novo but can emerge after treatment of *EG*FR mutant lung adenocarcinoma (AD)^6^. SCLC has been subclassified based on the differential expression of the basic helix-loop-helix (bHLH) transcription factors (TFs) ASCL1 and NEUROD1^7^. These neuronal lineage TFs (LTFs) have been implicated in the maturation of resident NE cells of the lung^8,9^. They are also involved in the carcinogenic process as shown in mouse models of SCLC where ASCL1 is required for tumor formation^10^. NE prostate cancer (NEPC), in contrast, arises most frequently as a treatment-emergent phenotype from prostatic ADs after treatment to repress Androgen Receptor (AR) pathway activity^5^ and only rarely arise de novo. NEPC has poor prognosis, very limited therapeutic options, and is currently treated as a homogeneous disease.

Here we show the chromatin profiles of a range of NECs and identify convergence to a common epigenetic state. We show the existence of subtypes in treatment-emergent NEPC concordant with what has been described in de novo SCLC. These subtypes co-exist as separate subpopulations with distinct chromatin states within the same human NEPC specimens. The observed intra-tumoral heterogeneity of clinical NEPC samples has therapeutic implications.

## Results

### NECs share a common landscape of DNA-accessible regions

**Fig. 1:**
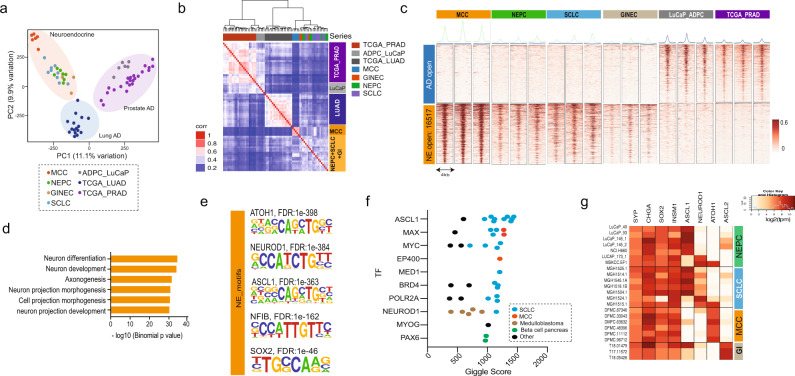
NE carcinomas share a common chromatin state independent of their anatomical origin. **a** Principal component analysis (PCA) of ATAC-seq data of NECs including Merkel cell carcinoma (MCC), neuroendocrine prostate cancer (NEPC), gastrointestinal neuroendocrine carcinoma (GINE), and small-cell lung cancer (SCLC). The plot also includes prostate adenocarcinoma (PDX models and TCGA primary tissues) and lung adenocarcinoma (TCGA primary tissues). **b** Hierarchical clustering of the pairwise Pearson’s correlation of the ATAC-seq signal across the distinct tumor types. **c** Heatmap representation of the differential regions between representative ADs and NECs. Each row is a peak location and each column is a sample. Shown above each column are the composite tag density plots for the AD sites (blue) and NE sites (green). **d** Gene Ontology enrichment using a binomial test^17^ showing the pathways enriched in genes with nearby NE-specific accessible regions shown in **c**. **e** Top results from motif analysis of the NE-specific accessible regions. **f** Public ChIP-seq data sets showing the highest overlap with the NE-specific accessible regions as determined by CistromeDB toolkit annotated by tissue type. The TFs are ordered by the top scoring data set of each type. **g** Expression of NE markers and bHLH TFs across all the NEC samples in our study displayed as a heatmap. Source data are provided as a Source Data file.

Histomorphologically, NECs show similarities that could result from activation of common transcriptional regulators^11^. To investigate the impact of chromatin accessibility in determining the NEC phenotype, we profiled the epigenetic landscape of NECs arising in various anatomic locations using assay for transposase-accessible chromatin with high-throughput sequencing (ATAC-seq) and RNA sequencing (RNA-seq) applied to patient-derived xenograft (PDX) models of NEPC^12^, SCLC^13^, and MCC, as well as GINEC clinical samples (Supplementary Table 1). As NEC can emerge from a preexisting AD, as typified by NEPC^5^ and occasionally by SCLC^6^, we hypothesized that those histologies are extremes of a spectrum of tumor progression. To determine how the chromatin state differs between NE and AD by ATAC-seq analysis, we also generated data from metastatic prostate AD (PRAD) PDX models and used The Cancer Genome Atlas data for primary PRADs and non-small-cell lung ADs^14^. We obtained high-quality data with the fraction of reads in peaks (FrIP) scores in the range of 10–35 and peak numbers in the range of 25–75k (Supplementary Table 1). Replicate profiling of samples showed high concordance (Supplementary Fig. 1a). Unsupervised principal component analysis (PCA) performed on the ATAC-seq data revealed that the NECs cluster together, indicating a convergent chromatin state, in contrast to the ADs that are segregated by anatomic site (Fig. 1a). To test this result, we have also analyzed previously published ATAC-seq results from engineered cells that express defined sets of oncogenic drivers to reprogram normal basal prostate cells to an NE state^15^. The close proximity in the PCA plot of the cluster of terminal NEPC engineered cells (“PARCB,” expressing dominant-negative TP53, myrAkt1, RB1-shRNA, c-MYC, and BCL2) to the clinical NECs illustrates the functional impact of those genetic alterations to reprogram the chromatin state from the basal epithelial prostate cells toward the NEPC phenotype (Supplementary Fig. 1b). The sample–sample correlation of the ATAC-seq peaks for our data set also supports the result that NECs are more similar to each other than to their AD counterparts from the same tissue (Fig. 1b). These analyses also clustered prostate PDXs and primary human tumors together emphasizing, in terms of the chromatin state, the value of the prostate LuCaP^16^ PDXs to model human prostate cancer, as previously validated by histological and molecular characterization^16^.

To investigate epigenetic drivers involved in the NE chromatin state, we performed a supervised analysis of the DNA accessibility between ADs and NECs, and found a high number (n:16517, *P*_adj_ < 0.001, log2(FC) > 2) of NE-specific accessible sites shared across all NE tumor types (Fig. 1c and Supplementary Fig. 1c). The NEC-specific chromatin signature is also represented in the engineered prostate cells^15^ (Supplementary Fig. 1d), which show a clear progression towards increasing signal at the NE-specific accessible sites, while showing little to no signal at the AD-specific sites in the heatmap (Supplementary Fig. 1d). We also determined the nearest gene to each site and translated the ATAC signature into an RNA-seq signature and found that we can distinguish castration-resistant prostate cancer (CRPC) from NEPC in patient cohorts (Supplementary Fig. 1e) further validating the signatures from our PDX models. The major differences in chromatin organization between ADs and NECs were further investigated by Genomic Regions Enrichment of Annotations Tool (GREAT) analysis^17^ associating genomic regions with nearby genes and then examining the enrichment of Gene Ontology (GO) pathways. Genes near NE-specific DNA-accessible regions showed a significant enrichment in pathways for neural differentiation, development, morphology, and axogenesis (Fig. 1d). Next, we used HOMER^18^ to investigate NE-specific sites for enrichment of TF DNA-binding motifs. This analysis revealed significant enrichment for motifs of the basic bHLH TF family, specifically for ATOH1, ASCL1, and NEUROD1, as well as motifs for NFIB, SOX2, and NKX2-1 (Fig. 1e and Supplementary Table 2). ATOH1 has been implicated as a LTF in MCC^19,20^, whereas ASCL1 and NEUROD1 have been suggested to have a corresponding role in SCLC^10,21^. NFIB is a TF previously implicated in rewiring the chromatin structure in SCLC^22^, whereas SOX2 and NKX2-1 are also known to be associated with SCLC^23,24^. Examining what motifs co-occur in the ATAC-seq peaks, we observed that the module of the main three motifs (ASCL1, ATOH1, and NEUROD1) occurs very frequently combined with either SOX2 or NFIB, or with both of them simultaneously (Supplementary Fig. 1e).

By comparing the NE-specific sites to published chromatin immunoprecipitation sequencing (ChIP-seq) profiles compiled in CistromeDB^25^, we identified TFs whose published binding sites have the highest overlap with the NE-specific ATAC-seq peaks as quantified by GIGGLE score^26^. Consistent with the observed shared epigenetic program among NECs, the top overlapping ChIP-seq data sets were generated from SCLC, MCC, or neural lineages (Fig. 1f). In particular, binding profiles of ASCL1 (SCLC), NEUROD1 (SCLC and medulloblastoma), and MAX (MCC)^4^ had the highest overlap scores with NE-specific accessible chromatin (Fig. 1f). We next analyzed the expression of these TFs and other NEC-associated factors within our study samples. As expected, we observed a strong commonality in the expression of NE markers (*SYP*, *CHGA*, and *INSM1*) and the stemness TF *SOX2* (Fig. 1g) across NECs. We also observed a more mutually exclusive expression pattern of bHLH TFs including *ASCL1* or *NEUROD1* in both NEPC and SCLC, *ASCL1*, and *ASCL2* expression in GI-NECs and *ATOH1* expression in MCC (Fig. 1g). Overall, our results suggest tumor and organ-specific bHLH TFs maintaining the common NE epigenetic state.

### Treatment emergent NEPC can be subclassified based on the expression of ASCL1 and NEUROD1

**Fig. 2:**
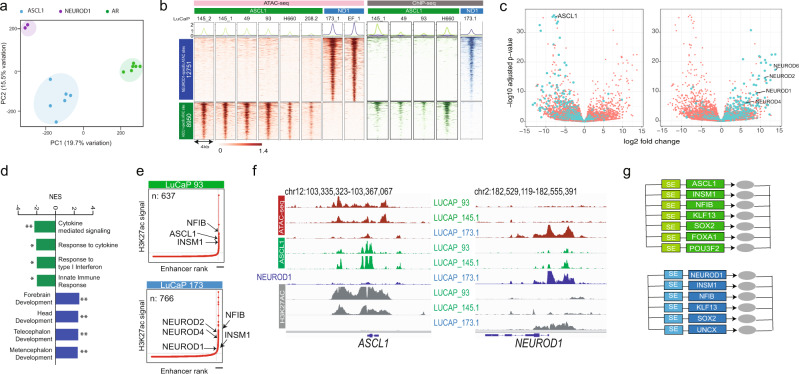
NEPC shows tumor subtypes based on the differential expression of the transcription factors ASCL1 and NEUROD1. **a** Principal component analysis of ATAC-seq data from NEPC and ADPC PDXs. Samples are color coded by the dominant TF expressed in that sample. **b** The left side of the heatmap (red) displays the differential ATAC-seq regions identified between NEPC subtypes. There are 12,751 NEUROD1-specific regions (top) and 8950 ASCL1-specific (bottom) ATAC sites. The right side of the heatmap shows the ChIP-seq data at the same sites for ASCL1 (green) and NEUROD1 (blue) for the indicated samples. **c** Association between differential ATAC-accessible sites and differential gene expression. Each volcano plot depicts RNA-seq log2-fold change (*x*-axis) and *p*-value adjusted for multiple hypothesis testing calculated by DESeq2 using a Wald’s test (*y*-axis). Each dot represents one gene: green indicates a differential ATAC peak is within 50 kB of the gene and orange indicates there is no such peak. Left: ASCL1-specific accessible regions and genes upregulated in ASCL1 subtype; (right) NEUROD1-specific accessible regions and genes upregulated in NEUROD1 subtype. **d** GSEA pathway analysis of genes enriched in the ASCL1 subtype (green) and the NEUROD1 subtype (blue) (**q-value < 0.001, *q-value < 0.05). **e** Signal distribution of H3K27ac marked enhancers from representative cases of the ASCL1 subtype (top) and NEUROD1 subtype (bottom). The bars in the lower right of each plot identify the subset of enhancers known as super-enhancers defined by the ROSE algorithm; 693 were identified in LuCaP 93 (ASCL1) and 766 in LuCaP 173.1 (NEUROD1). Super-enhancers nearby selected genes are indicated by the arrows. **f** Representative IGV tracks at the ASCL1 and NEUROD1 gene loci. ATAC-seq tracks are in red, ASCL1 ChIP-seq in green, NEUROD1 ChIP-seq in blue, and H3K27ac in gray. The loci are marked by subtype-specific super-enhancers with preferential binding of their respective TF. **g** Circuits of lineage transcription factors specific for the ASCL1 subtype (green) and NEUROD1 subtype (blue). Source data are provided as a Source Data file.

To explore heterogeneity in the TF regulation of the NEC epigenetic state, we performed an unsupervised analysis of the ATAC-seq data restricted to the NECs. Regardless of tissue of origin, NECs expressing *ASCL1* and/or *ASCL2* were tightly clustered together and were separated from NECs expressing *ATOH1* or *NEUROD1* (Supplementary Fig. 2a). Furthermore, the similarity in terms of the DNA accessibility shown by SCLC and NEPC depends on the status of *ASCL1* or *NEUROD1* expression but not on the tumor type (Supplementary Fig. 2b). Unsupervised analysis of the DNA accessibility in just prostate samples (Fig. 2a) showed clear grouping associated with the expression of *AR* (all ADPCs), *ASCL1*, or *NEUROD1*, with the same clustering being apparent by analysis of RNA-seq data of those same prostate samples (Supplementary Fig. 2c). Although NE subtypes based on the expression of those TFs have been previously described in SCLC^7,10^, the existence of these subtypes in treatment-emergent NEPC was unanticipated, as ASCL1 and NEUROD1 have been specifically associated with lung NE cells^8,9,27^, the putative cell of origin of the de novo SCLC.

Next, we aimed to identify the differential DNA accessibility associated with the ASCL1 and the NEUROD1 NEPC subtypes. Supervised analysis comparing *ASCL1* and *NEUROD1* expressing NEPC samples identified 8950 ASCL1- and 12,751 NEUROD1-specific accessible regions (false discovery rate (FDR) < 0.01, log2(FC) > 1) (Fig. 2b). We next interrogated the NEPC subtype-specific sites in SCLC and observed similar patterns of chromatin accessibility at these TF-specific genomic regions that, in addition, displayed an association between the chromatin state and the differential expression of *ASCL1* and *NEUROD1* (Supplementary Fig. 2d). Notably, SCLC cases that coexpress *ASCL1* and *NEUROD1* showed combined accessibility at the two sets of regions (Supplementary Fig. 2d). This result underlines the striking similarity in the chromatin state of the tumor subtypes, both in SCLC and in NEPC. It is important to note that despite the clear differences in accessibility associated with the subtypes, still a large number of open chromatin sites (36,493) are shared between these two subtypes as expected, given the NE characteristics in common for both subtypes (Supplementary Fig. 2e).

To further characterize the chromatin differences between the subtypes, we investigated the relationship between TF subtype-specific chromatin accessibility and the ASCL1 and NEUROD1 genomic binding. To that aim, we performed ChIP-seq analysis for each of the two TFs in NEPC models that expressed *ASCL1* or *NEUROD1* (Supplementary Table 3). This analysis identified thousands of highly conserved binding sites with both overlapping and differential sites for each of the two TFs (Supplementary Fig. 2e, f). Importantly, although the vast majority of the 36,493 shared regions show overlapping binding sites for both TFs (Supplementary Fig. 2e), the differential chromatin-accessible sites were bound by the corresponding TF, but not by the other (Fig. 2b). This result is consistent with a role for ASCL1 and NEUROD1 in maintaining the chromatin state in their respective subtypes. We next performed de novo motif analysis at the specific ASCL1- or NEUROD1-binding sites that yielded the expected consensus motifs for ASCL1 and NEUROD1, respectively (Supplementary Fig. 2g), validating the underlying differences between both the TFs. To further investigate the differences between the two TFs and identify potential co-operating factors, we compared the motifs enriched at the shared and the differential ASCL1- and NEUROD1-binding sites (Supplementary Data 1). The results for the shared binding sites showed enrichment for essentially the same motifs identified in the motif analysis of the ATAC-seq sites shared for all NECs in Fig. 1e (Supplementary Table 2). The highest enrichment was for a broad range of bHLH motifs including NEUROD1, ATOH1, and ASCL1, as well as the NFIB motif supporting that they constitute the basic transcriptional module that maintains the chromatin state. The results of the ASCL1-specific binding sites revealed a strong enrichment for NKX2 motifs. As *NKX2-1* is around 16-fold more highly expressed in the ASCL1 subtype and has been previously reported as specific for the ASCL1 subtype in SCLC^10^, it is likely responsible for this enrichment. For the NEUROD1 sites, we found enrichment for EBF and LHX motifs, which could correspond to the neurogenic TFs EBF3 and LHX8 that show higher expression in the NEUROD1 subtype. Supporting the shift in the transcriptional programs activated in NEPC as compared to CRPC, all these TFs showing enriched motifs at either ASCL1 or NEUROD1 are exclusively expressed in NEPC compared with CRPC. Finally, we noted that the NFIB motif is enriched at the differential sites of both TFs and the shared ones, suggesting that this factor is recruited to the bHLH-binding sites regardless of the specific TF being expressed.

We next sought to confirm that the enhancers specific to the ASCL1 and NEUROD1 subtypes are associated with expression of nearby genes by using the expression data generated from the same samples (Fig. 2c). As expected, differential expression analysis showed that *ASCL1* was one of the most upregulated genes in the ASCL1 set, whereas, conversely, the NEUROD1 set showed upregulation of several NEUROD family members including *NEUROD1*/*2*/*4*/*6* (Fig. 2c). Consistent with these differentially accessible regions being functional, we observed a substantial association between differential DNA accessibility and differential gene expression (Fig. 2c). Gene set enrichment analysis to identify pathways differentially over-represented in each of the two subtypes showed that ASCL1-associated gene expression was enriched in GO pathways of response to cytokines^28^, whereas the NEUROD1-associated expression was enriched in brain development pathways (Fig. 2d). Specifically enriched in the ASCL1 subtype are carcinoembryonic antigen-related cell adhesion molecules (*CEACAM*1,5,6,7). Interestingly, CEACAM5 has been investigated to target NEPC using an anti-CEACAM5-SN38 antibody–drug conjugate^29^. In addition, the ASCL1 subtype shows a relatively higher expression of major histocompatibility complex I-related genes (human leukocyte antigen genes, *NLRC5*) as compared to the NEUROD1 subtype, which could be contributing to the enrichment in immune pathways associated with ASCL1. However, the expression is still very low relative to the expression in CRPC. Therefore, similar to other NECs, both subtypes of NEPC show a relatively low expression of antigen presentation pathways.

The binding of ASCL1 and NEUROD1 TFs to their own promoters and nearby enhancers suggests they are working as LTFs in NEPC. LTFs are known to auto-activate their own expression by binding to super-enhancers (SEs) establishing a positive feedback loop. In addition, LTFs form circuits of core TFs driven by the activation of SEs promoting the transcriptional program required to maintain the lineage^30^. Both ASCL1 and NEUROD1 are known to be lineage transcriptional factors in neuronal systems^31,32^. To investigate a potential LTF behavior of both TFs in NEPC, we performed SE analysis by H3K27ac profiling of ASCL1 and NEUROD1 NEPCs, and found that all models showed SE activation in common at a number of TFs (*INSM1* and *NFIB*) regardless of the tumor subtype (Fig. 2e, Supplementary Table 3, and Supplementary Data 2). In addition, we found differential SEs at either *ASCL1* or *NEUROD1* (and other family members) in accordance with their expression status. Based on those characteristics, both ASCL1 and NEUROD1 can be considered as LTFs in NEPC with binding to SEs and activation of their own expression (Fig. 2c, f). We next identified the core circuit of TFs associated with each of the two subtypes applying a previously described method to identify interconnected auto-regulated loops^30^. We identified distinct but highly overlapping sets of TF circuits in these two subtypes (Fig. 2g).

Taken together, our results provide clear evidence of the existence of two molecular subtypes in NEPC model systems. These subtypes share NE phenotypic characteristics but differ in the expression of *ASCL1* and *NEUROD1*, which is associated with distinct chromatin landscapes and gene expression profiles.

### Analysis of tumor heterogeneity in NEPC liver metastases

**Fig. 3:**
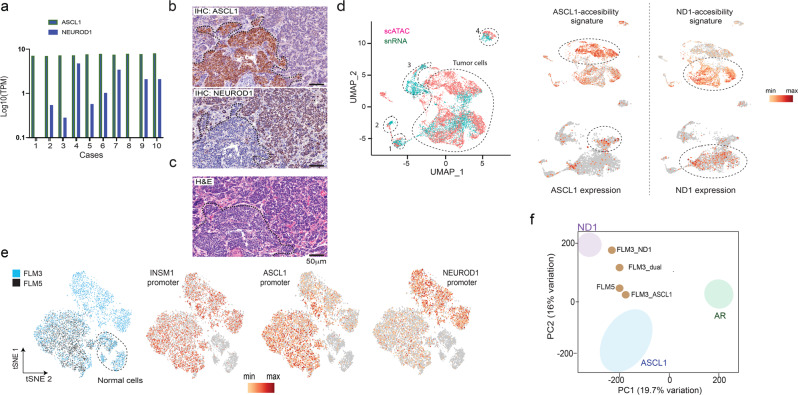
Single-cell analysis reveals that NEPC subtypes co-exist in human metastasis and contribute to inter- and intra-tumoral heterogeneity. **a** Plot of ASCL1 and NEUROD1 expression in NEPC tissues from a clinical cohort^16^. TPM: transcripts per million. **b** Representative immunostaining of FLM3 (ASCL1 staining in the top panel and NEUROD1 staining in the middle panel) showing intratumor heterogeneity. **c** Hematoxylin and eosin staining of the same field illustrates the distinct histologies for the two subpopulations. **d** Combined analysis of the scATAC-seq and snRNA-seq in FLM3 (left). Markers specific for normal cell populations enabled assignment of clusters: 1, vascular cells; 2, stromal cells; 3, hepatic cells; 4, monocytes. Accessibility at the top 30 differential ATAC-seq regions between ASCL1 and NEUROD1 subtypes identified by bulk analysis (top right). Analysis of *ASCL1* and *NEUROD1* expression in the snRNA-seq analysis (bottom right). This analysis matches cells with TF expression and the corresponding differential DNA accessibility for each subtype. **e** tSNE analysis of the combined FLM3 (blue) and FLM5 (black) scATAC-seq data (left). The other three plots show accessibility at *INSM1* promoter (NE marker) and the differential accessibility at *ASCL1* promoter and *NEUROD1* promoter. **f** Projection of the aggregated scATAC-seq clusters for FLM3 and 5 (light brown dots) within the PCA space defined in Fig. 2a. Source data are provided as a Source Data file.

Next, we aimed to determine whether the results from the model systems can be extended to human clinical NEPC. First, we interrogated expression levels of *ASCL1* and *NEUROD1* in tumor tissues from two cohorts of NEPC metastases^16,33^. In contrast to the mutually exclusive expression of the two TFs that we observed in NEPC PDXs (Fig. 1g), clinical samples showed a range of coexpression. The *ASCL1* expression was higher in the majority of the metastases accompanied by a lower and more variable expression of *NEUROD1* for almost all the cases (Fig. 3a and Supplementary Fig. 3a). The differential expression of the TFs in the clinical NEPC samples was associated with enrichment in the corresponding gene signatures identified in our analysis comparing the ASCL1 and NEUROD1 NEPC models (Supplementary Fig. 3b).

To investigate whether these TFs are co-expressed in the same tumor cells or in distinct tumor sub-populations, we studied five distinct fragments of liver metastasis (FLMs) obtained at autopsy from a patient diagnosed with NEPC available as both Optimal cutting temperature compound (OCT compound) frozen and formalin-fixed paraffin-embedded material, and performed RNA-seq to assess expression levels of *ASCL1* and *NEUROD1*. These levels showed a range of coexpression of the two TFs with FLM3 showing the highest relative expression of *NEUROD1* to *ASCL1* (Supplementary Fig. 3c). We next performed immunohistochemical (IHC) analysis for ASCL1 and NEUROD1 protein expression on FLM3. The ASCL1 and NEUROD1 staining showed intra-tumoral heterogeneity that defined two separated tumor populations, which are present at different foci across the tumor section (Fig. 3b and Supplementary Fig. 3d). Both subtypes show an NE phenotype as characterized by the expression of the NE marker INSM1 and absence of AR expression (Supplementary Fig. 3e). Correlation with histomorphological features showed that the two distinct cell populations also differ in their histological characteristics. ASCL1-positive cells had a sheet-like growth pattern and spindle cell morphology, whereas NEUROD1-positive cells appeared to grow in smaller cell clusters with pronounced nuclear molding and focal pleomorphic giant cells (Fig. 3c). We next performed double staining of ASCL1 and NEUROD1 by immunofluorescence (IF) in FLM3, to investigate potential coexpression in tumor cells, and observed that the vast majority of the cells showed an anticorrelated expression of the two TFs (Supplementary Fig. 3f). We extended the IF analysis to six additional NEPC samples and also observed the existence of the same type of intratumor heterogeneity with no ASCL1 and NEUROD1 coexpression (Supplementary Fig. 3g).

We next investigated the two observed intra-tumoral populations by single-cell chromatin (scATAC-seq) and expression (single nucleus RNA sequencing (snRNA-seq)) analysis. We selected FLM3 that showed the highest NEUROD1 expression and FLM5 that had the lowest, almost 200-fold lower than *ASCL1* (Supplementary Fig. 3c). We isolated nuclei from frozen sections of FLM3 and performed scATAC-seq and snRNA-seq to assign the *ASCL1* and *NEUROD1* expression with the corresponding chromatin state. The unsupervised t-distributed stochastic neighbor embedding (tSNE) clustering of the scATAC-seq resulted in multiple clusters that we analyzed for differential accessibility at *SOX2* promoter to distinguish tumor and normal cells. Based on accessibility to *SOX2*, the fraction of the tumor cells represented around 80%. In accordance with their NE phenotype, the tumor cells showed accessibility at the promoter for the NE marker *INSM1* (Supplementary Fig. 3h). Notably, we could distinguish the clusters that correspond to the two tumor subtypes based on the differential accessibility to the *ASCL1* and *NEUROD1* promoters (Supplementary Fig. 3h). The ASCL1 and NEUROD1 clusters also show differential accessibility at the top ATAC differential regions identified by bulk analysis (Supplementary Fig. 3i). An additional cluster was composed of cells that displayed accessibility at either the *ASCL1* or the *NEUROD1* promoter but were intermixed; we labeled that cluster as “mixed” (Supplementary Fig. 3h). We next analyzed the snRNA-seq to identify the tumor cells that express *ASCL1* and *NEUROD1*, and then integrated this data set with the scATAC-seq using SEURAT^34^ (Fig. 3d). This integration enabled the assignment of normal cells based on the expression of specific markers. Crucially, we observed that cells with either the ASCL1 or NEUROD1 accessibility signature developed from bulk data preferentially express the corresponding TF (Fig. 3d). Thus, our results show that ASCL1 and NEUROD1 subtypes exist as separate subpopulations possessing similar epigenetic features as in their respective model systems.

We next investigated the FLM5 sample, which has the highest expression of *ASCL1* by scATAC-seq analysis. In accordance with the RNA-seq, the tSNE analysis showed a single cluster of the FLM5 tumor cells with accessibility at *ASCL1* promoter but not at *NEUROD1* (Supplementary Fig. 3j). The integrated scATAC analysis of FLM3 and FLM5 revealed that 99% of the FLM5 tumor cells overlap the FLM3 ASCL1 cluster (Fig. 3e), indicating that those cells have identical chromatin accessibility. We next plotted the aggregated scATAC-seq by TF cluster from FLMs in the PCA space defined by the model systems in Fig. 2a, which further validated the chromatin state of the two subtypes in the primary tissue (Fig. 3f).

All together, these results demonstrate subtype heterogeneity in human NEPC metastases and that these subtypes show distinct epigenetic characteristics similar to those observed in model systems.

### In the patient metastasis, the NEPC subtypes are distinct but still related clones

**Fig. 4:**
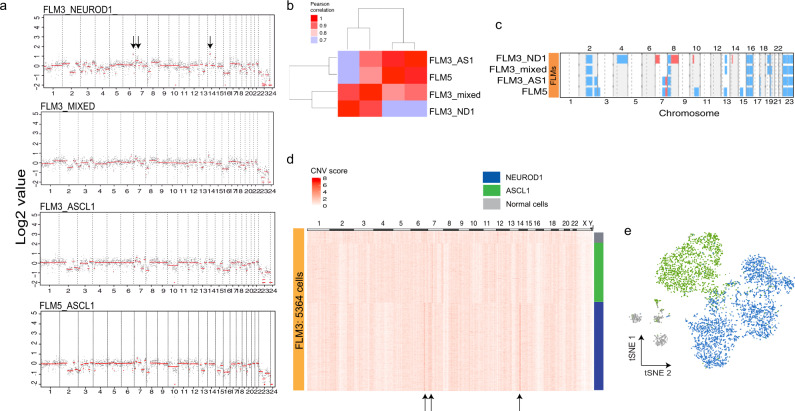
The NEPC subtypes are distinct clones. **a** Genome-wide CNV profiles inferred from the scATAC-seq clusters in FLM3 and FLM5. Black dots are values in 1 MB regions and the red line is the result of running a segmentation algorithm on the data (see “Methods”). Arrows point to differences seen in CNVs across the clusters. **b** Sample pairwise Pearson’s correlation of the CNV profiles. **c** Summary heatmap of the scATAC-seq-inferred CNV alterations across all of the patient samples (blue represents losses and red represents gains). **d** Heatmap of the single-cell CNV analysis of FLM3 where each column is a 2 MB bin tiled across the genome and the rows are individual cells that have been clustered with *K*-means. Arrows point to CNV differences observed here and in the cluster level analysis. **e** tSNE plot of FLM3 scATAC-seq data colored by the cluster each cell was partitioned into by the inferred CNV alterations. Those three clusters clearly correspond to NEUROD1 (blue), ASCL1 (green), and normal cells (gray). Source data are provided as a Source Data file.

We next sought to investigate the genetic characteristics of the NEC samples using whole-exome sequencing (WES) and copy number variation (CNV) inferred from the ATAC-seq data^35^. Inference of *RB1* genetic status from the bulk ATAC-seq data showed a biallelic loss in all the NEPC PDX models but not in the adenocarcimona prostate cancer (ADPC) models (Supplementary Fig. 4a) as previously reported^12^. The same approach was applied genome-wide to the scATAC-seq clusters identified by the tSNE analysis on FLM3 and FLM5. The results show an overall similarity in the CNVs across these clusters (Fig. 4a). For instance, we observed heterozygous losses in all of chr16 and parts of chr2 and chr13 in both ASCL1 and NEUROD1 clusters. In addition, we found a focal heterozygous loss at *PTEN* on chr10 in both clusters. However, clear CNV differences existed, including a 20 MB amplification on chr14p and a chr7p amplification that are only present in the NEUROD1 cluster. Notably, CNVs of the ASCL1 component in FLM3 showed almost identical characteristics with the ASCL1 cluster in FLM5 (Pearson’s correlation = 0.97), whereas showing a lower correlation to the NEUROD1 cluster within the same fragment (Pearson’s correlation = 0.81) (Fig. 4b). WES analysis of the FLM samples, although derived from bulk tissue, validated the scATAC-seq-inferred CNV alterations including amplifications on chr14p and chr7p (Supplementary Fig. 4b).

Finally, we extended the CNV analysis of FLM3 to the single-cell level^35,36^. *K*-means clustering of the cells based on the CNVs distinguished three clusters: one that corresponds to normal cells, with no alterations, and two additional clusters showing alterations. Another cluster of cells had amplifications in chr7p and chr14p, and was associated with the NEUROD1 type and a third cluster without those alterations that was associated with the ASCL1 type (Fig. 4d). We next marked the identity of the cells from the three clusters defined in the genetic analysis within the scATAC-seq tSNE plot from this sample and showed a strong correspondence to the groupings defined by the epigenetic analysis (Fig. 4e). Importantly, this single-cell analysis reinforces the interpretation of the mixed cluster identified in the scATAC-seq analysis, showing that it corresponds to intermixed ASCL1 and NEUROD1 clones instead of an independent clone (Supplementary Fig. 4c, d). Altogether, our results show the existence of distinct genetic clones associated with each of the two NEPC epigenetic subtypes in this patient, likely derived from a common ancestor given their substantial CNV profile overlap.

## Discussion

Poorly differentiated NECs are a class of high-grade tumors that arise at different anatomical sites and typically express markers of NE differentiation (CHGA, NCAM1, and SYP). Our results build considerably on previous work with RNA-seq and cell lines^11,15^, and provide a molecular rationale for the shared histopathological behavior of these tumors based on a common epigenetic state regardless of anatomic origins or the distinct tumor-initiation mechanisms. This epigenetic convergence is associated with the expression of distinct members of the bHLH family, suggesting that a variety of TFs can maintain the NE state.

The similarity in the chromatin state across NECs is particularly pronounced between NEPC and SCLC, which is surprising given the distinct cells of origin in these neoplasms. We observed that treatment-emergent NEPC shows subtypes based on the expression of *ASCL1* and *NEUROD1* as seen in de novo SCLC. This was unexpected, as those TFs have been previously associated with lung development^8,9,27^. Importantly, we show a fundamental difference between the representation of the subtypes in the PDX models as compared to human clinical samples. In contrast to the mutually exclusive expression of *ASCL1* and *NEUROD1* in model systems, tissues from NEPC clinical cohorts show coexpression of *ASCL1* and *NEUROD1* at varying levels. Single-cell analyses of a set of metastatic samples from the same patient revealed the presence of two distinct tumor populations that co-exist within the metastasis. This observation emphasizes that PDXs, despite being good models of the human disease, still offer limitations to illustrate the complexity observed in primary tissues. In fact, those limitations could have precluded a better characterization of subtype coexistence in SCLC^37^, which has mainly been described as homogeneous subtypes^7^. Our results show the existence of subtypes in clinical samples of NEPC and demonstrate heterogeneity in terms of the chromatin state.

The genetic and epigenetic characteristics of NEPC tumors and the newly revealed intra-tumoral heterogeneity of the subtypes can have direct clinical implications for the design of novel treatment strategies. Currently, the standard treatment based on platinum-containing combinations^38,39^ is applied to all patients and typically shows a short duration response. In this respect, our results showing the convergence to a NEC-specific chromatin state underlines the potential value of chromatin remodelers as promising therapeutic targets. Examples of chromatin remodelers already being targeted include enhancer of zeste-homolog 2 (EZH2)^40^ based on preclinical results reporting an effect of EZH2i to re-sensitize tumors to AR-signaling inhibitors in CRPC^33,41^. Notably, alterations in EZH2 have been implicated in de-repression of the TF SOX2 as a consequence of the functional loss of RB1^42,43^, suggesting EZH2 inhibitors as potential agents for NEPC treatment. Another strategy is the targeting of the bromodomain and extraterminal (BET) family. The activity of BET inhibitors regulating the expression of MYC family genes suggests them as candidates for targeting the specific MYC members associated with NEPC^44^. In particular, BRD4 inhibitors have already entered clinical testing based on preclinical data, suggesting that BRD4 could be involved in the transcriptional reprogramming of CRPC^45,46^. The strong similarity in the chromatin state between NEPC and SCLC, and the existence of similar subtypes provides a rationale to extrapolate the previously identified ASCL1- and NEUROD1-specific vulnerabilities in SCLC. In the ASCL1 subtype, e.g., DLL3 is a target for bi- and tri-specific T-cell engager antibodies^47,48^, which are in early phase trials (NCT03319940 and NCT04471727). AURKA is a target for small-molecule inhibitors such as alisertib, which may be more efficacious in the NEUROD1 subtype^49^. Our results also support the potential of targeting CEACAM proteins in ASCL1 + NEPC^29^. We note the possibility that therapeutic strategies that target one but not the other subtype might rapidly succumb to the outgrowth of the resistant subpopulation. Altogether, this new understanding of subtype heterogeneity based on NEUROD1 and ASCL1 illustrates the epigenetic complexity that exists in clinical tumors and provides a rationale for targeting the inter- and intra-tumoral heterogeneity as a therapeutic strategy in NEPC.

## Methods

### Clinical samples and cell lines

Tissue samples were collected within 8 h of death from patients who died of metastatic CRPC. All patients signed informed consent for a rapid autopsy, under the aegis of the Prostate Cancer Donor Program at the University of Washington. Hematoxylin and eosin-stained slides from each case were reviewed by a pathologist, to confirm the presence of tumor cells. All relevant ethical regulations for work with human participants were followed and informed consent was obtained. The Institutional Review Board of the University of Washington (IRB #2341) approved this study. For the PDX models, all relevant ethical regulations for animal testing and research were followed.

NEPC cell line MSKCC EF1 (contributed by Leigh Ellis laboratory) was maintained in RPMI medium supplemented with 10% fetal bovine serum (FBS). NCI-H660 (ATCC catalog number CRL-5813) cells were maintained in HITES medium supplemented with 5% FBS, 0.005 mg/ml Insulin, 0.01 mg/ml Transferrin, 30 nM Sodium selenite, 10 nM Hydrocortisone, 10 nM β-estradiol and, 4 mM l-glutamine. Our cells are routinely assessed for *Mycoplasma* contamination. In addition, we analyzed all of our sequenced libraries for the presence of mycoplasma DNA and all the samples showed the absence of contamination.

### Nuclei preparation

Fragments of frozen tissues (PDX models) or 50 μm sections (liver metastases) were cut and resuspended in 300 μl of cold 3-detergent-ATAC-Resuspension Buffer (RSB) containing 0.1% NP40, 0.1% Tween-20, and 0.01% Digitonin. Tissues were dounced ten times each with a loose and a tight pestle each until homogenization was complete. The homogenate was then transferred to a 1.5 ml pre-chilled microfuge tube and incubated on ice for 10 min. For cell lines, we started from 150,000 cells, washed with 1× phosphate-buffered saline (PBS) and performed lysis in 50 μl of cold RSB containing 0.1% NP40, 0.1% Tween-20, and 0.01% Digitonin. After lysis, 300 μl of ATAC-RSB containing 0.1% Tween-20 was added and the tubes were inverted to mix. Lysates were filtered through a 40 μm cell strainer and nuclei were centrifuged for 10 min at 1500 relative centrifugal force (RCF) in a pre-chilled (4 °C) fixed-angle centrifuge. Nuclei were resuspended with 300 μl of ATAC-RSB containing 0.1% Tween-20 and counted with a hemocytometer using Trypan blue stain.

### ATAC sequencing

Here, 100,000 nuclei were resuspended in 50 μl of transposition mix (25 μl 2× TD buffer, 2.5 μl transposase (100 nM final), 16.5 μl PBS, 0.5 μl 1% Digitonin, 0.5 μl 10% Tween-20, 5 μl H_2_O)^50^. Transposition reactions were incubated at 37 °C for 30 min on a thermomixer. Transposed DNA was purified using Qiagen columns. Libraries were amplified as described previously^51^. Thirty-five basepair paired-end reads were sequenced on a NextSeq instrument (Illumina).

### ChIP sequencing

Nuclei isolated as previously described were crosslinked with 1% formaldehyde for 10 min for H3K27Ac ChIP-seq. For ASCL1 and NEUROD1, ChIP-seq nuclei were crosslinked in two steps with 2 mM of DSG (Pierce) for 45 min at room temperature, followed by 1 ml of 1% formaldehyde for 10 min. Crosslinked nuclei were then quenched with 0.125 M glycine for 5 min at room temperature and washed with PBS. After fixation, pellets were resuspended in 500 μl of 1% SDS (50 mM Tris-HCl pH 8, 10 mM EDTA) and sonicated for 5 min (H3K27ac) or 10 min (ASCL1 and NEUROD1) using a Covaris E220 instrument (setting: 140 peak incident power, 5% duty factor, and 200 cycles per burst) in 1 ml adaptive focused acoustics (AFA) fiber millitubes. Chromatin was immunoprecipitated with 1 μg of H3K27Ac antibody (Diagenode catalog number C15410196), 10 μg of ASCL1 antibody (Abcam ab74065), or 10 μg of NEUROD1 antibody (Cell Signaling mAb #4373). Five micrograms of chromatin was used for H3K27Ac ChIPs and 40 μg of chromatin was used for ASCL1 or NEUROD1 ChIPs. ChIP-seq libraries were made using Rubicon kit and purified. Seventy-five basepair single-end reads were sequenced on a Nextseq instrument (Illumina).

### Single-nuclei ATAC-seq and RNA-seq

Nuclei were prepared as described previously. For scATAC-seq, nuclei were transposed according to the OMNI-ATAC protocol^50^. Approximately 7000 cells were targeted for each sample and processed according to the 10× Genomics scATAC-seq sample preparation protocol (Chromium Single Cell ATAC Library & Gel Bead Kit, 10× Genomics). For snRNA-seq, nuclei prepared the same way were used directly in the 10× Genomics snRNA-seq protocol (Chromium Single Cell 3′ v2 Reagent Kit, 10× Genomics).

### RNA sequencing

A fragment of frozen tissues (PDX models) or 50 μm sections (liver metastases) were cut and homogenized in 1 ml of AllPrep DNA/RNA Mini Kit (Qiagen) using a plastic pestle (Cole-Palmer #44468-23). DNA and RNA were simultaneously isolated. Five hundred nanograms of RNA was used to prepare libraries using the NEBNext Ultra™ RNA Library Prep Kit for Illumina. RNA quantity and quality were assessed on an Agilent 2100 Bioanalyzer. For all RNA-seq, reads were sequenced on a NextSeq 500 instrument (Illumina).

### Whole-exome sequencing

DNA extraction on frozen human FLMs and adjacent normal tissue was performed using the AllPrep DNA/RNA Mini Kit (Qiagen). WES sequencing was performed by Novogene using their standard protocols. Briefly, 1000 ng of genomic DNA were used as input to generate sequencing libraries using the Agilent SureSelect Human All Exon Kit. Captured libraries were enriched by PCR, purified, quantified using the Agilent Bioanalyzer 2100 system, and subsequently sequenced using the NextSeq 500 instrument (Illumina).

### Immunohistochemical analysis

IHC and IF studies using ASCL1 (clone 24B72D11.1, catalog number 556604, BD Biosciences, San Jose, CA) and NEUROD1 (clone EPR17084, catalog number ab205300, Abcam, Cambridge, MA) specific antibodies were carried out on archival formalin-fixed paraffin-embedded tissues. In brief, 5 μm paraffin sections were de-waxed and rehydrated following standard protocols. Antigen retrieval consisted of steaming for 40 min in Target Retrieval Solution (S1700, Agilent, Santa Clara, CA). Slides were then washed and equilibrated in TBS-Tween buffer (Sigma, St. Louis, MO) for 10 min. Primary antibodies were applied at a dilution of 1:25 at 37 °C for 60 min. For chromogenic studies, immunocomplexes were visualized by applying secondary detection reagents of the UltraVision™ Quanto Detection System (catalog number TL-060-QHD, Thermo Fisher, Waltham, MA) following the manufacturer’s instructions. Sequential dual-IF labeling studies were carried out using Tyramide SuperBoost kits (Thermo Fisher, Waltham, MA). All bright-field slides were imaged using a Ventana DP200 system (Roche Diagnostics, Indianapolis, IN). Fluorescence images were acquired on a Cytation 5 Cell Imager (Biotek, Winooski, VT). All the slides have been evaluated by an expert pathologist and the stainings have been replicated a minimum of three times.

### Computational and statistical analysis

#### Analysis of ATAC-seq and ChIP-seq data

A modified version of the ChiLin pipeline was used for quality control and pre-processing of the data^52,53^. We used Burrows-Wheeler Aligner (BWA Version: 0.7.17-r1188) as a read mapping tool to align to hg19 using default parameters. Unique reads for a position for peak calling were used to reduce false-positive peaks and statistically significant peaks were finally selected by calculating a FDR of reported peaks. ATAC peaks were called using MACS2 (v2.1.2) with a cutoff of FDR < 0.01. H3K27ac, ASCL1, and NEUROD1 peaks were called using MACS2 using the same cutoff. DESeq2 was used within the COBRA pipeline^54^ to identify differential peaks in ATAC-seq and ChIP-seq, where gained or lost peaks were defined with the threshold of log2-fold change of 1 or 2 and an adjusted *p*-value < 0.05^54^. PCA was performed using princomp in R.

Cis-regulatory Element Annotation System (CEAS) analysis is used to annotate resulting peaks with genome features. Cistrome Toolkit (dbtoolkit.cistrome.org) was used to probe which factors might regulate the user-defined genes. GREAT was used to annotate peaks with their biological functions. Conservation plots were obtained with the Conservation Plot (version 1.0.0) tool available in Cistrome^52,53^.

For all motif analyses, HOMER was used to generate a list of the most enriched motifs. Subsequently, *K*-means clustering is applied based on the correlation coefficients of position-specific weight matrix for each motif and the final results are ranked based on the smallest *p*-value in each cluster.

#### Analysis of SEs

Bed files for H3K27ac peaks created by MACS2 were used as input to by ROSE^52^ to call SEs in H3K27ac ChIP-seq data.

#### Visualization of ChIP-seq and ATAC-seq data

Read depth-normalized profiles corresponding to read coverage per one million reads were used for heatmaps and for visualization using the integrative genomics viewer^55^. Heatmaps were prepared using deepTools (version 2.5.4) and aggregation plots for ChIP-seq signals were generated using Sitepro in CEAS^56^. In the volcano plots, ATAC-seq peak summits were associated with the nearest transcription start site (TSS) within a distance of ±50 kb and incorporating DESeq2 output from RNA-seq, with the final plot generated using ggplot2 in R.

#### Analysis and visualization of RNA-seq data

For RNA-seq data, read alignment, quality control, and data analysis were performed using VIPER^57^. RNA-seq reads were mapped by STAR^58^ to hg19 and read counts for each gene were generated by Cufflinks. Differential gene expression analyses were performed on absolute gene counts for RNA-seq data using DESeq2. The top 50 genes scored by multiplying the log2-fold change by the −log(*p*-value) that were near ATAC-seq peaks were used as signatures for the ASCL1 and NEUROD1 subtypes. These were applied to the Beltran et al.^33^ and Labrecque et al.^16^ NEPC cohorts to get signature scores by GSVA software^59^ for each subtype. The difference in these scores was plotted against a normalized ASCL1/NEUROD1 expression ratio in Supplementary Fig. 3b. Specifically, the *x*-axis shows the differential enrichment of GSVA signature scores calculated using a Kolmogorov–Smirnov (KS) rank statistic yielding single-sample enrichment scores that are dependent on the sample set^59^. The *y*-axis is the ratio of ASCL1/NEUROD1 expression levels normalized by subtracting the mean and dividing by the SD. The rank correlation (Spearman) between these values was 0.57 (*p*-value of 0.01).

#### Single-cell ATAC-seq and RNA-seq

Single-cell RNA-seq data generated by 10× Genomics were preprocessed using the Cell Ranger (https://www.10xgenomics.com/) to obtain the UMI (unique molecular identifier) counts for each gene. To get a reliable single-cell transcriptome data set, we excluded the cells with <200 genes expressed (UMI > 0) or the cells with >80% UMIs from mitochondrial genes. The filtered data were then normalized and scaled by using Seurat^34^ to remove unwanted sources of variations. tSNE was performed on the normalized data to visualize the single cells in two-dimensional space by using the top ten dimensions of PCA. Unsupervised clustering was performed by using the “FindClusters” function in the Seurat package with parameter of resolution = 0.8. Cell cycle phases of all single cells were assigned by using the cyclone function in scran package^60^. Genes with differential expression between clusters were obtained by using Wilcoxon rank-sum test. FDR was then calculated to correct for multiple testing.

Single-cell ATAC-seq data were processed using the Cell Ranger ATAC pipeline v1.1.0, which provides quality control (QC) and clustering. Any cell that had FrIP <0.2 or total fragments <1000 was removed from the analysis. The tSNE analysis was performed using the implementation from the Loupe Cell Browser 3.1.0.

scATAC-seq and scRNA-seq data integration was performed by Seurat. The scATAC-seq peak matrix provided by 10× was loaded and collapsed to a “gene activity matrix.” The processed data was then scaled and normalized. To help understand the internal structure of the ATAC-seq data, the “RunLSI” function was run. “FindTransferAnchors” function identifies “anchors” between the ATAC-seq and RNA-seq data sets, and finally ATAC-seq and RNA-seq data are able to be co-embedded in the same tSNE plot.

#### Single-cell CNV

By modifying an existing method used for bulk ATAC-seq data, we created a way to use off-target scATAC-seq reads to infer DNA copy number amplifications. This approach first breaks the genome into many large intervals and finds the coverage of each window. The coverage of 100 GC-matched intervals are then averaged together as background. The coverage of each interval will be compared to each GC-matched background to estimate CNV fold change. The size of each interval was set to 1–2 Mb, to account for the sparsity of the scATAC-seq data with “ChunkGRanges” function in GenomicRange. For each window, the “GCcontent” function of biovizBase was used to calculate the percentage GC content. The coverage was compensated for removed peaks by using the effective window size in coverage calculation.

#### Whole-exome sequencing

Reads were aligned using BWA v0.5.9 and somatic mutations called using a customized version of the Getz Lab CGA WES Characterization pipeline (https://portal.firecloud.org/methods/getzlab/CGA_WES_Characterization_Pipeline_v0.1_Dec2018/). We used ContEst^61^ to estimate cross-sample contamination, MuTect^62^ v1.1.6 to call single nucleotide variants, and Strelka^63^ v1.0.11 to call indels. MuTect2.1^64^ was used to confirm Strelka indel calls. We applied DeTiN^65^ to rescue true somatic variants that were removed due to tumor-in-normal contamination. Variant calls were filtered through a panel of normal samples to remove artifacts from miscalled germline alterations and other rare error modes. Variants were annotated using VEP, Oncotator, and vcf2maf v1.6.17 (https://github.com/mskcc/vcf2maf). Allelic copy number, tumor purity, and ploidy were analyzed using ABSOLUTE^66^.

Prior to characterizing somatic mutations and copy number profiles from PDX samples, we removed potentially confounding mouse DNA sequences using ConcatRef^67^. Briefly, WES results were aligned to a concatenated hg19 reference genome and only reads for which both pairs uniquely aligned to just the hg19 reference sequences using BWA. The resultant high-confidence human paired-end sequences were then used for downstream analysis as above.

### Reporting summary

Further information on research design is available in the Nature Research Reporting Summary linked to this article.

## Supplementary information

Supplementary Information

Description of Additional Supplementary Files

Supplementary Data 1

Supplementary Data 2

Reporting Summary

## Data Availability

The ATAC-seq, ChIP-seq, and RNA-seq data generated in this study have been deposited in the NCBI GEO database under accession code GSE156292. The publicly available data used in Supplementary Fig. 1 were downloaded from the NCBI GEO database under accession number GSE118207. The publicly available RNA-seq data used in Fig. 3a and Supplementary Fig. 3a, b were downloaded from dbGap phs000909.v.p1 https://www.ncbi.nlm.nih.gov/projects/gap/cgi-bin/study.cgi?study_id=phs000909.v1.p1 and from the NCBI GEO database under accession number GSE126078. Source data are provided with this paper.

## Source data

Source Data
