# Supplementary material for: Subtype heterogeneity and epigenetic convergence in neuroendocrine prostate cancer
Source: Nat Commun. 2021 Oct 1;12:5775. doi: 10.1038/s41467-021-26042-z (PMC8486778; doi:10.1038/s41467-021-26042-z)
Supplement: Supplementary file 1 — Supplementary Information [file 41467_2021_26042_MOESM1_ESM.pdf]

Suppl. Fig1

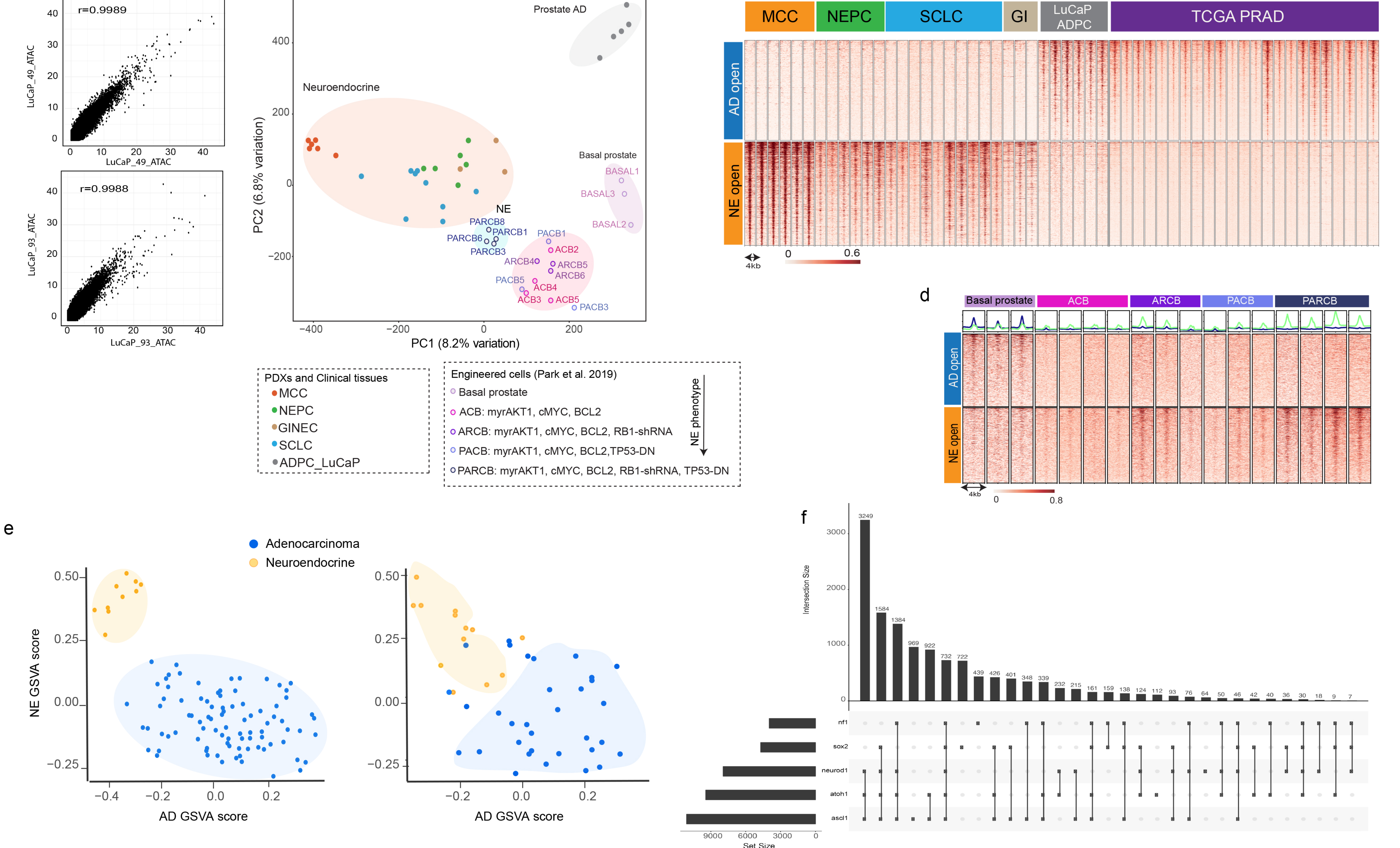

a
r=0.9989
LuCaP_49_ATAC
LuCaP_49_ATAC
r=0.9988
LuCaP_93_ATAC
LuCaP_93_ATAC
b
Prostate AD
Neuroendocrine
Basal prostate
BASAL1
BASAL3
BASAL2
NE
PARCB8
PARCB1
PARCB6
PARCB3
PACB1
ACB2
ARCB4
ARCB5
ARCB6
PACB5
ACB4
ACB3
ACB5
PACB3
PC2 (6.8% variation)
PC1 (8.2% variation)
PDXs and Clinical tissues
MCC
NEPC
GINEC
SCLC
ADPC_LuCaP
Engineered cells (Park et al. 2019)
Basal prostate
ACB: myrAKT1, cMYC, BCL2
ARCB: myrAKT1, cMYC, BCL2, RB1-shRNA
PACB: myrAKT1, cMYC, BCL2,TP53-DN
PARCB: myrAKT1, cMYC, BCL2, RB1-shRNA, TP53-DN
NE phenotype
c
MCC
NEPC
SCLC
GI
LuCaP ADPC
TCGA PRAD
AD open
NE open
4kb
0
0.6
d
Basal prostate
ACB
ARCB
PACB
PARCB
AD open
NE open
4kb
0
0.8
e
Adenocarcinoma
Neuroendocrine
NE GSVA score
AD GSVA score
AD GSVA score
f
Intersection Size
nf1
sox2
neurod1
atoh1
ascl1
Set Size

Supplemental Figure 1. NE carcinomas share a common chromatin state independent of their anatomical origin. (**a**) Correlation plots of the ATAC-seq signal for two LuCaP_49 replicates (Pearson's correlation r = 0.9989, left) and LuCaP_93 replicates (Pearson's correlation r = 0.9988, right). (**b**) PCA analysis of ATAC-seq data including neuroendocrine samples (MCC, NEPC, GINE, SCLC), ADPC LuCaPs and engineered prostate cells from a published study[16] . Those cells cover a spectrum of transdifferentiation from the basal prostate epithelium through intermediate stages toward a neuroendocrine phenotype with the alterations indicated in the legend. The final cell line samples ('PARCB', expressing dominant negative TP53 (TP53DN), myrAKT1, RB1-shRNA, c-MYC, and BCL2) cluster in close proximity to the NEPC, GINE and SCLC samples from our analysis. (**c**) Heatmap representation of the differential regions between ADs and NECs for the complete collection of NECs analyzed in our studies compared to the LuCaP ADPC and the TCGA primary prostate adenocarcinomas (PRAD). Each row is a peak location and each column is a sample. (**d**) Heatmap representation of the differential regions between ADs and NECs for the engineered cells from Park et al [16]. Each row is a peak location and each column is a sample. Shown above each column are the composite tag density plots for the AD sites (black) and NE sites (green). (e) Plot of patient RNA-seq data scored by NE (y-axis) and AD (x-axis) signatures derived from the ATAC-seq sites in Figure 1c. The left plot is data from Labreque et. al.[17] the right plot is data from Beltran et. al[34]. (**f**) UpSetR plot showing the intersections of ATAC-seq peaks containing the specified motifs and their intersection sizes.

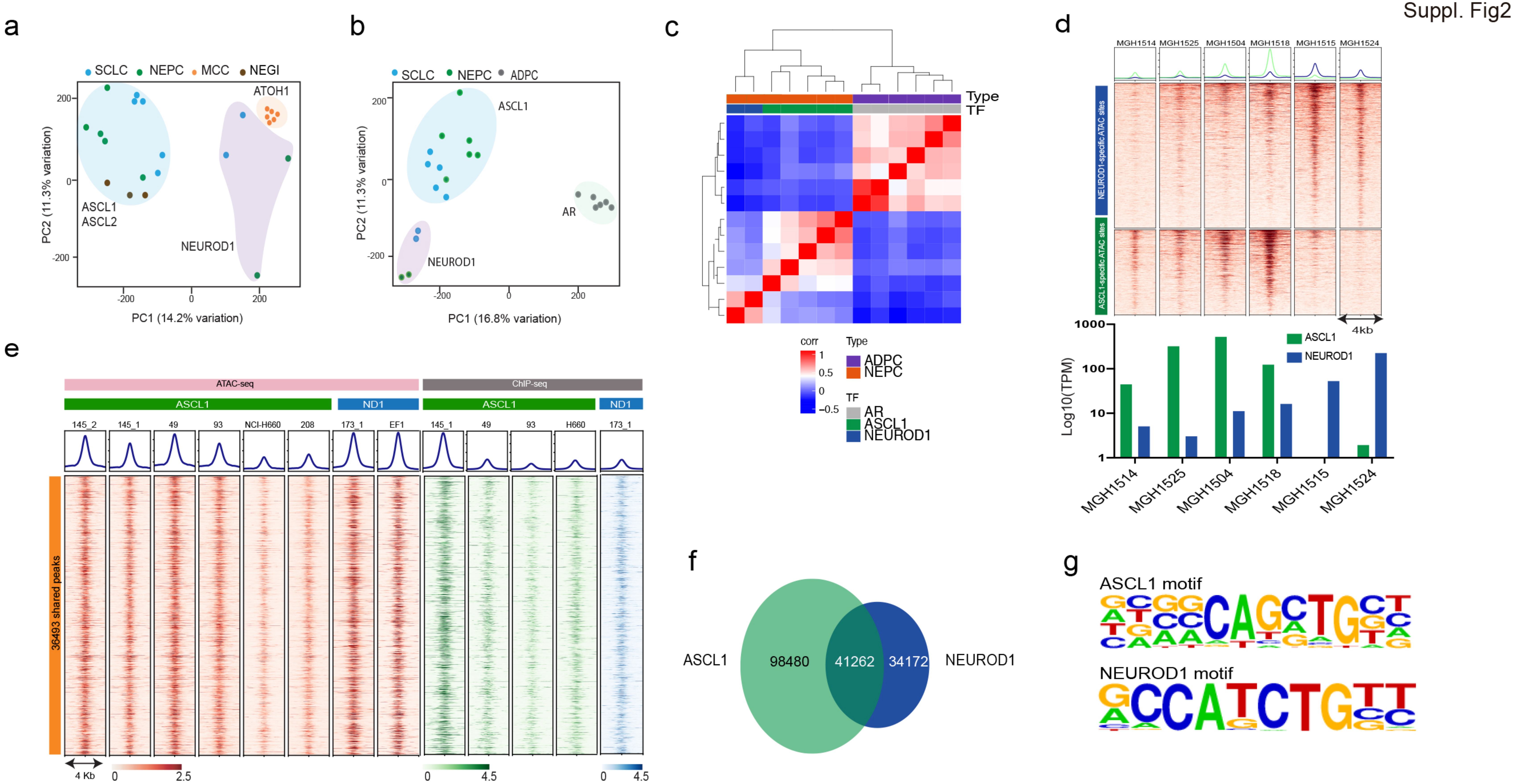

Suppl. Fig2
a
SCLC
NEPC
MCC
NEGI
ATOH1
ASCL1
ASCL2
NEUROD1
PC1 (14.2% variation)
PC2 (11.3% variation)
b
SCLC
NEPC
ADPC
ASCL1
AR
NEUROD1
PC1 (16.8% variation)
PC2 (11.3% variation)
c
Type
TF
corr
ADPC
NEPC
AR
ASCL1
NEUROD1
d
MGH1514
MGH1525
MGH1504
MGH1518
MGH1515
MGH1524
NEUROD1-specific ATAC sites
ASCL1-specific ATAC sites
4kb
Log10(TPM)
e
ATAC-seq
ChIP-seq
ASCL1
ND1
36493 shared peaks
4 Kb
f
ASCL1
98480
41262
34172
NEUROD1
g
ASCL1 motif
NEUROD1 motif

Supplemental Figure 2. NEPC shows tumor subtypes based on the differential expression of the transcription factors ASCL1 and NEUROD1. **(a)** PCA analysis of ATAC-seq data of NECs, samples are color coded by tumor type and clusters are highlighted based on the expression of the dominant bHLH TF in each sample. (**b**) PCA analysis of ATAC-seq data of SCLC, NEPC and ADPC, samples are color coded by tumor type and clusters are highlighted based on the expression of the dominant bHLH TF in each sample. (**c**) Hierarchical clustering of the pairwise Pearson's correlation of the RNA-seq signal across the NEPCs and ADPC. (**d**) Heatmap of the ATAC-seq signal in SCLCs at the ASCL1- and NEUROD1-specific DNA accessible regions identified in NEPC (on top) and corresponding expression of ASCL1 and NEUROD1 of the same SCLC tissues (bottom). (**e**) Heatmap of the ATAC-seq signal at the shared accessible regions in the ASCL1 and NEUROD1 NEPC subtypes (36493 peaks that are shared in at least four samples). Right side of the plot shows the ChIP-seq results for ASCL1 (green) and NEUROD1 (blue) for the indicated samples at the same sites. (**f)** Venn diagram representing the union of the ASCL1 binding sites obtained by ChIP-seq in PDXs (145.1, 93, 43 and H660) in red and the NEUROD1 bindings by ChIP-seq analysis in PDX 173.1 (in blue) (**g**) Top enriched consensus motifs identified in ASCL1 ChIP-seq (top) and NEUROD1 ChIP-seq data (bottom). Source data are provided as a Source Data file.

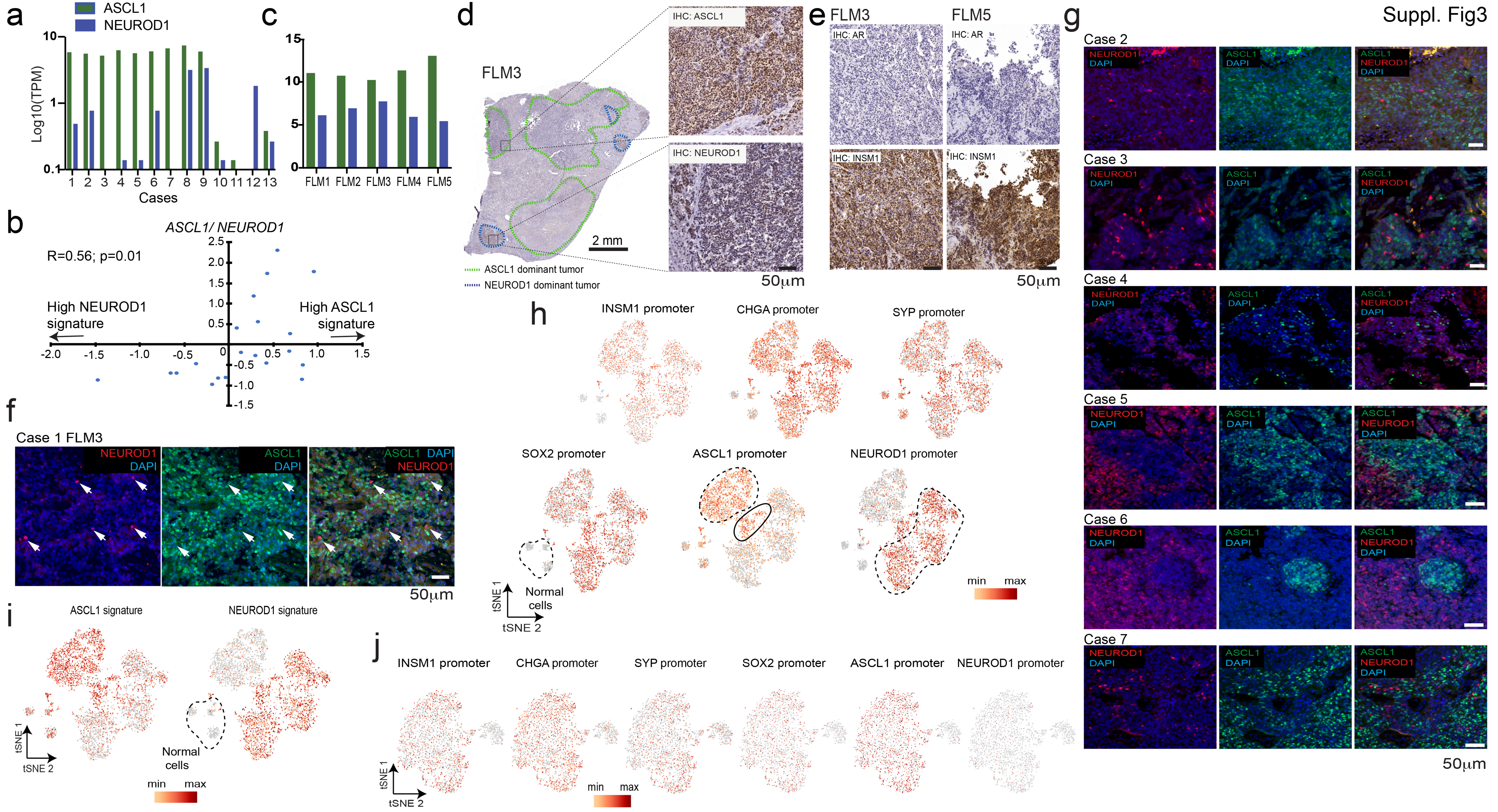
Suppl. Fig3
a
ASCL1
NEUROD1
Log10(TPM)
Cases
b
ASCL1/ NEUROD1
R=0.56; p=0.01
High NEUROD1 signature
High ASCL1 signature
c
FLM1 FLM2 FLM3 FLM4 FLM5
d
FLM3
IHC: ASCL1
IHC: NEUROD1
2 mm
ASCL1 dominant tumor
NEUROD1 dominant tumor
50μm
e
FLM3
FLM5
IHC: AR
IHC: INSM1
50μm
f
Case 1 FLM3
NEUROD1 DAPI
ASCL1 DAPI
ASCL1 DAPI NEUROD1
50μm
g
Case 2
Case 3
Case 4
Case 5
Case 6
Case 7
NEUROD1 DAPI
ASCL1 DAPI
ASCL1 NEUROD1 DAPI
50μm
h
INSM1 promoter
CHGA promoter
SYP promoter
SOX2 promoter
ASCL1 promoter
NEUROD1 promoter
Normal cells
tSNE 1
tSNE 2
min max
i
ASCL1 signature
NEUROD1 signature
Normal cells
tSNE 1
tSNE 2
min max
j
INSM1 promoter
CHGA promoter
SYP promoter
SOX2 promoter
ASCL1 promoter
NEUROD1 promoter
tSNE 1
tSNE 2
min max

Supplemental Figure 3. Single cell analysis reveals that NEPC sub-types co-exist in human metastasis and contribute to inter- and intra-tumoral heterogeneity. (a) Plot of ASCL1 and NEUROD1 expression in NEPC tissues from a clinical cohort (Beltran et al. 2016). (**b**) Scatter plot showing correlation between scores for gene expression signatures assessed by GSVA analysis[60], and expression of *ASCL1* or *NEUROD1* for the clinical samples in the Beltran and Labrecque cohorts. The resulting values show a spearman correlation of 0.57 (p=0.01) indicating that the signatures are ranking the samples in accordance with the amount of ASCL1 and NEUROD1 expressing cells. (**c**) Plot of ASCL1 and NEUROD1 expression in the five fragments of liver metastases from case 1. (**d**) IHC analysis of a whole FLM3 section at low magnification showing distinct foci for ASCL1 expressing cells (circled in green) and NEUROD1 expressing cells (circled in blue). The right-hand side of the panel has high magnification micrographs showing regions of ASCL1 positive (top) and NEUROD1 positive (bottom) tumor cells. (**e**) Immunohistochemical analysis of AR (top) and INSM1 (bottom) of FLM3 and FLM5 tissues. (**f**) Representative immunofluorescence staining of FLM3 (case 1) for ASCL1 (green) and NEUROD1 (red), white arrows are pointing at NEUROD1 expressing tumor cells showing no ASCL1 expression. (**g**) Immunofluorescence staining of six additional NEPC metastatic samples for ASCL1 (green), NEUROD1 (red) and nuclear staining DAPI (blue). Cases 2-5 are patient samples, case 6 is an organoid model grown in vivo (PDOX) and case 7 is LuCaP 208. (**h**) t-SNE analysis of the scATACseq data of FLM3, showing accessibility at the SOX2 promoter (marking tumor cells) and accessibility at INSM1, CHGA and SYP promoters (NE markers). ASCL1 (left) and NEUROD1 (right) open promoters mark ASCL1 and NEUROD1 subpopulations within the dashed lines. A small “mixed” cluster consists of cells that are open for either ASCL1 or NEUROD1 but are intermixed (solid line). (**i**) t-SNE analysis of the scATAC-seq data of FLM3 showing accessibility at the top 30 differential ATAC-seq regions identified by bulk analysis in ASCL1 (top) and NEUROD1 (bottom) subtypes. (**j**) t-SNE analysis of the scATAC-seq data of FLM5, showing accessibility at the SOX2 promoter, accessibility at INSM1 (NE marker) and NEUROD1 (left) and ASCL1 (right) promoters marking NEUROD1 and ASCL1 subpopulations respectively. Source data are provided as a Source Data file.

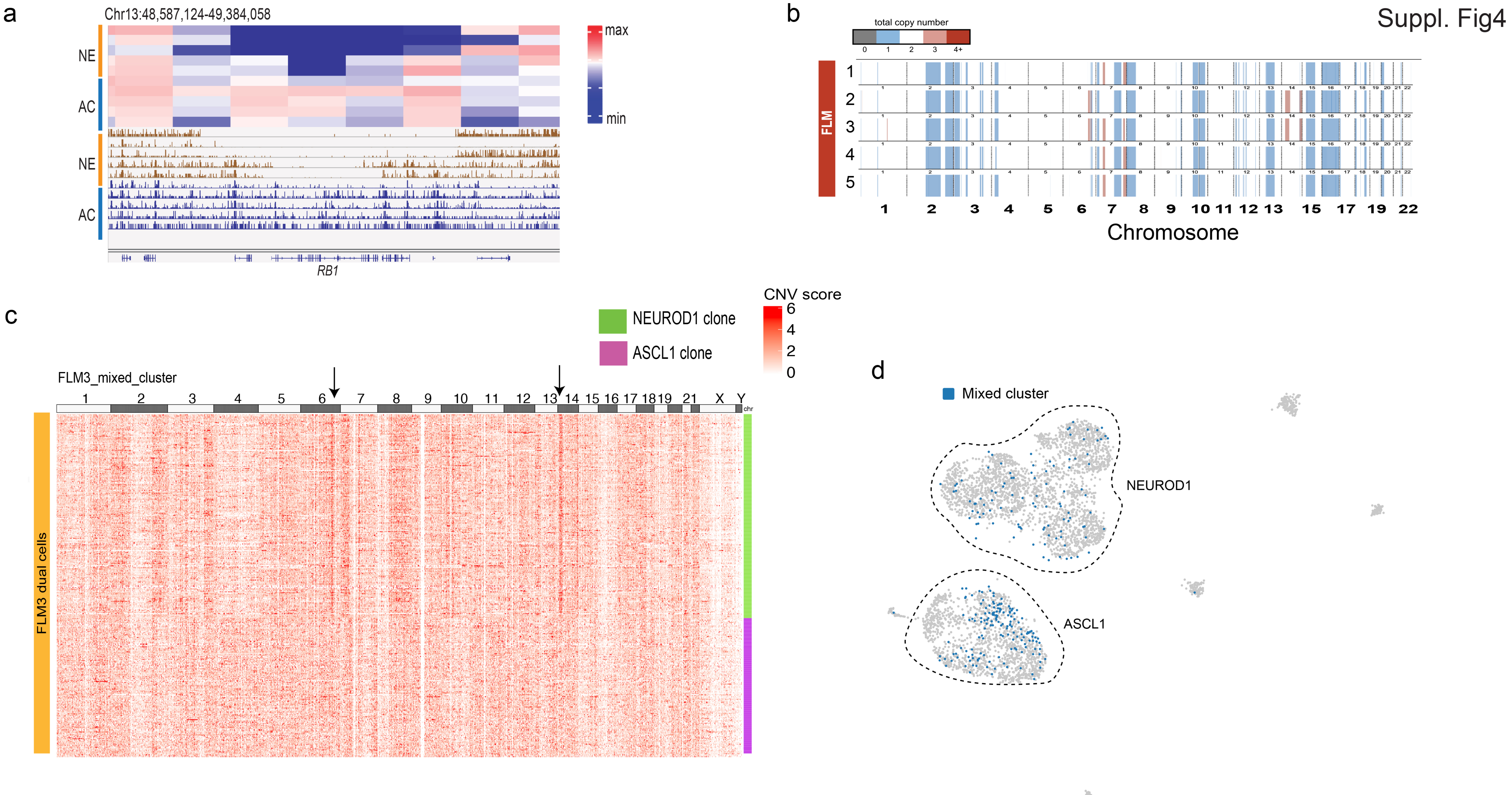
Suppl. Fig4
a
Chr13:48,587,124-49,384,058
NE
AC
NE
AC
max
min
RB1
b
total copy number
0
1
2
3
4+
FLM
1
2
3
4
5
1 2 3 4 5 6 7 8 9 10 11 12 13 15 17 19 22
Chromosome
c
NEUROD1 clone
ASCL1 clone
CNV score
6
4
2
0
FLM3_mixed_cluster
1 2 3 4 5 6 7 8 9 10 11 12 13 14 15 16 17 18 19 21 X Y
chr
FLM3 dual cells
d
Mixed cluster
NEUROD1
ASCL1

Supplemental Figure 4. The NEPC sub-types are distinct clones. (a) Inference of CNV at RB1 locus from ATAC-seq bulk signal in NEPC and ADPC. (top) Heatmap representation of the inferred data and (bottom) IGV track of the actual ATAC-seq data at that locus. (**b**) Heatmap of the CNV alterations determined by WES across FLM3 (by clusters) and FLM5. (**c**) Heatmap of the single cell CNV analysis of the mixed cluster of ASCL1 and NEUROD1. Two dominant clusters are evident that are the same as the larger clusters of ASCL1 and NEUROD1 subclones. (**d**) UMAP of the single cell ATAC-seq data of FLM3 showing that all cells from the mixed cluster of Supp Fig. 3h resolve into either the ASCL1 and NEUROD1 cluster in this analysis. Source data are provided as a Source Data file.

Supplemental Table1. Tumor samples used for the ATAC-seq analysis

Sample	Tumor	Tissue	ATAC-seq				RNA-seq
			UniquelyMapped	Total_Peaks	DHS	FRiP	
DFMC_11112	MCC	PDX	45497753	91671	4897	36.4	Yes
DFMC_33043	MCC	PDX	50628567	90443	4828	41.1	Yes
DFMC_48396	MCC	PDX	56332609	94578	4871	34.8	Yes
DFMC_63632	MCC	PDX	44959450	66762	4671	26.7	Yes
DFMC_87346	MCC	PDX	47845776	61516	4887	38.6	Yes
DFMC_96712	MCC	PDX	44559386	72993	4832	29.4	Yes
EF1_cfce	NEPC	Cell line	95546177	76884	4890	39.1	
LuCaP_145_1_rep1	NEPC	PDX	27578828	25268	4806	12.6	
LuCaP_145_1_rep2	NEPC	PDX	40326914	41537	4819	4.6	
LuCaP_145_2	NEPC	PDX	21372235	32372	4892	16.6	Yes
LuCaP_173_1_p17	NEPC	PDX	37204748	31379	4828	33.3	Yes
LuCaP_173_1_p1	NEPC	PDX	173962297	79631	4782	8	Yes
LuCaP_208	NEPC	PDX	53527713	36861	4859	9	
LuCaP_49_rep1	NEPC	PDX	40159074	35772	4881	15	Yes
LuCaP_49_rep2	NEPC	PDX	44956639	51883	4893	21	
LuCaP_23	ADPC	PDX	30997298	49662	4868	24	Yes
LuCaP_77	ADPC	PDX	19527592	54493	4731	26.3	Yes
LuCaP_77CR	ADPC	PDX	52410587	56397	4838	20.4	
LuCaP_78	ADPC	PDX	29173802	48785	4641	17.6	Yes
LuCaP_81	ADPC	PDX	47086721	40311	4784	13.5	Yes
LuCaP_93_rep1	ADPC	PDX	39109506	35158	4664	10.5	Yes
LuCaP_93_rep2	ADPC	PDX	33565564	42470	4638	14.2	
LuCaP_96	ADPC	PDX	66307812	51946	4926	18.1	Yes
MGH_1504	SCLC	PDX	37791305	35413	4628	13.6	Yes
MGH_1514	SCLC	PDX	62263801	37249	4649	8.9	Yes
MGH_1515	SCLC	PDX	35865754	32243	4465	11.1	Yes
MGH_1518	SCLC	PDX	33289869	38847	4472	15.6	Yes
MGH_1524-1	SCLC	PDX	45397760	25676	4704	5.7	Yes
MGH_1525	SCLC	PDX	42628509	27972	4758	6.8	Yes
MGH_1545	SCLC	PDX	35339906	51209	4697	22	Yes
MGH_1567-1	SCLC	PDX	58821999	21845	4910	5.8	
NCI-H660	NEPC	Cell line	310339362	70104	4423	15.1	
T17-11572_NEC	GINEC	Primary tissue	62189628	54148	4959	22.6	Yes
T18-01479_NEC	GINEC	Primary tissue	52641558	80490	4853	30	Yes
T18-05426_I_NEC	GINEC	Primary tissue	58703600	55989	4896	36.2	Yes

DHS: DNase I hypersensitive site (per 5000 peaks)

FRIP: fraction of reads that fall into a peak

Supplemental table 2. Motif analysis at NE-specific peaks

Motif Name	Consensus	P-value	Log P-value	q-value (Benjamini)	# of Target Sequences with Motif(of 4540)	% of Target Sequences with Motif	# of Background Sequences with Motif(of 44756)	% of Background Sequences with Motif
Atoh1(bHLH)/Cerebellum-Atoh1-ChIP-Seq(GSE22111)/Homer	VNRVCAGCTGGY	1e-398	-9.17E+02	0	2781	61.26%	13641.7	30.48%
Ascl1(bHLH)/NeuralTubes-Ascl1-ChIP-Seq(GSE55840)/Homer	NNVVCAGCTGBN	1e-384	-8.85E+02	0	3364	74.10%	19420.5	43.39%
NeuroD1(bHLH)/Islet-NeuroD1-ChIP-Seq(GSE30298)/Homer	GCCATCTGTT	1e-363	-8.38E+02	0	2308	50.84%	10266.3	22.94%
NeuroG2(bHLH)/Fibroblast-NeuroG2-ChIP-Seq(GSE75910)/Homer	ACCATCTGTT	1e-328	-7.57E+02	0	3056	67.31%	17367	38.80%
HEB(bHLH)/mES-Heb-ChIP-Seq(GSE53233)/Homer	VCAGCTGBNN	1e-326	-7.52E+02	0	3542	78.02%	22448.3	50.16%
E2A(bHLH)/proBcell-E2A-ChIP-Seq(GSE21978)/Homer	DNRCAGCTGY	1e-324	-7.48E+02	0	3173	69.89%	18591.1	41.54%
Olig2(bHLH)/Neuron-Olig2-ChIP-Seq(GSE30882)/Homer	RCCATMTGTT	1.00E-291	-6.71E+02	0	3228	71.10%	19821.1	44.29%
Ptf1a(bHLH)/Panc1-Ptf1a-ChIP-Seq(GSE47459)/Homer	ACAGCTGTTN	1.00E-290	-6.69E+02	0	3899	85.88%	27464	61.37%
MyoG(bHLH)/C2C12-MyoG-ChIP-Seq(GSE36024)/Homer	AACAGCTG	1.00E-266	-6.15E+02	0	2494	54.93%	13402.9	29.95%
Tcf12(bHLH)/GM12878-Tcf12-ChIP-Seq(GSE32465)/Homer	VCAGCTGYTG	1.00E-262	-6.04E+02	0	2448	53.92%	13095.1	29.26%
Tcf21(bHLH)/ArterySmoothMuscle-Tcf21-ChIP-Seq(GSE61369)/Ho	NAACAGCTGG	1.00E-258	-5.95E+02	0	2335	51.43%	12205.7	27.27%
Myf5(bHLH)/GM-Myf5-ChIP-Seq(GSE24852)/Homer	BAACAGCTGT	1.00E-240	-5.55E+02	0	1900	41.85%	9022.1	20.16%
MyoD(bHLH)/Myotube-MyoD-ChIP-Seq(GSE21614)/Homer	RRCAGCTGYTSY	1.00E-212	-4.89E+02	0	1986	43.74%	10214.4	22.82%
Ap4(bHLH)/AML-Tfap4-ChIP-Seq(GSE45738)/Homer	NAHCAGCTGD	1.00E-206	-4.75E+02	0	2524	55.59%	14927.7	33.35%
Lhx2(Homeobox)/HFSC-Lhx2-ChIP-Seq(GSE48068)/Homer	TAATTAGN	1.00E-178	-4.11E+02	0	1694	37.31%	8557.6	19.12%
Lhx1(Homeobox)/EmbryoCarcinoma-Lhx1-ChIP-Seq(GSE70957)/H	NNYTAATTAR	1.00E-169	-3.90E+02	0	1715	37.78%	8904	19.90%
Slug(Zf)/Mesoderm-Snai2-ChIP-Seq(GSE61475)/Homer	SNGCACCTGCHS	1.00E-166	-3.83E+02	0	1590	35.02%	7995.8	17.87%
Lhx3(Homeobox)/Neuron-Lhx3-ChIP-Seq(GSE31456)/Homer	ADBTAATTAR	1.00E-166	-3.83E+02	0	2166	47.71%	12695.4	28.37%
NF1(CTF)/LNCAP-NF1-ChIP-Seq(Unpublished)/Homer	CYTGGCABNSTGCCAR	1.00E-162	-3.75E+02	0	1214	26.74%	5314.3	11.87%
E2A(bHLH),near_PU.1/Bcell-PU.1-ChIP-Seq(GSE21512)/Homer	NVCACCTGBN	1.00E-148	-3.43E+02	0	2676	58.94%	17791.2	39.75%
Nkx6.1(Homeobox)/Islet-Nkx6.1-ChIP-Seq(GSE40975)/Homer	GKTAATGR	1.00E-135	-3.12E+02	0	2758	60.75%	18977	42.40%
NF1-halfsite(CTF)/LNCaP-NF1-ChIP-Seq(Unpublished)/Homer	YTGCCAAG	1.00E-131	-3.04E+02	0	2838	62.51%	19872.4	44.40%
SCL(bHLH)/HPC7-Scl-ChIP-Seq(GSE13511)/Homer	AVCAGCTG	1.00E-121	-2.79E+02	0	4232	93.22%	36268	81.04%
Tgif2(Homeobox)/mES-Tgif2-ChIP-Seq(GSE55404)/Homer	TGTCANYT	1.00E-101	-2.34E+02	0	3460	76.21%	27394.9	61.21%
Tlx?(NR)/NPC-H3K4me1-ChIP-Seq(GSE16256)/Homer	CTGGCAGSCTGCCA	1.00E-99	-2.28E+02	0	1114	24.54%	5775.3	12.90%
Isl1(Homeobox)/Neuron-Isl1-ChIP-Seq(GSE31456)/Homer	CTAATKGV	1.00E-93	-2.14E+02	0	2285	50.33%	15853	35.42%
ZEB1(Zf)/PDAC-ZEB1-ChIP-Seq(GSE64557)/Homer	VCAGGTRDRY	1.00E-92	-2.12E+02	0	2701	59.49%	19857.5	44.37%
Pit1+1bp(Homeobox)/GCrat-Pit1-ChIP-Seq(GSE58009)/Homer	ATGCATAATTCA	1.00E-75	-1.73E+02	0	587	12.93%	2521.5	5.63%
Tbx5(T-box)/HL1-Tbx5.biotin-ChIP-Seq(GSE21529)/Homer	AGGTGTCA	1.00E-74	-1.72E+02	0	3473	76.50%	28575.3	63.85%
Meis1(Homeobox)/MastCells-Meis1-ChIP-Seq(GSE48085)/Homer	VGCTGWCAVB	1.00E-66	-1.54E+02	0	2238	49.30%	16415.6	36.68%
ZBTB18(Zf)/HEK293-ZBTB18.GFP-ChIP-Seq(GSE58341)/Homer	AACATCTGGA	1.00E-64	-1.49E+02	0	1054	23.22%	6173.1	13.79%
Tgif1(Homeobox)/mES-Tgif1-ChIP-Seq(GSE55404)/Homer	YTGWCADY	1.00E-60	-1.39E+02	0	3119	68.70%	25409.9	56.78%
Sox6(HMG)/Myotubes-Sox6-ChIP-Seq(GSE32627)/Homer	CCATTGTTNY	1.00E-57	-1.32E+02	0	1794	39.52%	12720.4	28.42%
Pdx1(Homeobox)/Islet-Pdx1-ChIP-Seq(SRA008281)/Homer	YCATYAATCA	1.00E-56	-1.30E+02	0	1237	27.25%	7919.4	17.70%
Nanog(Homeobox)/mES-Nanog-ChIP-Seq(GSE11724)/Homer	RGCCATTAAC	1.00E-55	-1.29E+02	0	3659	80.59%	31453.8	70.28%
Sox3(HMG)/NPC-Sox3-ChIP-Seq(GSE33059)/Homer	CCWTTGTY	1.00E-51	-1.18E+02	0	1921	42.31%	14152.9	31.62%
Bapx1(Homeobox)/VertebralCol-Bapx1-ChIP-Seq(GSE36672)/Hom	TTRAGTGSYK	1.00E-50	-1.16E+02	0	2356	51.89%	18272.6	40.83%
Foxo1(Forkhead)/RAW-Foxo1-ChIP-Seq(Fan_et_al.)/Homer	CTGTTTAC	1.00E-48	-1.11E+02	0	2159	47.56%	16511.4	36.89%
Smad4(MAD)/ESC-SMAD4-ChIP-Seq(GSE29422)/Homer	VBSYGTCTGG	1.00E-46	-1.08E+02	0	2053	45.22%	15572.2	34.79%
Sox2(HMG)/mES-Sox2-ChIP-Seq(GSE11431)/Homer	BCCATTGTTC	1.00E-46	-1.07E+02	0	1116	24.58%	7264.3	16.23%
Nkx2.1(Homeobox)/LungAC-Nkx2.1-ChIP-Seq(GSE43252)/Homer	RSCACTYRAG	1.00E-46	-1.07E+02	0	2961	65.22%	24486.1	54.71%
OCT:OCT-short(POU,Homeobox)/NPC-OCT6-ChIP-Seq(GSE43916)/	ATGCATWATGCATRW	1.00E-45	-1.05E+02	0	889	19.58%	5436.9	12.15%
AP-2gamma(AP2)/MCF7-TFAP2C-ChIP-Seq(GSE21234)/Homer	SCCTSAGGSCAW	1.00E-43	-1.01E+02	0	1859	40.95%	13932.1	31.13%
Nkx2.2(Homeobox)/NPC-Nkx2.2-ChIP-Seq(GSE61673)/Homer	BTBRAGTGSN	1.00E-43	-1.01E+02	0	2343	51.61%	18499.1	41.33%
EBF1(EBF)/Near-E2A-ChIP-Seq(GSE21512)/Homer	GTCCCCWGGGGA	1.00E-43	-9.99E+01	0	1934	42.60%	14642.8	32.72%
Sox10(HMG)/SciaticNerve-Sox3-ChIP-Seq(GSE35132)/Homer	CCWTTGTYYB	1.00E-42	-9.84E+01	0	1834	40.40%	13751.8	30.73%
Smad2(MAD)/ES-SMAD2-ChIP-Seq(GSE29422)/Homer	CTGTCTGG	1.00E-42	-9.72E+01	0	1977	43.55%	15103	33.75%
Smad3(MAD)/NPC-Smad3-ChIP-Seq(GSE36673)/Homer	TWGTCTGV	1.00E-41	-9.62E+01	0	3041	66.98%	25557.4	57.11%
Nkx2.5(Homeobox)/HL1-Nkx2.5.biotin-ChIP-Seq(GSE21529)/Home	RRSCACTYAA	1.00E-41	-9.51E+01	0	2515	55.40%	20300.3	45.36%
AR-halfsite(NR)/LNCaP-AR-ChIP-Seq(GSE27824)/Homer	CCAGGAACAG	1.00E-40	-9.42E+01	0	3619	79.71%	31749.7	70.94%
Sox4(HMG)/proB-Sox4-ChIP-Seq(GSE50066)/Homer	YCTTTGTTCC	1.00E-40	-9.41E+01	0	1086	23.92%	7227.7	16.15%
OCT:OCT(POU,Homeobox,IR1)/NPC-Brn2-ChIP-Seq(GSE35496)/Ho	ATGAATWATTCATGA	1.00E-40	-9.28E+01	0	79	1.74%	106.6	0.24%
FoxL2(Forkhead)/Ovary-FoxL2-ChIP-Seq(GSE60858)/Homer	WWTRTAAACAVG	1.00E-39	-8.99E+01	0	949	20.90%	6154.1	13.75%
EBF(EBF)/proBcell-EBF-ChIP-Seq(GSE21978)/Homer	DGTCCCYRGGGA	1.00E-38	-8.96E+01	0	553	12.18%	3031.5	6.77%
AP-2alpha(AP2)/Hela-AP2alpha-ChIP-Seq(GSE31477)/Homer	ATGCCCTGAGGC	1.00E-37	-8.61E+01	0	1526	33.61%	11217.3	25.06%
Prop1(Homeobox)/GHFT1-PROP1.biotin-ChIP-Seq(GSE77302)/Hor	NTAATBNAATTA	1.00E-37	-8.58E+01	0	733	16.15%	4467.3	9.98%
Sox15(HMG)/CPA-Sox15-ChIP-Seq(GSE62909)/Homer	RAACAATGGN	1.00E-37	-8.56E+01	0	1263	27.82%	8900	19.89%
Twist(bHLH)/HMLE-TWIST1-ChIP-Seq(Chang_et_al)/Homer	VCAKCTGGNNNCCAGN	1.00E-36	-8.51E+01	0	382	8.41%	1847.6	4.13%
Six2(Homeobox)/NephronProgenitor-Six2-ChIP-Seq(GSE39837)/H	GWAAYHTGAKMC	1.00E-34	-7.93E+01	0	1130	24.89%	7871.9	17.59%
FOXK1(Forkhead)/HEK293-FOXK1-ChIP-Seq(GSE51673)/Homer	NVWTGTTTAC	1.00E-34	-7.92E+01	0	1153	25.40%	8072.8	18.04%
Foxf1(Forkhead)/Lung-Foxf1-ChIP-Seq(GSE77951)/Homer	WWATRTAAACAN	1.00E-33	-7.73E+01	0	979	21.56%	6622.5	14.80%
Rfx6(HTH)/Min6b1-Rfx6.HA-ChIP-Seq(GSE62844)/Homer	TGTTKCCTAGCAACM	1.00E-33	-7.71E+01	0	1551	34.16%	11644.4	26.02%
FOXA1(Forkhead)/LNCAP-FOXA1-ChIP-Seq(GSE27824)/Homer	WAAGTAAACA	1.00E-33	-7.67E+01	0	1244	27.40%	8915.2	19.92%
Hoxc9(Homeobox)/Ainv15-Hoxc9-ChIP-Seq(GSE21812)/Homer	GGCCATAAATCA	1.00E-32	-7.43E+01	0	644	14.19%	3928.1	8.78%
Sox17(HMG)/Endoderm-Sox17-ChIP-Seq(GSE61475)/Homer	CCATTGTTYB	1.00E-32	-7.41E+01	0	896	19.74%	5983.1	13.37%
Fox:Ebox(Forkhead,bHLH)/Panc1-Foxa2-ChIP-Seq(GSE47459)/Hom	NNNVCTGWGYAAACA	1.00E-31	-7.25E+01	0	1245	27.42%	9013	20.14%
RBPJ:Ebox(?,bHLH)/Panc1-Rbpj1-ChIP-Seq(GSE47459)/Homer	GGGRAARRGRMCAGN	1.00E-31	-7.21E+01	0	696	15.33%	4375.2	9.78%
Nkx3.1(Homeobox)/LNCaP-Nkx3.1-ChIP-Seq(GSE28264)/Homer	AAGCACTTAA	1.00E-30	-6.99E+01	0	2371	52.22%	19550.1	43.68%
Foxo3(Forkhead)/U2OS-Foxo3-ChIP-Seq(E-MTAB-2701)/Homer	DGTAAACA	1.00E-30	-6.93E+01	0	859	18.92%	5759.9	12.87%
Six1(Homeobox)/Myoblast-Six1-ChIP-Chip(GSE20150)/Homer	GKVTCADRTTWC	1.00E-29	-6.82E+01	0	371	8.17%	1935.6	4.32%
Phox2a(Homeobox)/Neuron-Phox2a-ChIP-Seq(GSE31456)/Homer	YTAATYNRATTA	1.00E-29	-6.81E+01	0	486	10.70%	2787.4	6.23%
Unknown(Homeobox)/Limb-p300-ChIP-Seq/Homer	SSCMATWAAA	1.00E-29	-6.78E+01	0	767	16.89%	5024.5	11.23%
Barx1(Homeobox)/Stomach-Barx1.3xFlag-ChIP-Seq(GSE69483)/H	AAACMATTAN	1.00E-29	-6.75E+01	0	598	13.17%	3664.3	8.19%
FOXA1(Forkhead)/MCF7-FOXA1-ChIP-Seq(GSE26831)/Homer	WAAGTAAACA	1.00E-29	-6.68E+01	0	1030	22.69%	7259.6	16.22%
Eomes(T-box)/H9-Eomes-ChIP-Seq(GSE26097)/Homer	ATTAACACCT	1.00E-28	-6.61E+01	0	2277	50.15%	18745.6	41.89%
HOXA9(Homeobox)/HSC-Hoxa9-ChIP-Seq(GSE33509)/Homer	GGCCATAAATCA	1.00E-28	-6.59E+01	0	807	17.78%	5388.1	12.04%

PRDM9(Zf)/Testis-DMC1-ChIP-Seq(GSE35498)/Homer	ADGGYAGYAGCATCT	1.00E-28	-6.56E+01	0	769	16.94%	5078.7	11.35%
Pit1(Homeobox)/GCrat-Pit1-ChIP-Seq(GSE58009)/Homer	ATGMATATDC	1.00E-28	-6.49E+01	0	1073	23.63%	7672.5	17.14%
Znf263(Zf)/K562-Znf263-ChIP-Seq(GSE31477)/Homer	CVGTSCTCCC	1.00E-27	-6.42E+01	0	2470	54.41%	20682.3	46.21%
Zic3(Zf)/mES-Zic3-ChIP-Seq(GSE37889)/Homer	GGCCYCCTGCTGDGH	1.00E-27	-6.28E+01	0	1197	26.37%	8805	19.67%
Foxa2(Forkhead)/Liver-Foxa2-ChIP-Seq(GSE25694)/Homer	CYTGTTTACWYW	1.00E-27	-6.23E+01	0	920	20.26%	6410	14.32%
HOXD13(Homeobox)/Chicken-Hoxd13-ChIP-Seq(GSE38910)/Home	NCYAATAAAA	1.00E-26	-6.13E+01	0	1091	24.03%	7907.2	17.67%
Oct6(POU,Homeobox)/NPC-Pou3f1-ChIP-Seq(GSE35496)/Homer	WATGCAAATGAG	1.00E-26	-6.12E+01	0	533	11.74%	3245.5	7.25%
Unknown-ESC-element(?)/mES-Nanog-ChIP-Seq(GSE11724)/Hom	CACAGCAGGGGG	1.00E-24	-5.71E+01	0	1141	25.13%	8445.8	18.87%
Tbr1(T-box)/Cortex-Tbr1-ChIP-Seq(GSE71384)/Homer	AAGGTGTKAA	1.00E-24	-5.65E+01	0	1566	34.49%	12302.5	27.49%
NPAS2(bHLH)/Liver-NPAS2-ChIP-Seq(GSE39860)/Homer	KCCACGTGAC	1.00E-23	-5.51E+01	0	1416	31.19%	10965.8	24.50%
Hoxb4(Homeobox)/ES-Hoxb4-ChIP-Seq(GSE34014)/Homer	TGATTRATGGCY	1.00E-22	-5.28E+01	0	296	6.52%	1560.8	3.49%
FOXK2(Forkhead)/U2OS-FOXK2-ChIP-Seq(E-MTAB-2204)/Homer	SCHTGTTTACAT	1.00E-22	-5.25E+01	0	768	16.92%	5317.8	11.88%
Pitx1(Homeobox)/Chicken-Pitx1-ChIP-Seq(GSE38910)/Homer	TAATCCCN	1.00E-22	-5.25E+01	0	3372	74.27%	30210.4	67.50%
AMYB(HTH)/Testes-AMYB-ChIP-Seq(GSE44588)/Homer	TGGCAGTTGG	1.00E-22	-5.13E+01	0	1630	35.90%	13044.7	29.15%
Zic(Zf)/Cerebellum-ZIC1.2-ChIP-Seq(GSE60731)/Homer	CCTGCTGAGH	1.00E-21	-5.01E+01	0	1294	28.50%	9995	22.33%
FOXP1(Forkhead)/H9-FOXP1-ChIP-Seq(GSE31006)/Homer	NYYTGTTTACHN	1.00E-21	-4.98E+01	0	522	11.50%	3335.5	7.45%
FOXM1(Forkhead)/MCF7-FOXM1-ChIP-Seq(GSE72977)/Homer	TRTTTACTTW	1.00E-21	-4.90E+01	0	1040	22.91%	7751.5	17.32%
Pax8(Paired,Homeobox)/Thyroid-Pax8-ChIP-Seq(GSE26938)/Home	GTCATGCHTGRCTGS	1.00E-20	-4.78E+01	0	618	13.61%	4154.6	9.28%
Maz(Zf)/HepG2-Maz-ChIP-Seq(GSE31477)/Homer	GGGGGGGG	1.00E-20	-4.76E+01	0	1681	37.03%	13632	30.46%
Brn2(POU,Homeobox)/NPC-Brn2-ChIP-Seq(GSE35496)/Homer	ATGAATATTC	1.00E-19	-4.57E+01	0	165	3.63%	725.5	1.62%
BMAL1(bHLH)/Liver-Bmal1-ChIP-Seq(GSE39860)/Homer	GNCACGTG	1.00E-19	-4.45E+01	0	1959	43.15%	16367.2	36.57%
Sox9(HMG)/Limb-SOX9-ChIP-Seq(GSE73225)/Homer	AGGVNCCTTTGT	1.00E-19	-4.41E+01	0	1088	23.96%	8304.9	18.56%
Pitx1:Ebox(Homeobox,bHLH)/Hindlimb-Pitx1-ChIP-Seq(GSE41591)	YTAATTRAWWCCAGA	1.00E-18	-4.35E+01	0	287	6.32%	1601.3	3.58%
Rfx5(HTH)/GM12878-Rfx5-ChIP-Seq(GSE31477)/Homer	SCCTAGCAACAG	1.00E-18	-4.33E+01	0	445	9.80%	2826.3	6.31%
Foxa3(Forkhead)/Liver-Foxa3-ChIP-Seq(GSE77670)/Homer	BSNTGTTTACWYWGN	1.00E-18	-4.32E+01	0	395	8.70%	2431.2	5.43%
Brn1(POU,Homeobox)/NPC-Brn1-ChIP-Seq(GSE35496)/Homer	TATGCWAATBAV	1.00E-18	-4.27E+01	0	380	8.37%	2321.6	5.19%
p63(p53)/Keratinocyte-p63-ChIP-Seq(GSE17611)/Homer	NNDRCATGYCYNRRCA	1.00E-18	-4.18E+01	0	536	11.81%	3591.9	8.03%
ETS:E-box(ETS,bHLH)/HPC7-Scl-ChIP-Seq(GSE22178)/Homer	AGGAARCAGCTG	1.00E-17	-4.02E+01	0	207	4.56%	1059.8	2.37%
PAX5(Paired,Homeobox)/GM12878-PAX5-ChIP-Seq(GSE32465)/H	GCAGCCAAGCRTGACH	1.00E-17	-3.92E+01	0	582	12.82%	4025.6	8.99%
CRX(Homeobox)/Retina-Crx-ChIP-Seq(GSE20012)/Homer	GCTAATCC	1.00E-16	-3.85E+01	0	2126	46.83%	18188.7	40.64%
Tbet(T-box)/CD8-Tbet-ChIP-Seq(GSE33802)/Homer	AGGTGTGAAM	1.00E-16	-3.84E+01	0	1225	26.98%	9708.3	21.69%
BMYB(HTH)/Hela-BMYB-ChIP-Seq(GSE27030)/Homer	NHAACBGYYV	1.00E-16	-3.83E+01	0	1502	33.08%	12267.6	27.41%
NPAS(bHLH)/Liver-NPAS-ChIP-Seq(GSE39860)/Homer	NVCACGTG	1.00E-16	-3.70E+01	0	1733	38.17%	14481.5	32.36%
PAX6(Paired,Homeobox)/Forebrain-Pax6-ChIP-Seq(GSE66961)/Ho	NGTGTTCAVTSAAGCG	1.00E-16	-3.69E+01	0	166	3.66%	806.4	1.80%
CDX4(Homeobox)/ZebrafishEmbryos-Cdx4.Myc-ChIP-Seq(GSE4825	NGYCATAAAWCH	1.00E-15	-3.63E+01	0	880	19.38%	6669.2	14.90%
p73(p53)/Trachea-p73-ChIP-Seq(PRJNA310161)/Homer	NRRRCAWGTCCDGRC	1.00E-15	-3.61E+01	0	112	2.47%	459.9	1.03%
MYB(HTH)/ERMYB-Myb-ChIPSeq(GSE22095)/Homer	GGCVGTTR	1.00E-15	-3.60E+01	0	1718	37.84%	14377.9	32.13%
Cux2(Homeobox)/Liver-Cux2-ChIP-Seq(GSE35985)/Homer	HNRAATCAAT	1.00E-15	-3.59E+01	0	488	10.75%	3311.8	7.40%
Tbx20(T-box)/Heart-Tbx20-ChIP-Seq(GSE29636)/Homer	GGTGYTGACAGS	1.00E-15	-3.56E+01	0	358	7.89%	2261.4	5.05%
RFX(HTH)/K562-RFX3-ChIP-Seq(SRA012198)/Homer	CGGTTGCCATGGCAAC	1.00E-15	-3.54E+01	0	134	2.95%	605.3	1.35%
ZNF416(Zf)/HEK293-ZNF416.GFP-ChIP-Seq(GSE58341)/Homer	WDNCTGGGCA	1.00E-15	-3.51E+01	0	1917	42.22%	16307.5	36.44%
HOXA2(Homeobox)/mES-Hoxa2-ChIP-Seq(Donaldson_et_al.)/Hom	GYCATCMATCAT	1.00E-14	-3.40E+01	0	161	3.55%	799.2	1.79%
Rfx1(HTH)/NPC-H3K4me1-ChIP-Seq(GSE16256)/Homer	KGTTGCCATGGCAA	1.00E-14	-3.32E+01	0	302	6.65%	1858.1	4.15%
KLF14(Zf)/HEK293-KLF14.GFP-ChIP-Seq(GSE58341)/Homer	RGKGGGCGKGGC	1.00E-13	-3.21E+01	0	1950	42.95%	16742.8	37.41%
p53(p53)/Saos-p53-ChIP-Seq(GSE15780)/Homer	RRCATGYCYRGRCATG	1.00E-13	-3.17E+01	0	162	3.57%	829.2	1.85%
p53(p53)/Saos-p53-ChIP-Seq/Homer	RRCATGYCYRGRCATG	1.00E-13	-3.17E+01	0	162	3.57%	829.2	1.85%
X-box(HTH)/NPC-H3K4me1-ChIP-Seq(GSE16256)/Homer	GGTTGCCATGGCAA	1.00E-13	-3.16E+01	0	174	3.83%	917	2.05%
Sp5(Zf)/mES-Sp5.Flag-ChIP-Seq(GSE72989)/Homer	RGKGGGCGGAGC	1.00E-13	-3.15E+01	0	1188	26.17%	9598.3	21.45%
Oct4(POU,Homeobox)/mES-Oct4-ChIP-Seq(GSE11431)/Homer	ATTTGCATAW	1.00E-13	-3.15E+01	0	512	11.28%	3604.7	8.05%
HNF6(Homeobox)/Liver-Hnf6-ChIP-Seq(ERP000394)/Homer	NTATYGATCH	1.00E-13	-3.14E+01	0	565	12.44%	4056.5	9.06%
Rfx2(HTH)/LoVo-RFX2-ChIP-Seq(GSE49402)/Homer	GTTGCCATGGCAACM	1.00E-13	-3.13E+01	0	149	3.28%	743.1	1.66%
Mef2b(MADS)/HEK293-Mef2b.V5-ChIP-Seq(GSE67450)/Homer	GCTATTTTTGGM	1.00E-13	-3.00E+01	0	841	18.52%	6505.5	14.54%
Mef2c(MADS)/GM12878-Mef2c-ChIP-Seq(GSE32465)/Homer	DCYAAAAATAGM	1.00E-12	-2.88E+01	0	455	10.02%	3184.8	7.12%
Tbox:Smad(T-box,MAD)/ESCd5-Smad2_3-ChIP-Seq(GSE29422)/H	AGGTGHCAGACA	1.00E-12	-2.87E+01	0	304	6.70%	1946.2	4.35%
Otx2(Homeobox)/EpiLC-Otx2-ChIP-Seq(GSE56098)/Homer	NYTAATCCYB	1.00E-11	-2.69E+01	0	791	17.42%	6152	13.75%
ZNF467(Zf)/HEK293-ZNF467.GFP-ChIP-Seq(GSE58341)/Homer	TGGGGAAGGGCM	1.00E-11	-2.58E+01	0	1272	28.02%	10586.3	23.65%
GSC(Homeobox)/FrogEmbryos-GSC-ChIP-Seq(DRA000576)/Homer	RGGATTAR	1.00E-11	-2.57E+01	0	1073	23.63%	8748.4	19.55%
Cdx2(Homeobox)/mES-Cdx2-ChIP-Seq(GSE14586)/Homer	GYMATAAAAH	1.00E-11	-2.55E+01	0	672	14.80%	5141.2	11.49%
Hand2(bHLH)/Mesoderm-Hand2-ChIP-Seq(GSE61475)/Homer	TGACANARRCCAGRC	1.00E-10	-2.43E+01	0	697	15.35%	5397.2	12.06%
MafA(bZIP)/Islet-MafA-ChIP-Seq(GSE30298)/Homer	TGCTGACTCA	1.00E-10	-2.42E+01	0	1036	22.82%	8467.5	18.92%
Rbpj1(?)/Panc1-Rbpj1-ChIP-Seq(GSE47459)/Homer	HTTTCCCASG	1.00E-10	-2.39E+01	0	1748	38.50%	15163.1	33.88%
PAX3:FKHR-fusion(Paired,Homeobox)/Rh4-PAX3:FKHR-ChIP-Seq(G	ACCRTGACTAATTNN	1.00E-10	-2.32E+01	0	229	5.04%	1445.3	3.23%
Tcf4(HMG)/Hct116-Tcf4-ChIP-Seq(SRA012054)/Homer	ASATCAAAGGVA	1.00E-09	-2.22E+01	0	506	11.15%	3781.7	8.45%
HOXB13(Homeobox)/ProstateTumor-HOXB13-ChIP-Seq(GSE5628	TTTTATKRGG	1.00E-09	-2.10E+01	0	988	21.76%	8149.4	18.21%
HNF1b(Homeobox)/PDAC-HNF1B-ChIP-Seq(GSE64557)/Homer	GTTAATNATTAA	1.00E-08	-2.06E+01	0	166	3.66%	995	2.22%
OCT4-SOX2-TCF-NANOG(POU,Homeobox,HMG)/mES-Oct4-ChIP-S	ATTTGCATAACAATG	1.00E-08	-2.04E+01	0	213	4.69%	1365.9	3.05%
Foxh1(Forkhead)/hESC-FOXH1-ChIP-Seq(GSE29422)/Homer	NNTGTGGATTSS	1.00E-08	-1.97E+01	0	642	14.14%	5058.8	11.30%
Mef2d(MADS)/Retina-Mef2d-ChIP-Seq(GSE61391)/Homer	GCTATTTTTAGC	1.00E-08	-1.96E+01	0	215	4.74%	1395.2	3.12%
Pbx3(Homeobox)/GM12878-PBX3-ChIP-Seq(GSE32465)/Homer	SCTGTCAMTCAN	1.00E-08	-1.92E+01	0	334	7.36%	2386.4	5.33%
NFAT(RHD)/Jurkat-NFATC1-ChIP-Seq(Jolma_et_al.)/Homer	ATTTTCCATT	1.00E-08	-1.90E+01	0	969	21.34%	8056.8	18.00%
PBX1(Homeobox)/MCF7-PBX1-ChIP-Seq(GSE28007)/Homer	GSCTGTCACTCA	1.00E-08	-1.86E+01	0	136	3.00%	794.8	1.78%
Brachyury(T-box)/Mesoendoderm-Brachyury-ChIP-exo(GSE54963)	ANTTMRCASBNNNGT	1.00E-07	-1.81E+01	0	323	7.11%	2318.3	5.18%
Pax7(Paired,Homeobox)/Myoblast-Pax7-ChIP-Seq(GSE25064)/Hor	TAATCAATTA	1.00E-07	-1.80E+01	0	120	2.64%	683.7	1.53%
ZNF415(Zf)/HEK293-ZNF415.GFP-ChIP-Seq(GSE58341)/Homer	GRTGMTRGAGCC	1.00E-07	-1.75E+01	0	795	17.51%	6523.5	14.58%
ZNF189(Zf)/HEK293-ZNF189.GFP-ChIP-Seq(GSE58341)/Homer	TGGAACAGMA	1.00E-07	-1.75E+01	0	1010	22.25%	8504	19.00%
HIF-1b(HLH)/T47D-HIF1b-ChIP-Seq(GSE59937)/Homer	RTACGTGC	1.00E-07	-1.74E+01	0	1053	23.19%	8907.3	19.90%
TATA-Box(TBP)/Promoter/Homer	CCTTTTAWAGSC	1.00E-07	-1.70E+01	0	1228	27.05%	10566.3	23.61%
GATA3(Zf)/iTreg-Gata3-ChIP-Seq(GSE20898)/Homer	AGATAASR	1.00E-07	-1.67E+01	0	1328	29.25%	11527.6	25.76%
Arnt:Ahr(bHLH)/MCF7-Arnt-ChIP-Seq(Lo_et_al.)/Homer	TBGCACGCAA	1.00E-07	-1.65E+01	0	662	14.58%	5352.9	11.96%
PR(NR)/T47D-PR-ChIP-Seq(GSE31130)/Homer	VAGRACAKNCTGTBC	1.00E-07	-1.65E+01	0	2150	47.36%	19449.9	43.46%

Supplemental table 3. Characteristics of the ASCL1 and NEUROD1 ChIP-seq datasets

Sample	ChIP-seq	Uniquely Mapped Reads (M)	Total_Peaks	DHS (per 5000 peaks)	FRiP
LuCaP49	ASCL1	26.1	46859	4732	32.5
LuCaP93	ASCL1	25.9	49994	4664	21.1
LuCaP145_1	ASCL1	38.8	46303	4259	38.9
H660	ASCL1	25	61338	4869	34.4
LuCaP173_p1	NEUROD1	31.9	78124	4857	35.1
LuCaP49	H3K27ac	94.7	27699	4690	9.2
LuCaP93	H3K27ac	96.9	68618	4729	43.9
LuCaP145_1	H3K27ac	74.3	69086	4665	48
LuCaP145_2	H3K27ac	98.6	57843	4968	54.2
LuCaP173_p1	H3K27ac	93.2	65495	4945	20.2
EF-1	H3K27ac	101.2	68984	4969	44.8

DHS: DNase I hypersensitive site (per 5000 peaks)
FRIP: fraction of reads that fall into a peak
