# Supplementary material for: Subtype heterogeneity and epigenetic convergence in neuroendocrine prostate cancer
Source: Nat Commun. 2021 Oct 1;12:5775. doi: 10.1038/s41467-021-26042-z (PMC8486778; doi:10.1038/s41467-021-26042-z)
Supplement: Supplementary file 2 — Description of Additional Supplementary Files [file 41467_2021_26042_MOESM2_ESM.pdf]

Description of Additional Supplementary Files

File Name: Supplementary Data 1
Description: Motif analysis of ASCL1, NEUROD1 and shared peaks.

File Name: Supplementary Data 2
Description: Genes marked with superenhancers in NEPC models.
