# Supplementary material for: Subtype heterogeneity and epigenetic convergence in neuroendocrine prostate cancer
Source: Nat Commun. 2021 Oct 1;12:5775. doi: 10.1038/s41467-021-26042-z (PMC8486778; doi:10.1038/s41467-021-26042-z)
Supplement: Supplementary file 3 — Supplementary Data 1 [file 41467_2021_26042_MOESM3_ESM.pdf]

Supplemental Table 4. Motif analysis

Cluster	Name	PValue	log(PValue)	# Target Seq	% of Targets	# Backgroun	% of Background Sequences with Motif
1	E2A(bHLH)/proBcell-E2A-ChIP-Seq(GSE21978)/Homer	1.00E-110	-255.22705	1685	58.79%	17586.2	38.05%
	HEB(bHLH)/mES-Heb-ChIP-Seq(GSE53233)/Homer	1.00E-90	-209.01491	1857	64.79%	21239.3	45.96%
	Ascl1(bHLH)/NeuralTubes-Ascl1-ChIP-Seq(GSE55840)/Homer	1.00E-86	-199.9032	1634	57.01%	17875.1	38.68%
	Tcf12(bHLH)/GM12878-Tcf12-ChIP-Seq(GSE32465)/Homer	1.00E-68	-157.55262	1152	40.20%	11653.1	25.21%
	Ptf1a(bHLH)/Panc1-Ptf1a-ChIP-Seq(GSE47459)/Homer	1.00E-67	-156.4127	2031	70.87%	25387.8	54.93%
	MyoG(bHLH)/C2C12-MyoG-ChIP-Seq(GSE36024)/Homer	1.00E-64	-148.82983	1139	39.74%	11650.9	25.21%
	Myf5(bHLH)/GM-Myf5-ChIP-Seq(GSE24852)/Homer	1.00E-58	-134.47143	804	28.05%	7413.4	16.04%
	MyoD(bHLH)/Myotube-MyoD-ChIP-Seq(GSE21614)/Homer	1.00E-50	-115.57859	889	31.02%	8907.5	19.27%
	Ap4(bHLH)/AML-Tfap4-ChIP-Seq(GSE45738)/Homer	1.00E-44	-102.41205	1119	39.04%	12443.3	26.92%
	Tcf21(bHLH)/ArterySmoothMuscle-Tcf21-ChIP-Seq(GSE61369)/Hon	1.00E-42	-97.549444	956	33.36%	10244.9	22.17%
	Atoh1(bHLH)/Cerebellum-Atoh1-ChIP-Seq(GSE22111)/Homer	1.00E-41	-94.715422	1002	34.96%	10965.4	23.73%
	NeuroD1(bHLH)/Islet-NeuroD1-ChIP-Seq(GSE30298)/Homer	1.00E-31	-71.472692	752	26.24%	8077.4	17.48%
	HLH-1(bHLH)/cElegans-Embryo-HLH1-ChIP-Seq(modEncode)/Homer	1.00E-31	-73.398524	591	20.62%	5866.7	12.69%
	NeuroG2(bHLH)/Fibroblast-NeuroG2-ChIP-Seq(GSE75910)/Homer	1.00E-28	-64.647191	1096	38.24%	13222.3	28.61%
	TCF4(bHLH)/SHSY5Y-TCF4-ChIP-Seq(GSE96915)/Homer	1.00E-24	-56.602185	1110	38.73%	13716.6	29.68%
	Olig2(bHLH)/Neuron-Olig2-ChIP-Seq(GSE30882)/Homer	1.00E-22	-52.903628	1138	39.71%	14278.9	30.90%
	bZIP52(bZIP)/colamp-bZIP52-DAP-Seq(GSE60143)/Homer	1.00E-13	-30.147818	729	25.44%	9130.3	19.76%
	SCL(bHLH)/HPC7-Scl-ChIP-Seq(GSE13511)/Homer	1.00E-09	-21.814502	2217	77.36%	33404.7	72.28%
	E-box/Drosophila-Promoters/Homer	1.00E-06	-14.184119	166	5.79%	1808.7	3.91%
	VIP1(bZIP)/col-VIP1-DAP-Seq(GSE60143)/Homer	1.00E-05	-13.747023	138	4.82%	1452.1	3.14%
	bZIP69(bZIP)/col-bZIP69-DAP-Seq(GSE60143)/Homer	1.00E-04	-9.562546	91	3.18%	958.5	2.07%
2	Slug(Zf)/Mesoderm-Snai2-ChIP-Seq(GSE61475)/Homer	1.00E-102	-235.50077	955	33.32%	7752.4	16.77%
	E2A(bHLH),near_PU.1/Bcell-PU.1-ChIP-Seq(GSE21512)/Homer	1.00E-95	-220.75889	1574	54.92%	16537.2	35.78%
	ZEB1(Zf)/PDAC-ZEB1-ChIP-Seq(GSE64557)/Homer	1.00E-72	-167.48717	1565	54.61%	17512.4	37.89%
	ZEB2(Zf)/SNU398-ZEB2-ChIP-Seq(GSE103048)/Homer	1.00E-57	-133.09837	1089	38.00%	11284.4	24.42%
	NGA4(ABI3VP1)/col-NGA4-DAP-Seq(GSE60143)/Homer	1.00E-44	-103.50082	1752	61.13%	22160.5	47.95%
3	NF1(CTF)/LNCAP-NF1-ChIP-Seq(Unpublished)/Homer	1.00E-38	-88.691844	502	17.52%	4433.6	9.59%
	Tlx?(NR)/NPC-H3K4me1-ChIP-Seq(GSE16256)/Homer	1.00E-22	-52.253879	456	15.91%	4593.6	9.94%
4	Nkx2.2(Homeobox)/NPC-Nkx2.2-ChIP-Seq(GSE61673)/Homer	1.00E-22	-51.855894	1065	37.16%	13219.4	28.60%
	Nkx2.1(Homeobox)/LungAC-Nkx2.1-ChIP-Seq(GSE43252)/Homer	1.00E-17	-39.845184	1334	46.55%	17862	38.65%
	Nkx2.5(Homeobox)/HL1-Nkx2.5.biotin-ChIP-Seq(GSE21529)/Homer	1.00E-15	-35.173776	1067	37.23%	13958.3	30.20%
	Bapx1(Homeobox)/VertebralCol-Bapx1-ChIP-Seq(GSE36672)/Home	1.00E-14	-32.308535	988	34.47%	12895.5	27.90%
	Nkx3.1(Homeobox)/LNCaP-Nkx3.1-ChIP-Seq(GSE28264)/Homer	1.00E-08	-18.494397	963	33.60%	13290.5	28.76%
5	FOXA1(Forkhead)/MCF7-FOXA1-ChIP-Seq(GSE26831)/Homer	1.00E-19	-44.758669	316	11.03%	2975.7	6.44%
	FOXA1(Forkhead)/LNCAP-FOXA1-ChIP-Seq(GSE27824)/Homer	1.00E-16	-38.344563	372	12.98%	3842.5	8.31%
	FOXM1(Forkhead)/MCF7-FOXM1-ChIP-Seq(GSE72977)/Homer	1.00E-15	-35.893583	327	11.41%	3318.8	7.18%
	Foxa3(Forkhead)/Liver-Foxa3-ChIP-Seq(GSE77670)/Homer	1.00E-11	-27.360845	125	4.36%	1010.2	2.19%
	Foxa2(Forkhead)/Liver-Foxa2-ChIP-Seq(GSE25694)/Homer	1.00E-11	-26.238184	284	9.91%	3013.1	6.52%
	FOXP1(Forkhead)/H9-FOXP1-ChIP-Seq(GSE31006)/Homer	1.00E-11	-26.384587	156	5.44%	1388.1	3.00%
	Foxf1(Forkhead)/Lung-Foxf1-ChIP-Seq(GSE77951)/Homer	1.00E-11	-26.376856	256	8.93%	2642.9	5.72%
	FOXK1(Forkhead)/HEK293-FOXK1-ChIP-Seq(GSE51673)/Homer	1.00E-10	-25.205995	326	11.37%	3606.7	7.80%
	FoxL2(Forkhead)/Ovary-FoxL2-ChIP-Seq(GSE60858)/Homer	1.00E-10	-24.575174	236	8.23%	2430	5.26%
	Fox:Ebox(Forkhead,bHLH)/Panc1-Foxa2-ChIP-Seq(GSE47459)/Home	1.00E-09	-22.974199	437	15.25%	5224.3	11.30%
	Foxo3(Forkhead)/U2OS-Foxo3-ChIP-Seq(E-MTAB-2701)/Homer	1.00E-09	-22.596753	236	8.23%	2484.3	5.38%
	Foxo1(Forkhead)/RAW-Foxo1-ChIP-Seq(Fan_et_al.)/Homer	1.00E-07	-16.218182	769	26.83%	10470.1	22.66%
	FOXK2(Forkhead)/U2OS-FOXK2-ChIP-Seq(E-MTAB-2204)/Homer	1.00E-06	-14.245711	222	7.75%	2564.4	5.55%
	PHA-4(Forkhead)/cElegans-Embryos-PHA4-ChIP-Seq(modEncode)/H	1.00E-03	-8.322908	989	34.51%	14531.4	31.44%
6	NF1-halfsite(CTF)/LNCaP-NF1-ChIP-Seq(Unpublished)/Homer	1.00E-18	-43.677795	1234	43.06%	16129.2	34.90%
	HIC1(Zf)/Treg-ZBTB29-ChIP-Seq(GSE99889)/Homer	1.00E-10	-25.13493	1474	51.43%	20884.9	45.19%
7	bHLHE41(bHLH)/proB-Bhlhe41-ChIP-Seq(GSE93764)/Homer	1.00E-18	-41.630877	793	27.67%	9595.2	20.76%
	IBL1(bHLH)/Seedling-IBL1-ChIP-Seq(GSE51120)/Homer	1.00E-16	-38.587055	1186	41.38%	15621.4	33.80%
	ABF1(bZIP)/Arabidopsis-ABF1-ChIP-Seq(GSE80564)/Homer	1.00E-13	-31.472567	645	22.51%	7851	16.99%
	HIF-1b(HLH)/T47D-HIF1b-ChIP-Seq(GSE59937)/Homer	1.00E-06	-15.027077	617	21.53%	8251.7	17.85%
	Pho2(bHLH)/Yeast-Pho2-ChIP-Seq(GSE29506)/Homer	1.00E-06	-15.073435	324	11.30%	3957.6	8.56%
	ABI5(bZIP)/col-ABI5-DAP-Seq(GSE60143)/Homer	1.00E-06	-15.191226	271	9.46%	3206.8	6.94%
	c-Myc(bHLH)/LNCAP-cMyc-ChIP-Seq(Unpublished)/Homer	1.00E-05	-12.813893	341	11.90%	4308.7	9.32%
	SPCH(bHLH)/Seedling-SPCH-ChIP-Seq(GSE57497)/Homer	1.00E-05	-12.384714	538	18.77%	7236.6	15.66%
	E-box/Arabidopsis-Promoters/Homer	1.00E-04	-9.323711	297	10.36%	3859	8.35%
	HIF2a(bHLH)/785_O-HIF2a-ChIP-Seq(GSE34871)/Homer	1.00E-03	-8.474795	209	7.29%	2630.8	5.69%
	HIF-1a(bHLH)/MCF7-HIF1a-ChIP-Seq(GSE28352)/Homer	1.00E-03	-8.500007	168	5.86%	2047	4.43%
	Pho4(bHLH)/Yeast-Pho4-ChIP-Seq(GSE29506)/Homer	1.00E-03	-8.308655	171	5.97%	2097.6	4.54%
	At4g18890(BZR)/col-At4g18890-DAP-Seq(GSE60143)/Homer	1.00E-03	-7.784062	131	4.57%	1560	3.38%
	PIF7(bHLH)/col-PIF7-DAP-Seq(GSE60143)/Homer	1.00E-03	-8.167853	156	5.44%	1892.2	4.09%
	CLOCK(bHLH)/Liver-Clock-ChIP-Seq(GSE39860)/Homer	1.00E-03	-8.03244	328	11.44%	4395.5	9.51%
	BMAL1(bHLH)/Liver-Bmal1-ChIP-Seq(GSE39860)/Homer	1.00E-03	-7.670583	875	30.53%	12811.4	27.72%
	BIM2(bHLH)/col-BIM2-DAP-Seq(GSE60143)/Homer	1.00E-03	-7.04056	419	14.62%	5834.6	12.62%
	PIF5ox(bHLH)/Arabidopsis-PIF5ox-ChIP-Seq(GSE35062)/Homer	1.00E-03	-8.30089	561	19.57%	7888.7	17.07%
	At4g36780(BZR)/col-At4g36780-DAP-Seq(GSE60143)/Homer	1.00E-02	-6.279845	188	6.56%	2446.9	5.29%
	At1g78700(BZR)/col-At1g78700-DAP-Seq(GSE60143)/Homer	1.00E-02	-6.098334	195	6.80%	2559.5	5.54%
	bHLH34(bHLH)/colamp-bHLH34-DAP-Seq(GSE60143)/Homer	1.00E-02	-6.370904	177	6.18%	2281.8	4.94%
	n-Myc(bHLH)/mES-nMyc-ChIP-Seq(GSE11431)/Homer	1.00E-02	-6.019482	464	16.19%	6610.4	14.30%
	c-Myc(bHLH)/mES-cMyc-ChIP-Seq(GSE11431)/Homer	1.00E-02	-5.328068	352	12.28%	4965.5	10.74%
	NPAS(bHLH)/Liver-NPAS-ChIP-Seq(GSE39860)/Homer	1.00E-02	-4.668028	751	26.20%	11227	24.29%
	USF1(bHLH)/GM12878-Usf1-ChIP-Seq(GSE32465)/Homer	1.00E-02	-6.527372	284	9.91%	3845.1	8.32%
	PIF4(bHLH)/Seedling-PIF4-ChIP-Seq(GSE35315)/Homer	1.00E-02	-5.593947	632	22.05%	9249.8	20.01%
8	Twist(bHLH)/HMLE-TWIST1-ChIP-Seq(Chang_et_al)/Homer	1.00E-16	-38.436889	182	6.35%	1485	3.21%
9	PRDM9(Zf)/Testis-DMC1-ChIP-Seq(GSE35498)/Homer	1.00E-12	-28.905794	353	12.32%	3855.2	8.34%
10	Tbx5(T-box)/HL1-Tbx5.biotin-ChIP-Seq(GSE21529)/Homer	1.00E-12	-29.742108	1654	57.71%	23510.6	50.87%
	Tbx21(T-box)/GM12878-TBX21-ChIP-Seq(Encode)/Homer	1.00E-10	-24.215784	448	15.63%	5330.7	11.53%
	Tbet(T-box)/CD8-Tbet-ChIP-Seq(GSE33802)/Homer	1.00E-08	-19.911369	459	16.02%	5668.8	12.27%
	Tbx6(T-box)/ESC-Tbx6-ChIP-Seq(GSE93524)/Homer	1.00E-07	-16.335892	564	19.68%	7383.4	15.98%
	Eomes(T-box)/H9-Eomes-ChIP-Seq(GSE26097)/Homer	1.00E-07	-18.243601	822	28.68%	11146.4	24.12%
	Tbr1(T-box)/Cortex-Tbr1-ChIP-Seq(GSE71384)/Homer	1.00E-02	-6.563408	487	16.99%	6911.3	14.95%
11	Zic3(Zf)/mES-Zic3-ChIP-Seq(GSE37889)/Homer	1.00E-11	-25.404436	685	23.90%	8701.2	18.83%
	Unknown-ESC-element(?)/mES-Nanog-ChIP-Seq(GSE11724)/Home	1.00E-09	-22.074411	581	20.27%	7335.9	15.87%

	Zic(Zf)/Cerebellum-ZIC1.2-ChIP-Seq(GSE60731)/Homer	1.00E-05	-11.599255	633	22.09%	8724.6	18.88%
12	bHLH130(bHLH)/col-bHLH130-DAP-Seq(GSE60143)/Homer	1.00E-10	-23.477857	333	11.62%	3758.7	8.13%
	bHLH122(bHLH)/col100-bHLH122-DAP-Seq(GSE60143)/Homer	1.00E-08	-19.055288	366	12.77%	4381.5	9.48%
	bHLH80(bHLH)/col-bHLH80-DAP-Seq(GSE60143)/Homer	1.00E-05	-12.234659	417	14.55%	5448.2	11.79%
13	NPAS2(bHLH)/Liver-NPAS2-ChIP-Seq(GSE39860)/Homer	1.00E-10	-24.589843	714	24.91%	9169.8	19.84%
	MITF(bHLH)/MastCells-MITF-ChIP-Seq(GSE48085)/Homer	1.00E-02	-5.95607	430	15.00%	6096.7	13.19%
	Usf2(bHLH)/C2C12-Usf2-ChIP-Seq(GSE36030)/Homer	1.00E-02	-5.138752	159	5.55%	2091.9	4.53%
14	Six2(Homeobox)/NephronProgenitor-Six2-ChIP-Seq(GSE39837)/Hor	1.00E-08	-19.450308	333	11.62%	3903	8.45%
	Six1(Homeobox)/Myoblast-Six1-ChIP-Chip(GSE20150)/Homer	1.00E-06	-15.547844	97	3.38%	891.4	1.93%
	Six4(Homeobox)/MCF7-SIX4-ChIP-Seq(Encode)/Homer	1.00E-03	-7.854204	28	0.98%	219.3	0.47%
15	ATY13(MYB)/col-ATY13-DAP-Seq(GSE60143)/Homer	1.00E-08	-20.140357	1272	44.38%	18001	38.95%
	MYB(HTH)/ERMYB-Myb-ChIPSeq(GSE22095)/Homer	1.00E-04	-9.288152	696	24.28%	9873.6	21.36%
	MYB77(MYB)/col-MYB77-DAP-Seq(GSE60143)/Homer	1.00E-03	-7.758513	470	16.40%	6548.6	14.17%
	AMYB(HTH)/Testes-AMYB-ChIP-Seq(GSE44588)/Homer	1.00E-03	-7.670032	601	20.97%	8557.4	18.52%
	BMYB(HTH)/Hela-BMYB-ChIP-Seq(GSE27030)/Homer	1.00E-02	-5.702459	531	18.53%	7672.6	16.60%
16	ZBTB18(Zf)/HEK293-ZBTB18.GFP-ChIP-Seq(GSE58341)/Homer	1.00E-08	-18.622602	367	12.81%	4413.9	9.55%
	Tal1	1.00E-05	-13.006529	748	26.10%	10373.9	22.45%
17	ETV4(ETS)/HepG2-ETV4-ChIP-Seq(ENCODE)/Homer	1.00E-08	-20.416359	681	23.76%	8895.1	19.25%
	GABPA(ETS)/Jurkat-GABPa-ChIP-Seq(GSE17954)/Homer	1.00E-07	-16.890655	491	17.13%	6278.5	13.59%
	EWS:ERG-fusion(ETS)/CADO_ES1-EWS:ERG-ChIP-Seq(SRA014231)/	1.00E-07	-18.02438	329	11.48%	3903.6	8.45%
	Elk1(ETS)/Hela-Elk1-ChIP-Seq(GSE31477)/Homer	1.00E-06	-14.173838	328	11.44%	4055.8	8.78%
	Fli1(ETS)/CD8-FLI-ChIP-Seq(GSE20898)/Homer	1.00E-06	-16.030952	599	20.90%	7921.6	17.14%
	Elf4(ETS)/BMDM-Elf4-ChIP-Seq(GSE88699)/Homer	1.00E-06	-14.924759	520	18.14%	6812.4	14.74%
	EHF(ETS)/LoVo-EHF-ChIP-Seq(GSE49402)/Homer	1.00E-06	-15.510104	600	20.94%	7967.8	17.24%
	ELF1(ETS)/Jurkat-ELF1-ChIP-Seq(SRA014231)/Homer	1.00E-05	-12.426327	310	10.82%	3881.9	8.40%
	ETV1(ETS)/GIST48-ETV1-ChIP-Seq(GSE22441)/Homer	1.00E-05	-13.205242	712	24.84%	9810	21.23%
	EWS:FLI1-fusion(ETS)/SK_N_MC-EWS:FLI1-ChIP-Seq(SRA014231)/I	1.00E-05	-13.135124	311	10.85%	3861.9	8.36%
	SPDEF(ETS)/VCaP-SPDEF-ChIP-Seq(SRA014231)/Homer	1.00E-05	-12.899109	532	18.56%	7113	15.39%
	ELF5(ETS)/T47D-ELF5-ChIP-Seq(GSE30407)/Homer	1.00E-05	-13.090896	341	11.90%	4294.2	9.29%
	ELF3(ETS)/PDAC-ELF3-ChIP-Seq(GSE64557)/Homer	1.00E-05	-12.602727	337	11.76%	4261.5	9.22%
	Elk4(ETS)/Hela-Elk4-ChIP-Seq(GSE31477)/Homer	1.00E-03	-9.125533	307	10.71%	4016.5	8.69%
	ETS(ETS)/Promoter/Homer	1.00E-03	-8.137307	177	6.18%	2190	4.74%
	Ets1-distal(ETS)/CD4+-PolII-ChIP-Seq(Barski_et_al.)/Homer	1.00E-03	-8.11849	149	5.20%	1796.9	3.89%
18	RBPJ:Ebox(?,bHLH)/Panc1-Rbpj1-ChIP-Seq(GSE47459)/Homer	1.00E-07	-17.503461	274	9.56%	3159.3	6.84%
19	LBD23(LOBAS2)/colamp-LBD23-DAP-Seq(GSE60143)/Homer	1.00E-07	-16.124616	422	14.72%	5313.1	11.50%
20	AP-2alpha(AP2)/Hela-AP2alpha-ChIP-Seq(GSE31477)/Homer	1.00E-07	-18.150437	827	28.86%	11228.1	24.30%
	AP-2gamma(AP2)/MCF7-TFAP2C-ChIP-Seq(GSE21234)/Homer	1.00E-05	-11.984025	975	34.02%	13964.7	30.22%
21	THRb(NR)/HepG2-THRb.Flag-ChIP-Seq(Encode)/Homer	1.00E-07	-18.418654	502	17.52%	6361.9	13.77%
	THRa(NR)/C17.2-THRa-ChIP-Seq(GSE38347)/Homer	1.00E-03	-9.019915	305	10.64%	3993.5	8.64%
22	ERF38(AP2EREBP)/col-ERF38-DAP-Seq(GSE60143)/Homer	1.00E-07	-16.920344	210	7.33%	2309.7	5.00%
	DREB19(AP2EREBP)/colamp-DREB19-DAP-Seq(GSE60143)/Homer	1.00E-06	-15.331605	221	7.71%	2511.6	5.43%
	AT3G16280(AP2EREBP)/colamp-AT3G16280-DAP-Seq(GSE60143)/	1.00E-06	-14.284725	143	4.99%	1502.2	3.25%
	DEAR3(AP2EREBP)/colamp-DEAR3-DAP-Seq(GSE60143)/Homer	1.00E-06	-15.116694	143	4.99%	1479.4	3.20%
	At5g65130(AP2EREBP)/colamp-At5g65130-DAP-Seq(GSE60143)/H	1.00E-05	-11.985054	127	4.43%	1359.3	2.94%
	At1g36060(AP2EREBP)/colamp-At1g36060-DAP-Seq(GSE60143)/H	1.00E-05	-13.606449	282	9.84%	3428	7.42%
	DEAR5(AP2EREBP)/col-DEAR5-DAP-Seq(GSE60143)/Homer	1.00E-05	-12.575618	81	2.83%	758.1	1.64%
	DEAR2(AP2EREBP)/colamp-DEAR2-DAP-Seq(GSE60143)/Homer	1.00E-05	-12.987822	426	14.86%	5537.5	11.98%
	CEJ1(AP2EREBP)/col-CEJ1-DAP-Seq(GSE60143)/Homer	1.00E-05	-12.19864	392	13.68%	5084	11.00%
	At1g19210(AP2EREBP)/colamp-At1g19210-DAP-Seq(GSE60143)/H	1.00E-04	-10.616848	569	19.85%	7826.3	16.93%
	bHLH10(bHLH)/colamp-bHLH10-DAP-Seq(GSE60143)/Homer	1.00E-04	-9.323019	119	4.15%	1337.7	2.89%
	TINY(AP2EREBP)/col-TINY-DAP-Seq(GSE60143)/Homer	1.00E-04	-9.82057	109	3.80%	1187.1	2.57%
	At2g44940(AP2EREBP)/colamp-At2g44940-DAP-Seq(GSE60143)/H	1.00E-04	-10.785113	91	3.18%	925.1	2.00%
	AT1G01250(AP2EREBP)/col-AT1G01250-DAP-Seq(GSE60143)/Hom	1.00E-04	-11.116201	50	1.74%	417.8	0.90%
	AT1G71450(AP2EREBP)/col-AT1G71450-DAP-Seq(GSE60143)/Hom	1.00E-04	-10.59095	644	22.47%	8968.5	19.41%
	At4g31060(AP2EREBP)/colamp-At4g31060-DAP-Seq(GSE60143)/H	1.00E-04	-11.334477	158	5.51%	1795.4	3.88%
	At4g28140(AP2EREBP)/colamp-At4g28140-DAP-Seq(GSE60143)/H	1.00E-03	-6.981325	146	5.09%	1805.3	3.91%
	DREB2(AP2EREBP)/col-DREB2-DAP-Seq(GSE60143)/Homer	1.00E-03	-8.453546	167	5.83%	2034.9	4.40%
	At4g32800(AP2EREBP)/colamp-At4g32800-DAP-Seq(GSE60143)/H	1.00E-03	-8.89996	48	1.67%	433.9	0.94%
	RAP21(AP2EREBP)/colamp-RAP21-DAP-Seq(GSE60143)/Homer	1.00E-03	-9.160037	77	2.69%	788.5	1.71%
	At1g22810(AP2EREBP)/colamp-At1g22810-DAP-Seq(GSE60143)/H	1.00E-03	-7.700125	180	6.28%	2254.7	4.88%
	At1g77640(AP2EREBP)/col-At1g77640-DAP-Seq(GSE60143)/Homer	1.00E-03	-8.347334	106	3.70%	1195.9	2.59%
	DREB26(AP2EREBP)/col-DREB26-DAP-Seq(GSE60143)/Homer	1.00E-03	-8.079812	131	4.57%	1547.8	3.35%
	At1g75490(AP2EREBP)/colamp-At1g75490-DAP-Seq(GSE60143)/H	1.00E-02	-4.730595	703	24.53%	10464.4	22.64%
	AT1G44830(AP2EREBP)/col-AT1G44830-DAP-Seq(GSE60143)/Hom	1.00E-02	-5.099287	163	5.69%	2152.9	4.66%
23	Fra2(bZIP)/Striatum-Fra2-ChIP-Seq(GSE43429)/Homer	1.00E-07	-17.324481	200	6.98%	2163.1	4.68%
	Fosl2(bZIP)/3T3L1-Fosl2-ChIP-Seq(GSE56872)/Homer	1.00E-07	-16.945577	155	5.41%	1585.2	3.43%
	Jun-AP1(bZIP)/K562-cJun-ChIP-Seq(GSE31477)/Homer	1.00E-07	-16.926172	119	4.15%	1131.1	2.45%
	Atf3(bZIP)/GBM-ATF3-ChIP-Seq(GSE33912)/Homer	1.00E-07	-16.418993	246	8.58%	2811.4	6.08%
	Fra1(bZIP)/BT549-Fra1-ChIP-Seq(GSE46166)/Homer	1.00E-07	-16.777256	211	7.36%	2327.8	5.04%
	AP-1(bZIP)/ThioMac-PU.1-ChIP-Seq(GSE21512)/Homer	1.00E-06	-16.02629	283	9.87%	3340.2	7.23%
	JunB(bZIP)/DendriticCells-Junb-ChIP-Seq(GSE36099)/Homer	1.00E-06	-16.104021	215	7.50%	2403.5	5.20%
	BATF(bZIP)/Th17-BATF-ChIP-Seq(GSE39756)/Homer	1.00E-06	-15.863378	239	8.34%	2738.5	5.93%
	Bach2(bZIP)/OCILy7-Bach2-ChIP-Seq(GSE44420)/Homer	1.00E-03	-8.221192	99	3.45%	1105.6	2.39%
	NFE2L2(bZIP)/HepG2-NFE2L2-ChIP-Seq(Encode)/Homer	1.00E-02	-4.765266	21	0.73%	189	0.41%
	NF-E2(bZIP)/K562-NFE2-ChIP-Seq(GSE31477)/Homer	1.00E-02	-4.699396	32	1.12%	326.2	0.71%
24	At5g05790(MYBrelated)/col-At5g05790-DAP-Seq(GSE60143)/Hom	1.00E-07	-17.814649	269	9.39%	3079.8	6.66%
	At3g11280(MYBrelated)/col-At3g11280-DAP-Seq(GSE60143)/Hom	1.00E-07	-17.771248	258	9.00%	2930.6	6.34%
	Gata4(Zf)/Heart-Gata4-ChIP-Seq(GSE35151)/Homer	1.00E-06	-14.895189	299	10.43%	3611.9	7.82%
	GATA3(Zf)/iTreg-Gata3-ChIP-Seq(GSE20898)/Homer	1.00E-05	-11.54036	402	14.03%	5268.5	11.40%
	Gata6(Zf)/HUG1N-GATA6-ChIP-Seq(GSE51936)/Homer	1.00E-05	-12.100115	260	9.07%	3183.7	6.89%
	Gata1(Zf)/K562-GATA1-ChIP-Seq(GSE18829)/Homer	1.00E-04	-10.885448	183	6.39%	2155.9	4.66%
	Gata2(Zf)/K562-GATA2-ChIP-Seq(GSE18829)/Homer	1.00E-04	-10.755419	200	6.98%	2398	5.19%
	At5g58900(MYBrelated)/colamp-At5g58900-DAP-Seq(GSE60143)/I	1.00E-03	-8.780241	310	10.82%	4081.6	8.83%
	At5g08520(MYBrelated)/colamp-At5g08520-DAP-Seq(GSE60143)/I	1.00E-03	-7.637353	297	10.36%	3962.3	8.57%
	ELT-3(Gata)/cElegans-L1-ELT3-ChIP-Seq(modEncode)/Homer	1.00E-03	-8.974191	106	3.70%	1174.8	2.54%
	PQM-1(?)/cElegans-L3-ChIP-Seq(modEncode)/Homer	1.00E-03	-8.311567	99	3.45%	1102.4	2.39%
	At1g49010(MYBrelated)/col-At1g49010-DAP-Seq(GSE60143)/Hom	1.00E-02	-6.778489	471	16.43%	6647.9	14.38%

25	PABPC1(?)/MEL-PABC1-CLIP-Seq(GSE69755)/Homer	1.00E-07	-17.298293	421	14.69%	5242.4	11.34%
	Hoxa11(Homeobox)/ChickenMSG-Hoxa11.Flag-ChIP-Seq(GSE86088	1.00E-05	-12.301896	609	21.25%	8310.3	17.98%
	HOXD13(Homeobox)/Chicken-Hoxd13-ChIP-Seq(GSE38910)/Homer	1.00E-05	-12.589196	249	8.69%	3006.7	6.51%
	caudal(Homeobox)/Drosophila-Embryos-ChIP-Chip(modEncode)/Ho	1.00E-04	-9.51921	197	6.87%	2409.5	5.21%
	CDX4(Homeobox)/ZebrafishEmbryos-Cdx4.Myc-ChIP-Seq(GSE48254	1.00E-04	-9.38867	217	7.57%	2699.5	5.84%
	Hoxa13(Homeobox)/ChickenMSG-Hoxa13.Flag-ChIP-Seq(GSE86088	1.00E-04	-9.85432	623	21.74%	8706.7	18.84%
	Hoxd13(Homeobox)/ChickenMSG-Hoxd13.Flag-ChIP-Seq(GSE86088	1.00E-04	-11.116081	392	13.68%	5146.8	11.14%
	Cdx2(Homeobox)/mES-Cdx2-ChIP-Seq(GSE14586)/Homer	1.00E-03	-8.536871	155	5.41%	1862.9	4.03%
	Hoxd11(Homeobox)/ChickenMSG-Hoxd11.Flag-ChIP-Seq(GSE86088	1.00E-03	-7.27757	646	22.54%	9288.6	20.10%
	Unknown(Homeobox)/Limb-p300-ChIP-Seq/Homer	1.00E-03	-9.024533	166	5.79%	1995.1	4.32%
	HOXB13(Homeobox)/ProstateTumor-HOXB13-ChIP-Seq(GSE56288)	1.00E-03	-6.925214	256	8.93%	3403.7	7.36%
26	ETS:E-box(ETS,bHLH)/HPC7-Scl-ChIP-Seq(GSE22178)/Homer	1.00E-06	-14.445709	78	2.72%	685.9	1.48%
27	WIP5(C2H2)/colamp-WIP5-DAP-Seq(GSE60143)/Homer	1.00E-06	-14.074626	628	21.91%	8477.1	18.34%
28	AT1G77200(AP2EREBP)/colamp-AT1G77200-DAP-Seq(GSE60143)/	1.00E-06	-13.940708	294	10.26%	3582.8	7.75%
	At4g16750(AP2EREBP)/col-At4g16750-DAP-Seq(GSE60143)/Homer	1.00E-05	-13.419007	280	9.77%	3407.6	7.37%
	CBF2(AP2EREBP)/colamp-CBF2-DAP-Seq(GSE60143)/Homer	1.00E-05	-13.782841	187	6.52%	2103.1	4.55%
	CBF4(AP2EREBP)/colamp-CBF4-DAP-Seq(GSE60143)/Homer	1.00E-05	-12.060083	261	9.11%	3199.8	6.92%
	AT1G12630(AP2EREBP)/colamp-AT1G12630-DAP-Seq(GSE60143)/	1.00E-04	-9.537946	203	7.08%	2493.5	5.40%
	AT3G60490(AP2EREBP)/colamp-AT3G60490-DAP-Seq(GSE60143)/	1.00E-04	-9.26546	117	4.08%	1312.4	2.84%
	CBF3(AP2EREBP)/colamp-CBF3-DAP-Seq(GSE60143)/Homer	1.00E-04	-10.584085	184	6.42%	2181.8	4.72%
	CBF1(AP2EREBP)/colamp-CBF1-DAP-Seq(GSE60143)/Homer	1.00E-04	-10.490133	264	9.21%	3317.9	7.18%
	ARF2(ARF)/col-ARF2-DAP-Seq(GSE60143)/Homer	1.00E-03	-8.841271	1204	42.01%	17876.9	38.68%
	Rap210(AP2EREBP)/col-Rap210-DAP-Seq(GSE60143)/Homer	1.00E-03	-8.067718	223	7.78%	2853.8	6.18%
	DDF1(AP2EREBP)/col-DDF1-DAP-Seq(GSE60143)/Homer	1.00E-02	-6.505827	142	4.95%	1771.3	3.83%
29	Lhx1(Homeobox)/EmbryoCarcinoma-Lhx1-ChIP-Seq(GSE70957)/Hor	1.00E-06	-15.165519	292	10.19%	3501.5	7.58%
	Lhx2(Homeobox)/HFSC-Lhx2-ChIP-Seq(GSE48068)/Homer	1.00E-05	-11.859887	277	9.67%	3436.2	7.44%
	Dlx3(Homeobox)/Kerainocytes-Dlx3-ChIP-Seq(GSE89884)/Homer	1.00E-04	-10.597757	164	5.72%	1904.6	4.12%
	Lhx3(Homeobox)/Neuron-Lhx3-ChIP-Seq(GSE31456)/Homer	1.00E-04	-11.006582	407	14.20%	5374.8	11.63%
	ATHB23(ZFHD)/col-ATHB23-DAP-Seq(GSE60143)/Homer	1.00E-03	-7.503401	206	7.19%	2638.8	5.71%
	ATHB33(ZFHD)/col-ATHB33-DAP-Seq(GSE60143)/Homer	1.00E-03	-8.36909	305	10.64%	4033.9	8.73%
	ATHB25(ZFHD)/colamp-ATHB25-DAP-Seq(GSE60143)/Homer	1.00E-03	-7.964917	255	8.90%	3324.8	7.19%
	AT1G20910(ARID)/col-AT1G20910-DAP-Seq(GSE60143)/Homer	1.00E-02	-4.888764	301	10.50%	4230.8	9.15%
	ATHB24(ZFHD)/colamp-ATHB24-DAP-Seq(GSE60143)/Homer	1.00E-02	-4.987669	163	5.69%	2159.9	4.67%
30	PAX5(Paired,Homeobox)/GM12878-PAX5-ChIP-Seq(GSE32465)/Hor	1.00E-06	-14.160838	292	10.19%	3544.1	7.67%
	Pax8(Paired,Homeobox)/Thyroid-Pax8-ChIP-Seq(GSE26938)/Homer	1.00E-04	-11.179776	300	10.47%	3800.1	8.22%
	PAX6(Paired,Homeobox)/Forebrain-Pax6-ChIP-Seq(GSE66961)/Hom	1.00E-03	-8.065582	49	1.71%	462.6	1.00%

Cluster	Name	PValue	log(PValue)	# Target Seq	% of Targets	# Background Sequences with Motif	% of Background Sequences with Motif
1	NeuroD1(bHLH)/Islet-NeuroD1-ChIP-Seq(GSE30298)/Ho	1.00E-241	-555.32544	1454	35.27%	6562.9	14.48%
	NeuroG2(bHLH)/Fibroblast-NeuroG2-ChIP-Seq(GSE759	1.00E-226	-520.63902	2025	49.11%	11656.8	25.71%
	Atoh1(bHLH)/Cerebellum-Atoh1-ChIP-Seq(GSE22111)/I	1.00E-221	-510.10459	1699	41.21%	8866.5	19.56%
	Olig2(bHLH)/Neuron-Olig2-ChIP-Seq(GSE30882)/Home	1.00E-203	-468.45069	2150	52.15%	13330.9	29.41%
	TCF4(bHLH)/SHSY5Y-TCF4-ChIP-Seq(GSE96915)/Homer	1.00E-198	-456.32015	1975	47.90%	11792.3	26.01%
	Ascl1(bHLH)/NeuralTubes-Ascl1-ChIP-Seq(GSE55840)/I	1.00E-84	-194.48572	1803	43.73%	13306.7	29.35%
	Tcf21(bHLH)/ArterySmoothMuscle-Tcf21-ChIP-Seq(GSE	1.00E-70	-161.73437	1165	28.26%	7743.1	17.08%
	Ptf1a(bHLH)/Panc1-Ptf1a-ChIP-Seq(GSE47459)/Homer	1.00E-54	-124.67987	2365	57.36%	20515.1	45.26%
	HLH-1(bHLH)/cElegans-Embryo-HLH1-ChIP-Seq(modEnc	1.00E-51	-118.6611	830	20.13%	5368	11.84%
	SCL(bHLH)/HPC7-Scl-ChIP-Seq(GSE13511)/Homer	1.00E-44	-102.76816	3176	77.03%	30383.7	67.02%
	Ap4(bHLH)/AML-Tfap4-ChIP-Seq(GSE45738)/Homer	1.00E-33	-76.949565	1225	29.71%	9800.5	21.62%
	bZIP52(bZIP)/colamp-bZIP52-DAP-Seq(GSE60143)/Hom	1.00E-30	-69.088147	1115	27.04%	8904.7	19.64%
	MyoD(bHLH)/Myotube-MyoD-ChIP-Seq(GSE21614)/Hor	1.00E-30	-70.155116	866	21.00%	6497	14.33%
	HEB(bHLH)/mES-Heb-ChIP-Seq(GSE53233)/Homer	1.00E-30	-69.996974	1807	43.83%	15911.9	35.10%
	Myf5(bHLH)/GM-Myf5-ChIP-Seq(GSE24852)/Homer	1.00E-30	-71.127259	779	18.89%	5669.7	12.51%
	E-box/Drosophila-Promoters/Homer	1.00E-28	-64.966834	308	7.47%	1700.6	3.75%
	Tcf12(bHLH)/GM12878-Tcf12-ChIP-Seq(GSE32465)/Hor	1.00E-24	-55.393601	1020	24.74%	8308.3	18.33%
	VIP1(bZIP)/col-VIP1-DAP-Seq(GSE60143)/Homer	1.00E-22	-52.193511	227	5.51%	1209.1	2.67%
	MyoG(bHLH)/C2C12-MyoG-ChIP-Seq(GSE36024)/Home	1.00E-22	-51.988683	1052	25.52%	8712.1	19.22%
	bZIP69(bZIP)/col-bZIP69-DAP-Seq(GSE60143)/Homer	1.00E-18	-43.295141	155	3.76%	758.2	1.67%
	bZIP18(bZIP)/colamp-bZIP18-DAP-Seq(GSE60143)/Hom	1.00E-13	-29.995793	3077	74.63%	31473	69.43%
	E2A(bHLH)/proBcell-E2A-ChIP-Seq(GSE21978)/Homer	1.00E-05	-13.579367	1278	31.00%	12543.4	27.67%
2	Tal1	1.00E-147	-339.78158	1702	41.28%	10462.8	23.08%
	ZBTB18(Zf)/HEK293-ZBTB18.GFP-ChIP-Seq(GSE58341)	1.00E-28	-65.628803	553	13.41%	3732.5	8.23%
3	EBF2(EBF)/BrownAdipose-EBF2-ChIP-Seq(GSE97114)/I	1.00E-137	-316.20732	1311	31.80%	7273.6	16.05%
	EBF(EBF)/proBcell-EBF-ChIP-Seq(GSE21978)/Homer	1.00E-84	-193.59918	448	10.87%	1724.4	3.80%
	EBF1(EBF)/Near-E2A-ChIP-Seq(GSE21512)/Homer	1.00E-64	-148.68105	1314	31.87%	9310.9	20.54%
4	CTCF(Zf)/CD4+-CTCF-ChIP-Seq(Barski_et_al.)/Homer	1.00E-68	-158.70777	252	6.11%	728.6	1.61%
	BORIS(Zf)/K562-CTCFL-ChIP-Seq(GSE32465)/Homer	1.00E-50	-116.2111	300	7.28%	1226.6	2.71%
5	FOXA1(Forkhead)/LNCAP-FOXA1-ChIP-Seq(GSE27824)/	1.00E-60	-140.27003	817	19.82%	4977.6	10.98%
	Foxa2(Forkhead)/Liver-Foxa2-ChIP-Seq(GSE25694)/Hor	1.00E-59	-136.36476	652	15.81%	3661.3	8.08%
	FOXA1(Forkhead)/MCF7-FOXA1-ChIP-Seq(GSE26831)/I	1.00E-53	-122.2973	661	16.03%	3889.8	8.58%
	FOXM1(Forkhead)/MCF7-FOXM1-ChIP-Seq(GSE72977),	1.00E-47	-108.80088	696	16.88%	4347.3	9.59%
	Fox:Ebox(Forkhead,bHLH)/Panc1-Foxa2-ChIP-Seq(GSE4	1.00E-46	-106.65503	836	20.28%	5593.4	12.34%
	PHA-4(Forkhead)/cElegans-Embryos-PHA4-ChIP-Seq(m	1.00E-45	-104.18902	1886	45.74%	15864.4	35.00%
	Foxo3(Forkhead)/U2OS-Foxo3-ChIP-Seq(E-MTAB-2701	1.00E-43	-100.31055	541	13.12%	3162.6	6.98%
	FoxL2(Forkhead)/Ovary-FoxL2-ChIP-Seq(GSE60858)/Ho	1.00E-38	-89.336346	521	12.64%	3129.9	6.90%
	FOXK1(Forkhead)/HEK293-FOXK1-ChIP-Seq(GSE51673)	1.00E-37	-86.075014	686	16.64%	4580.5	10.10%
	FOXP1(Forkhead)/H9-FOXP1-ChIP-Seq(GSE31006)/Hon	1.00E-34	-78.46124	327	7.93%	1710.5	3.77%
	Foxf1(Forkhead)/Lung-Foxf1-ChIP-Seq(GSE77951)/Hom	1.00E-31	-73.10987	525	12.73%	3377.3	7.45%
	Foxo1(Forkhead)/RAW-Foxo1-ChIP-Seq(Fan_et_al.)/Hc	1.00E-30	-69.367821	1306	31.68%	10785.8	23.79%
	Foxa3(Forkhead)/Liver-Foxa3-ChIP-Seq(GSE77670)/Hor	1.00E-28	-64.791362	248	6.02%	1247.5	2.75%
	FOXK2(Forkhead)/U2OS-FOXK2-ChIP-Seq(E-MTAB-220	1.00E-24	-57.29597	459	11.13%	3045.2	6.72%
6	Lhx2(Homeobox)/HFSC-Lhx2-ChIP-Seq(GSE48068)/Hom	1.00E-57	-131.918	786	19.06%	4817.4	10.63%
	Lhx3(Homeobox)/Neuron-Lhx3-ChIP-Seq(GSE31456)/Hc	1.00E-53	-122.12387	1082	26.24%	7576.5	16.71%
	Lhx1(Homeobox)/EmbryoCarcinoma-Lhx1-ChIP-Seq(GSI	1.00E-51	-119.32726	781	18.94%	4935.4	10.89%
	Dlx3(Homeobox)/Kerainocytes-Dlx3-ChIP-Seq(GSE8988	1.00E-41	-95.591584	478	11.59%	2708.2	5.97%
	ATHB34(ZFHD)/colamp-ATHB34-DAP-Seq(GSE60143)/I	1.00E-36	-83.486513	536	13.00%	3328.1	7.34%
	ATHB23(ZFHD)/col-ATHB23-DAP-Seq(GSE60143)/Hom	1.00E-34	-78.758165	578	14.02%	3750.6	8.27%
	ATHB24(ZFHD)/colamp-ATHB24-DAP-Seq(GSE60143)/I	1.00E-33	-77.790294	490	11.88%	3018.1	6.66%
	ATHB33(ZFHD)/col-ATHB33-DAP-Seq(GSE60143)/Hom	1.00E-32	-74.258916	784	19.02%	5657.9	12.48%
	ATHB25(ZFHD)/colamp-ATHB25-DAP-Seq(GSE60143)/I	1.00E-29	-67.945125	680	16.49%	4824.1	10.64%
	AT1G20910(ARID)/col-AT1G20910-DAP-Seq(GSE60143	1.00E-15	-35.835304	748	18.14%	6175.6	13.62%
7	Rfx2(HTH)/LoVo-RFX2-ChIP-Seq(GSE49402)/Homer	1.00E-43	-99.404932	155	3.76%	441.2	0.97%
	RFX(HTH)/K562-RFX3-ChIP-Seq(SRA012198)/Homer	1.00E-40	-93.763097	140	3.40%	384.5	0.85%
	Rfx1(HTH)/NPC-H3K4me1-ChIP-Seq(GSE16256)/Homer	1.00E-34	-79.541146	254	6.16%	1168.4	2.58%
	X-box(HTH)/NPC-H3K4me1-ChIP-Seq(GSE16256)/Home	1.00E-27	-62.651155	150	3.64%	586.2	1.29%
8	NF1-halfsite(CTF)/LNCaP-NF1-ChIP-Seq(Unpublished)/	1.00E-43	-99.736594	1635	39.66%	13380.7	29.52%
	HIC1(Zf)/Treg-ZBTB29-ChIP-Seq(GSE99889)/Homer	1.00E-24	-55.383523	1762	42.74%	15879.8	35.03%
9	Nkx6.1(Homeobox)/Islet-Nkx6.1-ChIP-Seq(GSE40975)/I	1.00E-34	-78.79963	1468	35.61%	12179.1	26.87%
	Isl1(Homeobox)/Neuron-Isl1-ChIP-Seq(GSE31456)/Hom	1.00E-28	-64.796011	1174	28.47%	9587.3	21.15%
	Nanog(Homeobox)/mES-Nanog-ChIP-Seq(GSE11724)/H	1.00E-20	-47.136237	2419	58.67%	23292.5	51.38%
	Barx1(Homeobox)/Stomach-Barx1.3xFlag-ChIP-Seq(GS	1.00E-11	-25.443534	265	6.43%	1889.1	4.17%
10	Rfx5(HTH)/GM12878-Rfx5-ChIP-Seq(GSE31477)/Home	1.00E-33	-76.100089	317	7.69%	1657.1	3.66%
	Rfx6(HTH)/Min6b1-Rfx6.HA-ChIP-Seq(GSE62844)/Hom	1.00E-12	-28.989236	831	20.16%	7206.1	15.90%
11	NF1(CTF)/LNCAP-NF1-ChIP-Seq(Unpublished)/Homer	1.00E-27	-62.220822	470	11.40%	3065.1	6.76%
	Tlx?(NR)/NPC-H3K4me1-ChIP-Seq(GSE16256)/Homer	1.00E-25	-59.580901	512	12.42%	3470.6	7.66%
12	p53(p53)/Saos-p53-ChIP-Seq(GSE15780)/Homer	1.00E-24	-57.359961	129	3.13%	485.6	1.07%
	p53(p53)/Saos-p53-ChIP-Seq/Homer	1.00E-24	-57.359961	129	3.13%	485.6	1.07%
	p63(p53)/Keratinocyte-p63-ChIP-Seq(GSE17611)/Home	1.00E-19	-44.162153	332	8.05%	2157.5	4.76%
	p73(p53)/Trachea-p73-ChIP-Seq(PRJNA310161)/Homer	1.00E-17	-40.532607	80	1.94%	278.3	0.61%
	p53(p53)/mES-cMyc-ChIP-Seq(GSE11431)/Homer	1.00E-02	-6.367366	23	0.56%	126.5	0.28%
13	Tgif2(Homeobox)/mES-Tgif2-ChIP-Seq(GSE55404)/Hor	1.00E-23	-53.325007	2150	52.15%	20109	44.36%
	Tgif1(Homeobox)/mES-Tgif1-ChIP-Seq(GSE55404)/Hor	1.00E-12	-29.557101	1910	46.33%	18448.7	40.70%
	Meis1(Homeobox)/MastCells-Meis1-ChIP-Seq(GSE4808	1.00E-10	-23.383307	1209	29.32%	11293.1	24.91%
	Tbx20(T-box)/Heart-Tbx20-ChIP-Seq(GSE29636)/Home	1.00E-06	-15.418607	193	4.68%	1447.9	3.19%
14	MYB101(MYB)/colamp-MYB101-DAP-Seq(GSE60143)/I	1.00E-22	-51.466002	1136	27.55%	9561.3	21.09%
	MYB65(MYB)/colamp-MYB65-DAP-Seq(GSE60143)/Ho	1.00E-13	-31.661416	598	14.50%	4840.6	10.68%
	BMYB(HTH)/Hela-BMYB-ChIP-Seq(GSE27030)/Homer	1.00E-11	-26.440787	888	21.54%	7869.5	17.36%
	MYB73(MYB)/col-MYB73-DAP-Seq(GSE60143)/Homer	1.00E-06	-15.280078	908	22.02%	8558.7	18.88%
	MYB33(MYB)/col-MYB33-DAP-Seq(GSE60143)/Homer	1.00E-06	-13.845299	803	19.48%	7546.5	16.65%
	MYB81(MYB)/col-MYB81-DAP-Seq(GSE60143)/Homer	1.00E-05	-12.517525	669	16.23%	6235.1	13.75%
	MYB70(MYB)/col-MYB70-DAP-Seq(GSE60143)/Homer	1.00E-05	-12.48316	670	16.25%	6247.8	13.78%
	AT3G10030(Trihelix)/colamp-AT3G10030-DAP-Seq(GSI	1.00E-04	-9.262383	255	6.18%	2210.1	4.88%
	MYB56(MYB)/colamp-MYB56-DAP-Seq(GSE60143)/Ho	1.00E-03	-8.277843	347	8.42%	3167.4	6.99%
	MYB119(MYB)/colamp-MYB119-DAP-Seq(GSE60143)/I	1.00E-02	-6.748031	119	2.89%	977.6	2.16%
	MYB98(MYB)/col-MYB98-DAP-Seq(GSE60143)/Homer	1.00E-02	-5.769339	120	2.91%	1017.8	2.25%
	MYB118(MYB)/colamp-MYB118-DAP-Seq(GSE60143)/I	1.00E-02	-5.44207	120	2.91%	1028.8	2.27%

	MYB105(MYB)/colamp-MYB105-DAP-Seq(GSE60143)/	1.00E-02	-6.413887	239	5.80%	2167.4	4.78%
15	Unknown-ESC-element(?)/mES-Nanog-ChIP-Seq(GSE1	1.00E-18	-42.782853	661	16.03%	5160.7	11.38%
	Zic(Zf)/Cerebellum-ZIC1.2-ChIP-Seq(GSE60731)/Homer	1.00E-15	-36.147983	773	18.75%	6410.5	14.14%
	Zic3(Zf)/mES-Zic3-ChIP-Seq(GSE37889)/Homer	1.00E-12	-29.692163	636	15.43%	5262.9	11.61%
16	Pitx1:Ebox(Homeobox,bHLH)/Hindlimb-Pitx1-ChIP-Seq(	1.00E-16	-37.229527	154	3.74%	806.3	1.78%
17	c-Jun-CRE(bZIP)/K562-cJun-ChIP-Seq(GSE31477)/Home	1.00E-16	-37.614235	238	5.77%	1464.1	3.23%
	Atf7(bZIP)/3T3L1-Atf7-ChIP-Seq(GSE56872)/Homer	1.00E-15	-36.457523	330	8.00%	2263.1	4.99%
	JunD(bZIP)/K562-JunD-ChIP-Seq/Homer	1.00E-15	-34.799109	83	2.01%	328.4	0.72%
	Atf2(bZIP)/3T3L1-Atf2-ChIP-Seq(GSE56872)/Homer	1.00E-14	-33.142879	246	5.97%	1594.1	3.52%
	Atf1(bZIP)/K562-ATF1-ChIP-Seq(GSE31477)/Homer	1.00E-14	-33.673523	431	10.45%	3222.2	7.11%
	bZIP50(bZIP)/colamp-bZIP50-DAP-Seq(GSE60143)/Hom	1.00E-12	-28.533479	540	13.10%	4369.2	9.64%
	TGA6(bZIP)/colamp-TGA6-DAP-Seq(GSE60143)/Homer	1.00E-10	-23.027659	333	8.08%	2558.1	5.64%
	TGA5(bZIP)/col-TGA5-DAP-Seq(GSE60143)/Homer	1.00E-09	-22.821962	65	1.58%	290	0.64%
	TGA4(bZIP)/colamp-TGA4-DAP-Seq(GSE60143)/Homer	1.00E-09	-21.85858	174	4.22%	1158.1	2.55%
	CRE(bZIP)/Promoter/Homer	1.00E-08	-19.237513	160	3.88%	1081.9	2.39%
	TGA1(bZIP)/colamp-TGA1-DAP-Seq(GSE60143)/Homer	1.00E-08	-18.96247	234	5.68%	1742.2	3.84%
	TGA3(bZIP)/colamp-TGA3-DAP-Seq(GSE60143)/Homer	1.00E-05	-12.144188	38	0.92%	184.5	0.41%
	FEA4(bZIP)/Corn-FEA4-ChIP-Seq(GSE61954)/Homer	1.00E-04	-9.971986	628	15.23%	5949.9	13.13%
18	RBPJ:Ebox(?,bHLH)/Panc1-Rbpj1-ChIP-Seq(GSE47459)/	1.00E-14	-33.816132	359	8.71%	2567.3	5.66%
19	Hoxd13(Homeobox)/ChickenMSG-Hoxd13.Flag-ChIP-Se	1.00E-14	-34.2219	809	19.62%	6820.4	15.05%
	PABPC1(?)/MEL-PABC1-CLIP-Seq(GSE69755)/Homer	1.00E-11	-26.737593	819	19.86%	7164.3	15.80%
	HOXD13(Homeobox)/Chicken-Hoxd13-ChIP-Seq(GSE38	1.00E-11	-27.351217	502	12.18%	4038.2	8.91%
	Unknown(Homeobox)/Limb-p300-ChIP-Seq/Homer	1.00E-09	-22.652532	350	8.49%	2725.3	6.01%
	Hoxa13(Homeobox)/ChickenMSG-Hoxa13.Flag-ChIP-Se	1.00E-08	-19.56574	1181	28.64%	11183.7	24.67%
	HOXB13(Homeobox)/ProstateTumor-HOXB13-ChIP-Seq	1.00E-08	-19.820669	512	12.42%	4359.6	9.62%
	Hoxd11(Homeobox)/ChickenMSG-Hoxd11.Flag-ChIP-Se	1.00E-08	-19.855261	1222	29.64%	11598.5	25.59%
	Hoxa9(Homeobox)/ChickenMSG-Hoxa9.Flag-ChIP-Seq(	1.00E-08	-20.240414	1451	35.19%	13995.6	30.87%
	Hoxa11(Homeobox)/ChickenMSG-Hoxa11.Flag-ChIP-Se	1.00E-07	-16.189177	1131	27.43%	10836.4	23.90%
	CDX4(Homeobox)/ZebrafishEmbryos-Cdx4.Myc-ChIP-Se	1.00E-07	-18.304785	413	10.02%	3444.1	7.60%
	caudal(Homeobox)/Drosophila-Embryos-ChIP-Chip(mod	1.00E-05	-11.729106	354	8.59%	3092.9	6.82%
	Cdx2(Homeobox)/mES-Cdx2-ChIP-Seq(GSE14586)/Hom	1.00E-04	-10.129786	282	6.84%	2443.7	5.39%
20	Smad4(MAD)/ESC-SMAD4-ChIP-Seq(GSE29422)/Home	1.00E-13	-30.90064	1193	28.94%	10812.3	23.85%
	Smad2(MAD)/ES-SMAD2-ChIP-Seq(GSE29422)/Homer	1.00E-11	-26.906174	1144	27.75%	10466.3	23.09%
	Smad3(MAD)/NPC-Smad3-ChIP-Seq(GSE36673)/Home	1.00E-10	-24.68187	1872	45.40%	18274	40.31%
21	TGA10(bZIP)/colamp-TGA10-DAP-Seq(GSE60143)/Hom	1.00E-13	-31.608715	353	8.56%	2556.2	5.64%
	ATAF1(NAC)/col-ATAF1-DAP-Seq(GSE60143)/Homer	1.00E-06	-14.896726	1116	27.07%	10751.7	23.72%
	TGA9(bZIP)/colamp-TGA9-DAP-Seq(GSE60143)/Homer	1.00E-06	-14.85263	652	15.81%	5950.6	13.13%
	TGA2(bZIP)/colamp-TGA2-DAP-Seq(GSE60143)/Homer	1.00E-05	-11.950224	281	6.82%	2370.7	5.23%
22	AP-2alpha(AP2)/Hela-AP2alpha-ChIP-Seq(GSE31477)/H	1.00E-12	-29.006237	775	18.80%	6648.4	14.67%
	AP-2gamma(AP2)/MCF7-TFAP2C-ChIP-Seq(GSE21234),	1.00E-09	-22.139117	948	22.99%	8650.2	19.08%
23	MafA(bZIP)/Islet-MafA-ChIP-Seq(GSE30298)/Homer	1.00E-12	-29.740913	664	16.10%	5533.8	12.21%
	BATF(bZIP)/Th17-BATF-ChIP-Seq(GSE39756)/Homer	1.00E-10	-23.368189	400	9.70%	3176.2	7.01%
	JunB(bZIP)/DendriticCells-Junb-ChIP-Seq(GSE36099)/H	1.00E-10	-23.072739	354	8.59%	2753	6.07%
	Atf3(bZIP)/GBM-ATF3-ChIP-Seq(GSE33912)/Homer	1.00E-09	-21.338632	403	9.77%	3259.5	7.19%
	Fra1(bZIP)/BT549-Fra1-ChIP-Seq(GSE46166)/Homer	1.00E-09	-21.404825	344	8.34%	2700.5	5.96%
	AP-1(bZIP)/ThioMac-PU.1-ChIP-Seq(GSE21512)/Homer	1.00E-08	-19.891706	459	11.13%	3840.6	8.47%
	Fra2(bZIP)/Striatum-Fra2-ChIP-Seq(GSE43429)/Homer	1.00E-07	-17.176975	303	7.35%	2425.1	5.35%
	Jun-AP1(bZIP)/K562-cJun-ChIP-Seq(GSE31477)/Homer	1.00E-06	-14.719093	161	3.90%	1173.2	2.59%
	Fosl2(bZIP)/3T3L1-Fosl2-ChIP-Seq(GSE56872)/Homer	1.00E-04	-11.279494	209	5.07%	1700.9	3.75%
	Nrf2(bZIP)/Lymphoblast-Nrf2-ChIP-Seq(GSE37589)/Ho	1.00E-02	-6.253091	38	0.92%	249	0.55%
	NF-E2(bZIP)/K562-NFE2-ChIP-Seq(GSE31477)/Homer	1.00E-02	-5.772552	46	1.12%	326.7	0.72%
	Bach2(bZIP)/OCILy7-Bach2-ChIP-Seq(GSE44420)/Home	1.00E-02	-5.318041	130	3.15%	1130.6	2.49%
24	Pdx1(Homeobox)/Islet-Pdx1-ChIP-Seq(SRA008281)/Hor	1.00E-12	-29.029785	581	14.09%	4749.4	10.48%
	HOXA9(Homeobox)/HSC-Hoxa9-ChIP-Seq(GSE33509)/H	1.00E-10	-23.922772	383	9.29%	3002.4	6.62%
	LIN-39(Homeobox)/cElegans.L3-LIN39-ChIP-Seq(modEr	1.00E-09	-20.91597	602	14.60%	5208.9	11.49%
	PBX2(Homeobox)/K562-PBX2-ChIP-Seq(Encode)/Homer	1.00E-09	-21.670683	456	11.06%	3757.7	8.29%
	Hoxc9(Homeobox)/Ainv15-Hoxc9-ChIP-Seq(GSE21812)/	1.00E-09	-22.664473	290	7.03%	2171	4.79%
	Hoxb4(Homeobox)/ES-Hoxb4-ChIP-Seq(GSE34014)/Hor	1.00E-07	-16.272294	134	3.25%	907.9	2.00%
	HOXA1(Homeobox)/mES-Hoxa1-ChIP-Seq(SRP084292)/	1.00E-04	-10.416261	162	3.93%	1281.7	2.83%
25	GLIS3(Zf)/Thyroid-Glis3.GFP-ChIP-Seq(GSE103297)/Ho	1.00E-11	-27.297235	1222	29.64%	11257.9	24.83%
26	AARE(HLH)/mES-cMyc-ChIP-Seq/Homer	1.00E-11	-26.899534	85	2.06%	396.4	0.87%
	Atf4(bZIP)/MEF-Atf4-ChIP-Seq(GSE35681)/Homer	1.00E-09	-21.875193	188	4.56%	1278.3	2.82%
	Chop(bZIP)/MEF-Chop-ChIP-Seq(GSE35681)/Homer	1.00E-09	-21.663305	152	3.69%	975.6	2.15%
	CEBP:AP1(bZIP)/ThioMac-CEBPb-ChIP-Seq(GSE21512)/	1.00E-06	-15.842374	433	10.50%	3720.9	8.21%
27	Sox10(HMG)/SciaticNerve-Sox3-ChIP-Seq(GSE35132)/H	1.00E-11	-25.939955	985	23.89%	8873.3	19.57%
	Sox2(HMG)/mES-Sox2-ChIP-Seq(GSE11431)/Homer	1.00E-11	-26.39694	539	13.07%	4419.3	9.75%
	Sox17(HMG)/Endoderm-Sox17-ChIP-Seq(GSE61475)/H	1.00E-11	-25.857707	453	10.99%	3612.3	7.97%
	Sox3(HMG)/NPC-Sox3-ChIP-Seq(GSE33059)/Homer	1.00E-09	-22.840072	994	24.11%	9092.1	20.06%
	Sox15(HMG)/CPA-Sox15-ChIP-Seq(GSE62909)/Homer	1.00E-09	-21.673589	617	14.96%	5330.8	11.76%
	Sox6(HMG)/Myotubes-Sox6-ChIP-Seq(GSE32627)/Hom	1.00E-09	-21.089128	898	21.78%	8183	18.05%
	Sox4(HMG)/proB-Sox4-ChIP-Seq(GSE50066)/Homer	1.00E-07	-17.168982	525	12.73%	4580.5	10.10%
	Sox9(HMG)/Limb-SOX9-ChIP-Seq(GSE73225)/Homer	1.00E-05	-13.571863	567	13.75%	5146.4	11.35%
28	ANL2(HB)/col-ANL2-DAP-Seq(GSE60143)/Homer	1.00E-10	-24.879328	285	6.91%	2079.3	4.59%
	HDG1(Homeobox)/col100-HDG1-DAP-Seq(GSE60143)/I	1.00E-07	-17.473918	239	5.80%	1821.3	4.02%
29	ARF2(ARF)/col-ARF2-DAP-Seq(GSE60143)/Homer	1.00E-10	-24.509665	1726	41.86%	16710.5	36.86%
	DDF1(AP2EREBP)/col-DDF1-DAP-Seq(GSE60143)/Hom	1.00E-02	-4.787525	123	2.98%	1081.5	2.39%
	CBF2(AP2EREBP)/colamp-CBF2-DAP-Seq(GSE60143)/H	1.00E-02	-6.54857	139	3.37%	1175.1	2.59%
	CBF3(AP2EREBP)/colamp-CBF3-DAP-Seq(GSE60143)/H	1.00E-02	-6.04416	143	3.47%	1231.4	2.72%
	CBF1(AP2EREBP)/colamp-CBF1-DAP-Seq(GSE60143)/H	1.00E-02	-4.628706	191	4.63%	1768.4	3.90%
30	Nkx2.1(Homeobox)/LungAC-Nkx2.1-ChIP-Seq(GSE4325	1.00E-10	-23.246126	1712	41.52%	16624.6	36.67%
	Bapx1(Homeobox)/VertebralCol-Bapx1-ChIP-Seq(GSE3	1.00E-09	-22.597855	1274	30.90%	12010	26.49%
	Nkx2.2(Homeobox)/NPC-Nkx2.2-ChIP-Seq(GSE61673)/H	1.00E-08	-20.479024	1267	30.73%	12039.4	26.56%
	Nkx3.1(Homeobox)/LNCaP-Nkx3.1-ChIP-Seq(GSE28264)	1.00E-08	-20.70813	1331	32.28%	12701.5	28.02%
	Nkx2.5(Homeobox)/HL1-Nkx2.5.biotin-ChIP-Seq(GSE21	1.00E-07	-17.991458	1374	33.33%	13300.3	29.34%

ASCL1_NEUROD1_overlap

Cluster	Name	PValue	log(PValue)	# Target Seq	% of Targets	# Backgroun	% of Background Sequences with Motif
1	NeuroD1(bHLH)/Islet-NeuroD1-ChIP-Seq(GSE30298)/Homer	1.00E-75	-174.52443	1737	37.85%	10484.4	25.44%
	Atoh1(bHLH)/Cerebellum-Atoh1-ChIP-Seq(GSE22111)/Homer	1.00E-72	-168.04874	2187	47.66%	14272.4	34.63%
	Ascl1(bHLH)/NeuralTubes-Ascl1-ChIP-Seq(GSE55840)/Homer	1.00E-72	-166.40792	3038	66.20%	21867	53.06%
	Tcf12(bHLH)/GM12878-Tcf12-ChIP-Seq(GSE32465)/Homer	1.00E-63	-145.58959	2230	48.59%	15003.5	36.40%
	Tcf21(bHLH)/ArterySmoothMuscle-Tcf21-ChIP-Seq(GSE61369)/	1.00E-62	-144.39505	2047	44.61%	13473.8	32.69%
	NeuroG2(bHLH)/Fibroblast-NeuroG2-ChIP-Seq(GSE75910)/Hon	1.00E-59	-136.57646	2411	52.54%	16727.7	40.59%
	MyoG(bHLH)/C2C12-MyoG-ChIP-Seq(GSE36024)/Homer	1.00E-59	-138.01485	2211	48.18%	14971.6	36.33%
	Myf5(bHLH)/GM-Myf5-ChIP-Seq(GSE24852)/Homer	1.00E-58	-134.19041	1544	33.65%	9532.3	23.13%
	E2A(bHLH)/proBcell-E2A-ChIP-Seq(GSE21978)/Homer	1.00E-51	-118.6854	2896	63.11%	21426.2	51.99%
	Ap4(bHLH)/AML-Tfap4-ChIP-Seq(GSE45738)/Homer	1.00E-49	-113.35674	2267	49.40%	15910.9	38.61%
	HLH-1(bHLH)/cElegans-Embryo-HLH1-ChIP-Seq(modEncode)/Hc	1.00E-46	-106.54549	1259	27.44%	7721.7	18.74%
	TCF4(bHLH)/SHSY5Y-TCF4-ChIP-Seq(GSE96915)/Homer	1.00E-43	-99.468588	2370	51.65%	17094.3	41.48%
	Olig2(bHLH)/Neuron-Olig2-ChIP-Seq(GSE30882)/Homer	1.00E-42	-98.778918	2470	53.82%	17992.6	43.66%
	MyoD(bHLH)/Myotube-MyoD-ChIP-Seq(GSE21614)/Homer	1.00E-42	-98.76537	1788	38.96%	12125.9	29.42%
	HEB(bHLH)/mES-Heb-ChIP-Seq(GSE53233)/Homer	1.00E-40	-93.809198	3250	70.82%	25276.8	61.33%
	Ptf1a(bHLH)/Panc1-Ptf1a-ChIP-Seq(GSE47459)/Homer	1.00E-35	-81.495517	3582	78.06%	28779.6	69.83%
	bZIP52(bZIP)/colamp-bZIP52-DAP-Seq(GSE60143)/Homer	1.00E-23	-53.83936	1627	35.45%	11783.3	28.59%
	VIP1(bZIP)/col-VIP1-DAP-Seq(GSE60143)/Homer	1.00E-21	-50.036306	372	8.11%	1964.2	4.77%
	bZIP69(bZIP)/col-bZIP69-DAP-Seq(GSE60143)/Homer	1.00E-20	-47.006621	275	5.99%	1341.6	3.26%
	E-box/Drosophila-Promoters/Homer	1.00E-15	-35.967152	397	8.65%	2337.3	5.67%
	SCL(bHLH)/HPC7-Scl-ChIP-Seq(GSE13511)/Homer	1.00E-04	-11.44966	4014	87.47%	35146.1	85.28%
2	CTCF(Zf)/CD4+-CTCF-ChIP-Seq(Barski_et_al.)/Homer	1.00E-63	-146.55895	522	11.38%	2084.5	5.06%
	BORIS(Zf)/K562-CTCFL-ChIP-Seq(GSE32465)/Homer	1.00E-55	-127.14668	902	19.66%	4777.8	11.59%
3	NF1(CTF)/LNCAP-NF1-ChIP-Seq(Unpublished)/Homer	1.00E-56	-131.04517	1141	24.86%	6473.3	15.71%
	Tlx?(NR)/NPC-H3K4me1-ChIP-Seq(GSE16256)/Homer	1.00E-22	-51.107091	944	20.57%	6245.7	15.15%
4	Sp1(Zf)/Promoter/Homer	1.00E-56	-130.95318	1106	24.10%	6214	15.08%
	Sp5(Zf)/mES-Sp5.Flag-ChIP-Seq(GSE72989)/Homer	1.00E-16	-39.017521	2270	49.47%	17815.1	43.23%
5	KLF3(Zf)/MEF-Klf3-ChIP-Seq(GSE44748)/Homer	1.00E-40	-93.104495	1385	30.18%	8940.7	21.69%
	Klf9(Zf)/GBM-Klf9-ChIP-Seq(GSE62211)/Homer	1.00E-29	-67.583047	1106	24.10%	7195.2	17.46%
	Klf4(Zf)/mES-Klf4-ChIP-Seq(GSE11431)/Homer	1.00E-17	-40.592484	945	20.59%	6491	15.75%
	KLF10(Zf)/HEK293-KLF10.GFP-ChIP-Seq(GSE58341)/Homer	1.00E-12	-28.002483	1160	25.28%	8619.1	20.91%
	KLF6(Zf)/PDAC-KLF6-ChIP-Seq(GSE64557)/Homer	1.00E-07	-17.987671	2291	49.92%	18889.6	45.83%
	KLF5(Zf)/LoVo-KLF5-ChIP-Seq(GSE49402)/Homer	1.00E-05	-13.572017	2533	55.20%	21315	51.72%
	EKLF(Zf)/Erythrocyte-Klf1-ChIP-Seq(GSE20478)/Homer	1.00E-04	-11.057203	413	9.00%	3024.9	7.34%
6	Elk1(ETS)/Hela-Elk1-ChIP-Seq(GSE31477)/Homer	1.00E-38	-88.691843	1307	28.48%	8397.9	20.38%
	Elk4(ETS)/Hela-Elk4-ChIP-Seq(GSE31477)/Homer	1.00E-37	-85.38418	1302	28.37%	8417.2	20.42%
	ETS(ETS)/Promoter/Homer	1.00E-36	-83.434885	764	16.65%	4324	10.49%
	ELF1(ETS)/Jurkat-ELF1-ChIP-Seq(SRA014231)/Homer	1.00E-30	-70.419254	1168	25.45%	7632.7	18.52%
	Fli1(ETS)/CD8-FLI-ChIP-Seq(GSE20898)/Homer	1.00E-22	-50.957042	1865	40.64%	13893.6	33.71%
	GABPA(ETS)/Jurkat-GABPa-ChIP-Seq(GSE17954)/Homer	1.00E-22	-50.884217	1488	32.43%	10692.2	25.94%
	Elf4(ETS)/BMDM-Elf4-ChIP-Seq(GSE88699)/Homer	1.00E-19	-45.055223	1511	32.93%	11045.4	26.80%
	ETV4(ETS)/HepG2-ETV4-ChIP-Seq(ENCODE)/Homer	1.00E-16	-38.445222	1963	42.78%	15133.7	36.72%
	EHF(ETS)/LoVo-EHF-ChIP-Seq(GSE49402)/Homer	1.00E-16	-37.15503	1633	35.59%	12323.5	29.90%
	ETV1(ETS)/GIST48-ETV1-ChIP-Seq(GSE22441)/Homer	1.00E-13	-31.803967	2001	43.60%	15704.5	38.11%
	EWS:FLI1-fusion(ETS)/SK_N_MC-EWS:FLI1-ChIP-Seq(SRA0142:	1.00E-12	-27.638855	900	19.61%	6474	15.71%
	EWS:ERG-fusion(ETS)/CADO_ES1-EWS:ERG-ChIP-Seq(SRA0142	1.00E-10	-23.222817	806	17.56%	5837.9	14.17%
	ELF5(ETS)/T47D-ELF5-ChIP-Seq(GSE30407)/Homer	1.00E-10	-23.826337	948	20.66%	6990.2	16.96%
	SPDEF(ETS)/VCaP-SPDEF-ChIP-Seq(SRA014231)/Homer	1.00E-07	-16.391987	1371	29.88%	10882.6	26.41%
	ELF3(ETS)/PDAC-ELF3-ChIP-Seq(GSE64557)/Homer	1.00E-07	-17.863054	876	19.09%	6603.8	16.02%
	Ets1-distal(ETS)/CD4+-PolII-ChIP-Seq(Barski_et_al.)/Homer	1.00E-03	-9.102192	367	8.00%	2718.5	6.60%
7	FOXA1(Forkhead)/MCF7-FOXA1-ChIP-Seq(GSE26831)/Homer	1.00E-36	-84.037556	768	16.74%	4345.5	10.54%
	FOXA1(Forkhead)/LNCAP-FOXA1-ChIP-Seq(GSE27824)/Homer	1.00E-33	-78.177963	919	20.03%	5557.2	13.48%
	FOXM1(Forkhead)/MCF7-FOXM1-ChIP-Seq(GSE72977)/Homer	1.00E-29	-67.820095	805	17.54%	4860.7	11.79%
	Foxa2(Forkhead)/Liver-Foxa2-ChIP-Seq(GSE25694)/Homer	1.00E-28	-65.183308	724	15.78%	4294.7	10.42%
	Foxf1(Forkhead)/Lung-Foxf1-ChIP-Seq(GSE77951)/Homer	1.00E-25	-58.252122	656	14.30%	3896.2	9.45%
	FoxL2(Forkhead)/Ovary-FoxL2-ChIP-Seq(GSE60858)/Homer	1.00E-23	-54.960674	607	13.23%	3584.8	8.70%
	Foxa3(Forkhead)/Liver-Foxa3-ChIP-Seq(GSE77670)/Homer	1.00E-22	-51.181773	304	6.62%	1490	3.62%
	FOXP1(Forkhead)/H9-FOXP1-ChIP-Seq(GSE31006)/Homer	1.00E-22	-52.674316	402	8.76%	2140	5.19%
	Foxo3(Forkhead)/U2OS-Foxo3-ChIP-Seq(E-MTAB-2701)/Homer	1.00E-22	-51.743511	602	13.12%	3598.8	8.73%
	Fox:Ebox(Forkhead,bHLH)/Panc1-Foxa2-ChIP-Seq(GSE47459)/H	1.00E-18	-41.481452	997	21.73%	6889.4	16.72%
	FOXK1(Forkhead)/HEK293-FOXK1-ChIP-Seq(GSE51673)/Homer	1.00E-18	-41.73304	803	17.50%	5332.1	12.94%
	PHA-4(Forkhead)/cElegans-Embryos-PHA4-ChIP-Seq(modEncoc	1.00E-12	-27.948015	2241	48.83%	17978.1	43.62%
	FOXK2(Forkhead)/U2OS-FOXK2-ChIP-Seq(E-MTAB-2204)/Home	1.00E-11	-26.240789	564	12.29%	3803.2	9.23%
	Foxo1(Forkhead)/RAW-Foxo1-ChIP-Seq(Fan_et_al.)/Homer	1.00E-10	-23.892241	1815	39.55%	14395.9	34.93%
8	NRF1(NRF)/MCF7-NRF1-ChIP-Seq(Unpublished)/Homer	1.00E-27	-63.44888	499	10.87%	2685.8	6.52%
	NRF(NRF)/Promoter/Homer	1.00E-20	-48.146118	504	10.98%	2931.2	7.11%
9	NF1-halfsite(CTF)/LNCaP-NF1-ChIP-Seq(Unpublished)/Homer	1.00E-26	-60.930308	2604	56.74%	20120	48.82%
	HIC1(Zf)/Treg-ZBTB29-ChIP-Seq(GSE99889)/Homer	1.00E-09	-20.866556	3091	67.36%	26002.4	63.09%
10	SHN3(AP2EREBP)/col-SHN3-DAP-Seq(GSE60143)/Homer	1.00E-25	-58.083717	1257	27.39%	8607.9	20.89%
	RRTF1(AP2EREBP)/colamp-RRTF1-DAP-Seq(GSE60143)/Homer	1.00E-22	-51.107113	538	11.72%	3135.4	7.61%
	AT4G18450(AP2EREBP)/col-AT4G18450-DAP-Seq(GSE60143)/I	1.00E-21	-49.714805	1291	28.13%	9085.4	22.05%
	ERF9(AP2EREBP)/colamp-ERF9-DAP-Seq(GSE60143)/Homer	1.00E-21	-49.438141	924	20.14%	6122.7	14.86%
	RAP212(AP2EREBP)/col-RAP212-DAP-Seq(GSE60143)/Homer	1.00E-19	-44.97591	1643	35.80%	12166.8	29.52%
	CRF4(AP2EREBP)/colamp-CRF4-DAP-Seq(GSE60143)/Homer	1.00E-19	-45.072121	1548	33.73%	11357.6	27.56%
	ERF1(AP2EREBP)/colamp-ERF1-DAP-Seq(GSE60143)/Homer	1.00E-18	-41.578272	1695	36.94%	12713.6	30.85%
	ERF5(AP2EREBP)/colamp-ERF5-DAP-Seq(GSE60143)/Homer	1.00E-18	-42.1125	1350	29.42%	9777.7	23.72%
	ERF3(AP2EREBP)/colamp-ERF3-DAP-Seq(GSE60143)/Homer	1.00E-17	-40.386067	1586	34.56%	11819.9	28.68%
	ERF10(AP2EREBP)/col-ERF10-DAP-Seq(GSE60143)/Homer	1.00E-16	-38.584035	1646	35.87%	12388.4	30.06%
	ABR1(AP2EREBP)/colamp-ABR1-DAP-Seq(GSE60143)/Homer	1.00E-15	-35.21361	1837	40.03%	14148.2	34.33%
	ERF2(AP2EREBP)/colamp-ERF2-DAP-Seq(GSE60143)/Homer	1.00E-15	-36.716195	1807	39.38%	13837	33.57%
	ERF73(AP2EREBP)/col-ERF73-DAP-Seq(GSE60143)/Homer	1.00E-15	-35.266159	1795	39.12%	13782.1	33.44%
	ERF11(AP2EREBP)/col-ERF11-DAP-Seq(GSE60143)/Homer	1.00E-14	-34.127767	1810	39.44%	13952	33.85%
	ERF13(AP2EREBP)/colamp-ERF13-DAP-Seq(GSE60143)/Homer	1.00E-13	-30.875851	2072	45.15%	16364.7	39.71%
	ESE1(AP2EREBP)/col-ESE1-DAP-Seq(GSE60143)/Homer	1.00E-13	-31.982622	1901	41.43%	14822.7	35.97%
	ERF8(AP2EREBP)/colamp-ERF8-DAP-Seq(GSE60143)/Homer	1.00E-11	-25.84732	1929	42.04%	15310.5	37.15%
	ERF4(AP2EREBP)/colamp-ERF4-DAP-Seq(GSE60143)/Homer	1.00E-11	-25.744502	1935	42.17%	15367.1	37.29%
	RAP26(AP2EREBP)/colamp-RAP26-DAP-Seq(GSE60143)/Homer	1.00E-09	-21.218722	2065	45.00%	16716.7	40.56%
	ERF105(AP2EREBP)/colamp-ERF105-DAP-Seq(GSE60143)/Hom	1.00E-07	-17.140033	2458	53.56%	20432.8	49.58%

	ERF104(AP2EREBP)/col-ERF104-DAP-Seq(GSE60143)/Homer	1.00E-06	-15.964919	2117	46.13%	17448.3	42.34%
	ERF7(AP2EREBP)/col-ERF7-DAP-Seq(GSE60143)/Homer	1.00E-05	-13.568211	2140	46.63%	17793.6	43.17%
	ERF115(AP2EREBP)/colamp-ERF115-DAP-Seq(GSE60143)/Hom	1.00E-03	-7.832356	2373	51.71%	20286.2	49.22%
	At2g33710(AP2EREBP)/colamp-At2g33710-DAP-Seq(GSE6014	1.00E-03	-6.995776	2537	55.28%	21834.9	52.98%
	AT5G23930(mTERF)/col-AT5G23930-DAP-Seq(GSE60143)/Hon	1.00E-02	-5.190446	2862	62.37%	24945.4	60.53%
	AT1G28160(AP2EREBP)/colamp-AT1G28160-DAP-Seq(GSE601	1.00E-02	-5.049638	2673	58.25%	23251.7	56.42%
11	Slug(Zf)/Mesoderm-Snai2-ChIP-Seq(GSE61475)/Homer	1.00E-24	-56.928109	1448	31.55%	10205.6	24.76%
	E2A(bHLH),near_PU.1/Bcell-PU.1-ChIP-Seq(GSE21512)/Homer	1.00E-15	-35.835862	2536	55.26%	20308.6	49.28%
	ZEB1(Zf)/PDAC-ZEB1-ChIP-Seq(GSE64557)/Homer	1.00E-07	-16.189666	2601	56.68%	21775	52.84%
	ZEB2(Zf)/SNU398-ZEB2-ChIP-Seq(GSE103048)/Homer	1.00E-05	-12.768641	1806	39.35%	14881.8	36.11%
	NGA4(ABI3VP1)/col-NGA4-DAP-Seq(GSE60143)/Homer	1.00E-02	-5.935041	3065	66.79%	26714.1	64.82%
12	E2FA(E2FDP)/colamp-E2FA-DAP-Seq(GSE60143)/Homer	1.00E-23	-53.642685	956	20.83%	6287.8	15.26%
	E2F(E2F)/Hela-CellCycle-Expression/Homer	1.00E-08	-19.522302	182	3.97%	1034.3	2.51%
13	ZBTB18(Zf)/HEK293-ZBTB18.GFP-ChIP-Seq(GSE58341)/Homer	1.00E-23	-53.698037	936	20.40%	6128.1	14.87%
	Tal1	1.00E-14	-34.093417	1735	37.81%	13304.4	32.28%
14	LEP(AP2EREBP)/col-LEP-DAP-Seq(GSE60143)/Homer	1.00E-22	-51.541308	1061	23.12%	7169.2	17.40%
	PUCHI(AP2EREBP)/colamp-PUCHI-DAP-Seq(GSE60143)/Homer	1.00E-15	-34.648027	1702	37.09%	13000.2	31.54%
	CRF10(AP2EREBP)/col100-CRF10-DAP-Seq(GSE60143)/Homer	1.00E-07	-18.207711	2172	47.33%	17816.2	43.23%
15	Rfx2(HTH)/LoVo-RFX2-ChIP-Seq(GSE49402)/Homer	1.00E-21	-49.398825	239	5.21%	1085.6	2.63%
	RFX(HTH)/K562-RFX3-ChIP-Seq(SRA012198)/Homer	1.00E-21	-48.611305	219	4.77%	966.8	2.35%
	Rfx1(HTH)/NPC-H3K4me1-ChIP-Seq(GSE16256)/Homer	1.00E-16	-37.712779	391	8.52%	2266.5	5.50%
	X-box(HTH)/NPC-H3K4me1-ChIP-Seq(GSE16256)/Homer	1.00E-13	-31.25819	202	4.40%	1023.9	2.48%
16	CAMTA1(CAMTA)/col-CAMTA1-DAP-Seq(GSE60143)/Homer	1.00E-21	-48.999656	946	20.61%	6306.6	15.30%
	CAMTA5(CAMTA)/col-CAMTA5-DAP-Seq(GSE60143)/Homer	1.00E-03	-8.359673	339	7.39%	2516.5	6.11%
17	E2F4(E2F)/K562-E2F4-ChIP-Seq(GSE31477)/Homer	1.00E-20	-46.771759	1271	27.70%	8995.9	21.83%
	E2F6(E2F)/Hela-E2F6-ChIP-Seq(GSE31477)/Homer	1.00E-18	-42.658138	1467	31.97%	10743.5	26.07%
	DEL2(E2FDP)/col-DEL2-DAP-Seq(GSE60143)/Homer	1.00E-17	-39.4896	804	17.52%	5390.8	13.08%
	E2F3(E2F)/MEF-E2F3-ChIP-Seq(GSE71376)/Homer	1.00E-17	-41.070451	1632	35.56%	12190	29.58%
	E2F1(E2F)/Hela-E2F1-ChIP-Seq(GSE22478)/Homer	1.00E-17	-40.824396	829	18.06%	5558.7	13.49%
	E2F7(E2F)/Hela-E2F7-ChIP-Seq(GSE32673)/Homer	1.00E-11	-27.267966	418	9.11%	2648.6	6.43%
18	At5g65130(AP2EREBP)/colamp-At5g65130-DAP-Seq(GSE6014	1.00E-20	-48.112913	619	13.49%	3786.9	9.19%
	DREB19(AP2EREBP)/colamp-DREB19-DAP-Seq(GSE60143)/Hor	1.00E-15	-34.868074	847	18.46%	5844.7	14.18%
	At1g36060(AP2EREBP)/colamp-At1g36060-DAP-Seq(GSE6014	1.00E-11	-26.618619	1045	22.77%	7704	18.69%
	DEAR2(AP2EREBP)/colamp-DEAR2-DAP-Seq(GSE60143)/Home	1.00E-11	-26.838388	1529	33.32%	11795.1	28.62%
	At4g28140(AP2EREBP)/colamp-At4g28140-DAP-Seq(GSE6014	1.00E-10	-25.133761	638	13.90%	4419.8	10.72%
	DREB2(AP2EREBP)/col-DREB2-DAP-Seq(GSE60143)/Homer	1.00E-10	-23.479505	674	14.69%	4754.8	11.54%
	CEJ1(AP2EREBP)/col-CEJ1-DAP-Seq(GSE60143)/Homer	1.00E-08	-19.528107	1353	29.48%	10582.9	25.68%
	AT1G44830(AP2EREBP)/col-AT1G44830-DAP-Seq(GSE60143)/I	1.00E-08	-20.53015	656	14.30%	4695.3	11.39%
	DREB26(AP2EREBP)/col-DREB26-DAP-Seq(GSE60143)/Homer	1.00E-07	-17.528822	491	10.70%	3451.6	8.38%
	ERF38(AP2EREBP)/col-ERF38-DAP-Seq(GSE60143)/Homer	1.00E-06	-14.946887	697	15.19%	5222.1	12.67%
	AT1G71450(AP2EREBP)/col-AT1G71450-DAP-Seq(GSE60143)/I	1.00E-06	-16.059885	2174	47.37%	17950.2	43.55%
	bHLH10(bHLH)/colamp-bHLH10-DAP-Seq(GSE60143)/Homer	1.00E-05	-12.325111	403	8.78%	2900.8	7.04%
	AT3G16280(AP2EREBP)/colamp-AT3G16280-DAP-Seq(GSE601	1.00E-05	-13.674724	472	10.29%	3416.2	8.29%
	DEAR5(AP2EREBP)/col-DEAR5-DAP-Seq(GSE60143)/Homer	1.00E-05	-12.108854	265	5.77%	1804.1	4.38%
	At1g22810(AP2EREBP)/colamp-At1g22810-DAP-Seq(GSE6014	1.00E-05	-13.573684	655	14.27%	4926.7	11.95%
	At1g19210(AP2EREBP)/colamp-At1g19210-DAP-Seq(GSE6014	1.00E-04	-10.388186	1848	40.27%	15407.8	37.39%
	TINY(AP2EREBP)/col-TINY-DAP-Seq(GSE60143)/Homer	1.00E-04	-10.026497	356	7.76%	2595.7	6.30%
	At4g31060(AP2EREBP)/colamp-At4g31060-DAP-Seq(GSE6014	1.00E-04	-9.351587	521	11.35%	3984.9	9.67%
	DEAR3(AP2EREBP)/colamp-DEAR3-DAP-Seq(GSE60143)/Home	1.00E-04	-10.866826	454	9.89%	3367.1	8.17%
	At2g44940(AP2EREBP)/colamp-At2g44940-DAP-Seq(GSE6014	1.00E-03	-8.268527	267	5.82%	1932.4	4.69%
	At1g75490(AP2EREBP)/colamp-At1g75490-DAP-Seq(GSE6014	1.00E-03	-8.866542	2303	50.19%	19574.6	47.50%
	At1g77640(AP2EREBP)/col-At1g77640-DAP-Seq(GSE60143)/Hc	1.00E-02	-6.472161	339	7.39%	2593.9	6.29%
19	HINFP(Zf)/K562-HINFP.eGFP-ChIP-Seq(Encode)/Homer	1.00E-18	-42.345738	1063	23.16%	7403.2	17.96%
20	NFY(CCAAT)/Promoter/Homer	1.00E-17	-40.458845	957	20.85%	6591.2	15.99%
21	LOB(LOBAS2)/col-LOB-DAP-Seq(GSE60143)/Homer	1.00E-17	-40.274722	693	15.10%	4501.5	10.92%
	AS2(LOBAS2)/col-AS2-DAP-Seq(GSE60143)/Homer	1.00E-10	-25.087058	280	6.10%	1656.4	4.02%
	LBD19(LOBAS2)/colamp-LBD19-DAP-Seq(GSE60143)/Homer	1.00E-05	-12.815412	1891	41.21%	15629.3	37.92%
	ASL18(LOBAS2)/colamp-ASL18-DAP-Seq(GSE60143)/Homer	1.00E-03	-8.825688	2237	48.75%	18986.5	46.07%
22	LBD23(LOBAS2)/colamp-LBD23-DAP-Seq(GSE60143)/Homer	1.00E-16	-39.005247	1584	34.52%	11845.7	28.74%
23	YY1(Zf)/Promoter/Homer	1.00E-16	-38.632266	189	4.12%	864.2	2.10%
24	Ronin(THAP)/ES-Thap11-ChIP-Seq(GSE51522)/Homer	1.00E-16	-37.31677	111	2.42%	405.5	0.98%
	GFY-Staf(?,Zf)/Promoter/Homer	1.00E-13	-31.136613	133	2.90%	578.9	1.40%
25	Rfx5(HTH)/GM12878-Rfx5-ChIP-Seq(GSE31477)/Homer	1.00E-16	-37.356329	472	10.29%	2869.8	6.96%
	Rfx6(HTH)/Min6b1-Rfx6.HA-ChIP-Seq(GSE62844)/Homer	1.00E-11	-25.411261	1716	37.39%	13468.8	32.68%
26	DPL-1(E2F)/cElegans-Adult-ChIP-Seq(modEncode)/Homer	1.00E-16	-38.563559	1673	36.46%	12620.9	30.62%
	FHY3(FAR1)/Arabidopsis-FHY3-ChIP-Seq(GSE30711)/Homer	1.00E-08	-19.464988	771	16.80%	5672.5	13.76%
	FAR1(FAR1)/col-FAR1-DAP-Seq(GSE60143)/Homer	1.00E-04	-11.337781	347	7.56%	2479.3	6.02%
27	p63(p53)/Keratinocyte-p63-ChIP-Seq(GSE17611)/Homer	1.00E-15	-35.032239	561	12.22%	3587.1	8.70%
	p53(p53)/Saos-p53-ChIP-Seq(GSE15780)/Homer	1.00E-13	-31.497284	150	3.27%	681.8	1.65%
	p53(p53)/Saos-p53-ChIP-Seq/Homer	1.00E-13	-31.497284	150	3.27%	681.8	1.65%
	p73(p53)/Trachea-p73-ChIP-Seq(PRJNA310161)/Homer	1.00E-13	-31.479788	101	2.20%	385.6	0.94%
28	ANAC094(NAC)/col-ANAC094-DAP-Seq(GSE60143)/Homer	1.00E-15	-34.560091	773	16.84%	5259.3	12.76%
	ANAC042(NAC)/col-ANAC042-DAP-Seq(GSE60143)/Homer	1.00E-13	-32.122544	1343	29.27%	10022.2	24.32%
29	CRE(bZIP)/Promoter/Homer	1.00E-15	-35.09781	428	9.33%	2580	6.26%
	TGA6(bZIP)/colamp-TGA6-DAP-Seq(GSE60143)/Homer	1.00E-14	-32.495891	690	15.04%	4649.4	11.28%
	bZIP50(bZIP)/colamp-bZIP50-DAP-Seq(GSE60143)/Homer	1.00E-12	-28.218652	1044	22.75%	7644.2	18.55%
	TGA5(bZIP)/col-TGA5-DAP-Seq(GSE60143)/Homer	1.00E-11	-25.538945	129	2.81%	602.7	1.46%
	Atf7(bZIP)/3T3L1-Atf7-ChIP-Seq(GSE56872)/Homer	1.00E-11	-27.224544	554	12.07%	3702	8.98%
	JunD(bZIP)/K562-JunD-ChIP-Seq/Homer	1.00E-11	-25.347515	140	3.05%	675.2	1.64%
	Atf1(bZIP)/K562-ATF1-ChIP-Seq(GSE31477)/Homer	1.00E-11	-25.58905	718	15.65%	5050.3	12.25%
	TGA3(bZIP)/colamp-TGA3-DAP-Seq(GSE60143)/Homer	1.00E-10	-24.103941	95	2.07%	402.3	0.98%
	TGA4(bZIP)/colamp-TGA4-DAP-Seq(GSE60143)/Homer	1.00E-10	-25.020295	317	6.91%	1930.8	4.68%
	c-Jun-CRE(bZIP)/K562-cJun-ChIP-Seq(GSE31477)/Homer	1.00E-10	-25.29452	362	7.89%	2262.5	5.49%
	TGA1(bZIP)/colamp-TGA1-DAP-Seq(GSE60143)/Homer	1.00E-09	-22.20143	452	9.85%	3020	7.33%
	Atf2(bZIP)/3T3L1-Atf2-ChIP-Seq(GSE56872)/Homer	1.00E-07	-17.549907	393	8.56%	2671.4	6.48%
	FEA4(bZIP)/Corn-FEA4-ChIP-Seq(GSE61954)/Homer	1.00E-06	-14.094721	1225	26.69%	9737.5	23.63%
30	bHLHE41(bHLH)/proB-Bhlhe41-ChIP-Seq(GSE93764)/Homer	1.00E-14	-34.042381	1899	41.38%	14729.2	35.74%
	bHLH74(bHLH)/col-bHLH74-DAP-Seq(GSE60143)/Homer	1.00E-11	-26.10869	529	11.53%	3531.5	8.57%
	USF1(bHLH)/GM12878-Usf1-ChIP-Seq(GSE32465)/Homer	1.00E-11	-27.272968	800	17.43%	5667.9	13.75%

TFE3(bHLH)/MEF-TFE3-ChIP-Seq(GSE75757)/Homer	1.00E-09	-22.924755	133	2.90%	654.8	1.59%
Usf2(bHLH)/C2C12-Usf2-ChIP-Seq(GSE36030)/Homer	1.00E-08	-18.8026	449	9.78%	3081.4	7.48%
BIM3(bHLH)/col-BIM3-DAP-Seq(GSE60143)/Homer	1.00E-08	-19.389342	210	4.58%	1238.2	3.00%
E-box(bHLH)/Promoter/Homer	1.00E-08	-19.839223	193	4.21%	1108.1	2.69%
bHLHE40(bHLH)/HepG2-BHLHE40-ChIP-Seq(GSE31477)/Homer	1.00E-08	-20.203719	570	12.42%	4009.1	9.73%
Cbf1(bHLH)/Yeast-Cbf1-ChIP-Seq(GSE29506)/Homer	1.00E-08	-18.90347	415	9.04%	2810.1	6.82%
BIM1(bHLH)/colamp-BIM1-DAP-Seq(GSE60143)/Homer	1.00E-06	-14.901894	185	4.03%	1131.2	2.74%
SPCH(bHLH)/Seedling-SPCH-ChIP-Seq(GSE57497)/Homer	1.00E-06	-15.010235	1334	29.07%	10631.4	25.80%
NPAS2(bHLH)/Liver-NPAS2-ChIP-Seq(GSE39860)/Homer	1.00E-05	-12.530376	1481	32.27%	12043.2	29.22%
BIM2(bHLH)/col-BIM2-DAP-Seq(GSE60143)/Homer	1.00E-05	-12.202042	993	21.64%	7842.9	19.03%
MITF(bHLH)/MastCells-MITF-ChIP-Seq(GSE48085)/Homer	1.00E-04	-10.869323	1078	23.49%	8643.8	20.97%
