# Supplementary material for: Subtype heterogeneity and epigenetic convergence in neuroendocrine prostate cancer
Source: Nat Commun. 2021 Oct 1;12:5775. doi: 10.1038/s41467-021-26042-z (PMC8486778; doi:10.1038/s41467-021-26042-z)
Supplement: Supplementary file 4 — Supplementary Data 2 [file 41467_2021_26042_MOESM4_ESM.pdf]

Supplemental Table 5. Nearest gene to the SE

LuCaP 93	LuCaP 145_1	LuCaP 145_2	LuCaP 49	LuCaP 173.1	EF-1
MIR10A	FLJ35776	HOXB3	HOXB3	NFAM1	UNCX
SFTA3	HOXB3	ATP8A2	PTMA	CUEDC1	LOC400931
LOC400931	ELF3	FLJ35776	TRIT1	CABP7	PPIF
KCNK9	LOC100130331	ELF3	CASZ1	LOC400931	C10orf91
ELF3	GALNT2	ID2	LOC400931	KLHL29	ENGASE
C14orf4	LPCAT1	TPD52	FAM125B	TP73	SLC12A7
LPCAT1	NEK2	NDRG1	CALML3	KIAA0182	C14orf4
KIAA0182	KCNK9	FAM125B	NFIB	EDN2	C14orf4
NFIB	CEACAM6	CAPZB	PWWP2B	LOC441666	C9orf50
RPTOR	LOC730227	CUEDC1	HOXD9	FOXA1	AHDC1
TEAD1	DLGAP1	GPC6	SFTA3	TNRC18	TNRC18
FLRT1	MGST2	CBFA2T2	PITPNM2	MGST2	CASZ1
CXCR7	CUEDC1	DLGAP1	LMO3	LOC400548	LYL1
GAS6	TPD52	GALNT2	CBFA2T2	ELFN2	FAM167A
CDKAL1	IRF2BP2	SNORA14B	CDH5	KIAA0182	CAMTA1
FAM174B	WISP1	POU2AF1	DMAP1	LOC100130298	CHMP6
MBIP	MICALCL	HES1	SLC36A4	GINS2	PITPNM2
VAV2	IRS2	LPIN1	CUEDC1	UBE2MP1	SREBF1
CASZ1	GPC6	MGLL	CBX4	LRRFIP1	SUN1
LMO3	EHF	FLJ35776	C1orf127	BEGAIN	NCRNA00163
CCDC33	ZNF608	NCRNA00184	CAMK1D	ABR	KRT42P
FOXA1	MALAT1	COTL1	FAM53B	LHX3	CABP7
KIAA0247	ZMYND8	RREB1	LFNG	PIPOX	C16orf78
PITPNM2	GRB7	BCL2L1	C14orf4	TOB1	CTDSP2
FLJ35776	SNORA14B	SAFB2	TMEM8C	NGFR	GAA
NCRNA00051	TMEM181	TRIT1	LOC100130331	FOXG1	AGPAT3
CUEDC1		LOC400931	BMF	NCOR2	RPL38
SIPA1L3	HS3ST1	LOC100131496	ELF3	UNCX	DAB2IP
MALAT1	NOL4	CCDC130	KIF19	CYTH4	C14orf180
CCDC88C	COTL1	SLC36A4	MALAT1	CWH43	PCP4
CAPZB	RHOH	LOC100131060	LOC100131496	FLRT1	RTDR1
SIK1	PSMG4	ETV6	EHF	SPDEF	CRIP2
CXCR7	NCRNA00184	NOL4	SIL1	ANKRD26P1	PLCH2
TSNARE1	RBM47	ZNF322A	KIAA0182	SRCIN1	ANK1
EDN2	NOL11	GPC6	IRF2BP2	LOC400548	CBX4
WNK2	SAAL1	PRMT8	LOC100271832	C14orf70	RBFOX3
LOC100271832	MIR21	SLC6A6	ST14	C14orf4	LGALS3
LOC730227	FAM125B	MALAT1	MIR3186	FAM101A	LOC100310782
SYT7	RREB1	LFNG	CACNA1A	MIR148A	MIR548W
CLDN4	SIX1	DSCAML1	KIAA0182	MIR1286	LOC100129534
C10orf91	NACAP1	SIX1	ASCL1	MCHR1	ESPNP
LOC100129066	ANKRD46	HOXB8	RPTOR	GRIK4	ATOH8
RASA3	RFTN1	EHF	SAFB2	CCDC85C	SMCR5
PTMA	DLGAP1	GFRA4	C2orf55	MIR3183	SLC5A10
CBFA2T2	FLJ35776	CASZ1	C20orf144	CRIP2	ENPP7
KIFC3	GPC6	SYT7	EDN2	LOC284648	KLF13
CCDC85C	TEAD1	LOC100422737	DBH	MIR3199-1	ACAP3
CDKN1B	DSP	RAI14	UTRN	MIR634	UNCX
LIFR	ATP8A2	C14orf4	DSP	MCART1	MIR4297
NCS1	MGLL	SAAL1	CEACAM6	CLSTN1	CCR7
DLGAP1	LPIN1	LOC100130275	NEURL	LOC100499467	DNAJB12
ASCL1	9-Sep	ABR	FAM102A	MIR4297	LOC100129034
MSI2	C14orf4	MICALCL	NFIC	KDM4B	EBF3
LOC400548	CCDC130	INSR	PTP4A3	WDR25	SRRM3
WASF2	RPTOR	TNRC18	MYH9	LOC100130298	OTOF
CRIP2	CAPN2	CEACAM6	REPIN1	C16orf78	MALAT1
CMBL	SEMA4B	SLC37A1	SREBF1	FAM83F	ABLIM2
TGIF1	LFNG	ARFGAP2	CLDN3	UNCX	MSI2
ASB9P1	GPC6	KRT7	GEMIN7	NCRNA00207	MLN
RREB1	IRS2	HNRNPL	LOC100289410	RTDR1	GRM4
GTF2I	ASB9P1	C3orf21	KRT8	SYT13	SRP9
FAM178B	C9orf106	VPS37C	SETD1B	LRRTM2	MIR3689B
LOC730668	IP6K3	DDC	MIR375	TPTE	PCMTD2
LHX3	CCRN4L	LOC730227	C9orf106	NCRNA00184	RXRA
SEMA4B	TNRC18	TGIF1	FLRT1	ADAMTS2	LY6D
TGIF1	TGIF1	MIR612	SEC11C	DPF1	CEP164
SLC12A7	SATB1	FOXJ3	TNRC18	KIAA0649	C19orf29
SCNN1B	IRX2	LGALS8	LASS4	WASF3	EML1
IP6K3	SFTA3	C18orf1	LRRTM2	CPLX2	ZNF536
FAM129B	CHN2	ADCYAP1	LOC100130331	UNC5B	PVRL1
ZNF750	SIPA1L2	C8orf75	TNS1	MICALL2	FAM83F

CLMN	FAM84B	NEK2	SOX2	SUN1	LOC730227
CALML5	ST3GAL1	NFIC	ZBTB7B	FGFR3	PLEKHA6
LOC100131496	TMEM63A	KIAA0182	HOXA9	PITPNM2	IRF2BP2
ZIC2	PGBD5	KIAA0247	MIR378C	MIR4311	BAG5
MBIP	MIR1287	ZBTB7C	DSCAML1	TIAF1	C16orf78
NACC2	NEAT1	RFTN1	FAM69B	ANKRD30BL	NFAM1
DDX11	HFE2	NUAK2	SH3BP4	CHST8	ZC3H3
MYT1	LOC100499467	SFTA3	LAMA5	GHRHR	ATP11A
KCNIP3	NKAIN2	C1QTNF3	CA10	HAAO	C21orf2
C2orf55	PBX3	RAB40C	DDX11	EGFR	LOC730668
CDKN2C	LOC400931	LOC100271832	MEF2D	C20orf117	DISC1
IER5	MIR30D	ATP2A3	IP6K3	9-Sep	SEPN1
KRT8	SLC6A5	LOC100133612	CLDN4	KCNN3	UNC5B
SOX4	DLGAP1	SOX4	ACTG1	CCDC33	RNF187
LFNG	KRT8	NAV2	PIM3	LOC399744	C14orf180
BCOR	RINL	EPB41L4B	HSD11B2	PPIF	CROCC
MIR326	OR51E2	FBXW4P1	THRA	SMCR5	FLJ43860
RALYL	C12orf42	SH3BP4	KRT7	MRPS23	KCNA10
MYH9	TEAD1	RANBP3	FAM129B	GPR12	MTSS1
CALML3	PHF19	FAM129B	SATB2	TACC2	PTMA
RALYL	IRF2	LOC390858	SMPD3	ELFN1	C20orf135
LOC648987	SPATA13	SMARCA2	NCRNA00051	FAM83G	HGFAC
NFIX	YWHAZ	RPTOR	NTHL1	MYO18B	VAV2
NEAT1	ABR	C2orf57	NUAK2	PQLC2	CUEDC1
AZIN1	RNF126P1	SEC11C	TLE2	MIR152	C14orf132
NUDT14	EPB41L4B	PITPNM2	LOC100128239	SEZ6	KIAA0125
RBM19	LOC648987	SERGEF	FBXW4P1	FAM49B	RFX2
MICALCL	NAV2	C9orf106	DAGLA	TMEM132E	SIK1
ZNF319	STK24	IGFBP2	TMEM181	MIR1301	MRPL37
HSD11B2	SHISA2	ING1	ANK2	CDC42EP2	C21orf33
KIF26A	GP9	ST14	SIK1	LOC645752	SOX4
CD9	LOC145837	LOC100129066	TFF3	ADAMTS2	TOB1
MBIP	LBR	SIPA1L3	TGIF1	SMCR5	CASZ1
FSCN2	NT5M	DNMBP	FOXA2	C14orf4	DRGX
TRIO	INSR	AMACR	PCBP1	IRF2BP2	PPP2R4
EPB41L4B	TMEM181	ID2	PROX1	TBC1D22A	SLC25A29
MIR196B	ID2	DSP	SELPLG	A2LD1	CACNA1I
FOXJ1	OCLN	TEAD1	NAT8L	ANKRD36BP2	ADAMTS2
PLXNA1	CALML5	OR51E2	NEAT1	FAM19A5	KCNT1
9-Sep	CCDC33	FGF9	GP5	SLIT1	GAS6
DSP	PDE4DIP	RAB11FIP1	ALDH1B1	LOC100294362	ARHGEF19
CDKN1A	SNX16	CA10	SOX4	ZNF706	TMEM92
TRIM14	CDKN2C	NUP210	RREB1	KIAA0087	SPEG
TNRC18	SYT7	HOXD8	KCNK3	ACTR3BP2	SH2D6
COPS8	LOC100131060	NFIB	LOC730227	OLFM1	LOC150197
CRB2	NSMAF	BMF	SLC37A1	C17orf54	TSPAN9
ATP2C1	ARHGEF38	INSM1	BRD2	C6orf129	KCTD7
TP73	LOC100130275	MIR1203	ZC3H4	TMEM53	FCN1
SLITRK6	LOC284788	IRS2	LIFR	ABTB1	FAM102A
BRD2	GINS2	DNAJC12	SPTBN5	PBOV1	CAMK2A
AGPAT2	KLF3	MYH9	EFHD2	LOC730668	B4GALNT3
RASD1	PBX1	SH3BP5	EDN2	GALNTL4	FLJ45079
ELFN1	INSM1	BCOR	COTL1	NEUROD2	TBC1D16
FAM102A	MAML3	FAM102A	UBTF	ALG2	FOXN3
NPTX1	MIR135B	ATP2C1	HES6	LRP5L	FMOD
CCDC33	ATP11B	MEF2D	ZIC5	TPRG1	IQSEC1
KLF13	SIPA1L2	PPAP2C	HDGF	IGLL1	LOC730441
KIF19	MIR23B	FAM174B	SCNN1A	LHX2	CROCCP2
TNS1	CBFA2T2	ETS2	ABTB1	WASF2	KLHL29
AHRR	MBNL2	FNDC3B	MIR3621	MIR148A	C1orf198
IRF2BP2	SGPL1	MGST2	LOC730668	SCARB1	PSD2
S100A2	RASSF5	TCERG1L	C6orf35	RASD1	TBC1D22A
TMSB4X	FOXA1	CAPN2	NOL4	LOC574538	CBX4
SOX2	NUDT12	LASS4	KLHL29	TMEM115	ALOX15
ARHGEF16	KIAA0182	TM4SF1	PPAP2C	LOC282997	TPPP
LOC284648	NEDD1	SDC1	ARHGEF2	TCP11	LRTM2
ABR	ATP2C1	CCDC40	LPIN1	C14orf23	SYT2
KLF13	ACP6	INSM1	C16orf91	MIR378C	MIR657
CEACAM6	COL4A2	LOC643406	MIR3188	C5orf15	OTUB1
POU2AF1	ENPP6	C1QTNF3	MYOF	C7orf41	COL5A1
COTL1	GFRA4	COMP	CCDC88C	BMPER	LOC400238
FOS	SERGEF	CHD6	FOXP4	SREBF1	NOTCH1
ABHD11	IER5	CNKSR3	RAB11A	FAM102A	STRA13

C14orf182	TNFSF8	NT5M	LENG9	TSPAN18	LOC283177
ETV6	PLXNA1	UTRN	IL17REL	EDN2	PHACTR3
SIL1	SOX4	VAV2	NCRNA00245	ZC3H3	RAPGEF1
LENG9	AFAP1	C7orf41	UCP2	AIPL1	PLXNA4
BMF	CLIC1	FAM190B	RANBP3	INSM1	SIM2
TERF1	SLC6A20	LMO3	LOC100129427	REV1	MGAT4A
LOC100272217	PITPNC1	ST18	LOC283335	TDRG1	ATP6V0E2
INSM1	SAT1	KRT8	NCRNA00184	PCSK9	KCNJ18
C14orf132	KIAA1530	KIAA0087	TEAD1	KRT15	MAD1L1
ST18	ID1	BCOR	FOXJ3	KCNK16	C22orf34
PSMB1	DNMBP	MBIP	NEK2	UNC5A	MIR629
FAM53B	ASCL1	KCTD1	C20orf112	LOC100132354	BCL11B
TBCC	ATP13A3	MIR21	LOC100130894	CEBPG	TP73
ISLR2	FOXJ3	SIPA1L3	DLL4	ANGPT1	RHOF
SMAD7	RAB11FIP3	PGBD5	GPR37L1	C9orf50	AFAP1
DUSP16	TRIB1	RET	LOC100128788	SLC35C2	YWHAZ
C1QTNF3	SHISA2	GRB7	GP9	MIR589	LOC400238
HSD17B6	SUMO1P1	CCDC162	HS6ST1	CPLX2	MKI67
HES1	MIR375	NCRNA00184	PRR15L	MIR135B	NEUROD1
BCOR	NR4A2	FAM178B	ACTB	ITPRIP	TRIOBP
NEK6	DCAF4L2	CLDN3	SOX13	NCRNA00051	CCDC33
LOC400238	WEE1	AFAP1	ZMIZ1	NFIB	MAP2K3
GRHPR	S100A11	CDKN2C	BCOR	ADAM6	LSP1
GADD45G	GRHL2	PGM2L1	RUNX1	NAA11	KREMEN1
KIF26A	ZFAT	C2orf55	CAPRIN1	C5orf15	EIF2C2
IMPA2	RALYL	ARHGEF38	ZFHX3	C18orf1	ANK1
LOC284751	LOC390858	EDN2	NR4A2	MED27	LOC440926
MARK2	ABHD11	PCBP1	MIR1227	LOC642236	MICALL2
C14orf181	MYT1	PBOV1	MAML3	OLIG3	AZIN1
NTHL1	SPPL2B	SEMA4B	CRIP2	CCR7	SHANK2
KRT7	C2orf57	CDON	DUSP22	LOC400548	PTP4A3
COMP	IL24	SRP9	HNRNPF	ADARB2	C9orf69
ZMIZ1	B4GALT5	MIR23B	HNRNPU	CHN2	COL9A2
SRSF3	HOXD8	ST6GAL1	SCOC	TBC1D16	CACNA2D2
TPD52	C20orf144	SLC4A11	SPPL2B	MYH9	C14orf70
TSC22D2	FLT1	NCRNA00111	SBNO2	NCOR2	CIC
C1QTNF3	GRHL2	SATB1	CDH1	NRXN1	NHLH1
ADCYAP1	LOC100133612	RASA3	DLEU2	C8orf46	INSM1
YKT6	CACNA1C	TCF12	LOC100129066	TRPM8	CRHR2
LOC100133545	INSM1	ASCL1	KIAA0240	SH3TC1	FLJ43860
FLJ35776	NUAK2	ASB9P1	MED25	C18orf1	MIR1275
C14orf180	ZBTB7B	LOC100133612	CTBP2	FAM174B	ELF3
MIR23B	LOC100133545	LOC283050	PLXNA2	EBF3	ELFN2
UTS2R	SCOC	LOC100130331	FLT1	KLHL29	ZMIZ1
KCNK5	PGS1	HS3ST1	CXXC5	ADSSL1	ARL16
C2orf85	MSI2	PLXND1	SH3TC1	ROCK1	CTTN
NCRNA00051	CDH5	SIK1	LOC283663	TARBP1	PSMB1
CHSY1	COL21A1	FOXA1	MIR937	GPR132	C14orf162
GLRX3	GPC6	AACS	BCL2L1	IGSF21	DMRTB1
GPRC5C	BCOR	TNFSF8	HOOK2	ASB9P1	TIMP2
CTBP2	TMEM30B	KIAA0182	SMARCA2	HIC1	TMEM121
COPS8	KIAA0240	TOB1	ACSM2B	LOC100129066	NAV1
DACH1	SLC37A1	LOC338799	MGST2	CNTN2	LOC100132707
SETD1B	BMF	LRIG1	LOC100130298	FBLN1	TPPP
HS6ST1	SCNN1B	FOXP4	SNCAIP	BEGAIN	B3GNT7
MACF1	PPAP2C	C11orf92	MIR326	ROR2	LHX3
SOX4	LASS4	RALYL	FOXA1	ANKRD46	ZFAT-AS1
LOC221122	ADCY9	LOC283663	SPEN	FAM125B	MYT1
BAIAP2	SEC16B	PDGFRL	PDE4DIP	MYO18B	CELF4
EFHD2	ZC3H4	C1orf127	ID2	TRIM29	KDM6B
MAL	RAB20	PSMG4	C12orf44	SEZ6L	GEMIN7
RET	HIST2H2BE	NFIA	MFSD10	TSPAN9	SDK1
ADAM6	TLE2	FBXO32	GPR84	CDH17	GTF2I
NCRNA00184	IER2	RALYL	SIK1	CAMK2B	C13orf35
PSD4	LOC100129066	MGLL	MDC1	PLXNA1	BCOR
ID4	GPC5	ID4	C21orf29	C14orf70	CORO2A
ProSAPiP1	HOXB8	RASSF5	TUBA1A	IMP5	LOC282997
PRKCD	ATP7B	LOC145837	SNORA14B	SYNPO	INF2
C1orf127	MIR759	TARBP1	GTF2I	VSTM2L	FSCN1
MDC1	RIMS2	MIR135B	BCOR	NEUROD2	CRYBB3
YWHAZ	ANKRD10	RARA	C10orf108	MIR4311	BAT2L1
CBFA2T2	PRAC	EFHD2	ID2	GSDMC	FAM131C
INSL5	FLJ13197	C12orf42	KLF6	TMPRSS15	C14orf180

C16orf81	RAPGEF5	MIR196B	C1orf152	NCRNA00051	TMEM53
BCL2L1	ADCY5	ARL4C	HK1	VAPA	PPP2R2C
PRDM6	SIL1	HOXB1	CEP164	CWH43	SLC19A1
C14orf180	PTP4A3	TMSB4X	INSM1	CA8	TBC1D22A
BCR	TFCP2L1	DMAP1	LOC100131060	MDM1	ADCK1
GRAMD4	BCOR	UNQ6975	C19orf61	MIR4251	KIAA0182
GPR84	BAALC	TBL1XR1	AHDC1	MPRIP	TESC
AMOTL2	C12orf42	MYOF	MIR4310	LOC148189	CHST3
NT5M	DUSP6	SYDE2	TEAD1	KLF13	PLXNA4
C12orf44	GABRG3	TEAD1	LIMS1	GPR12	RTN4RL1
KCNK16	PRKAB1	CLDN4	CPA1	MRM1	KCNQ3
ARAP1	CHRNA9	PPP1R3B	RET	LOC100130298	PGBD5
LPIN1	NOS1	LOC100130331	IGFBP5	VWC2	EDN2
ZFHX3	LGALS8	STEAP3	CDC25B	SCGN	NEAT1
ZBTB7C	LOC440926	RHOH	GRP	ECE1	MIR601
9-Sep	SIPA1L2	LOC100133545	SIPA1L3	RNF19A	CXCR7
ISYNA1	ATP1B1	TBC1D8	HNRNPL	KCNMA1	NFE2L1
SPEN	HOXB1	ZBTB7B	NOS3	PRKD1	E2F2
BAI1	CSRNP3	SAT1	ZP1	CDKAL1	MIR589
TERT	LOC399881	CDKN2A	CCDC40	SEC16B	ARHGEF16
LOC283050	TMEM150C	LPCAT1	SGMS1	LRRN2	OLFM1
STEAP3	CRIP2	KCNK9	GALNT2	QSOX2	C4orf23
C1orf89	SLC26A9	RASSF6	MPG	LOC100130275	GPR153
IDH2	ZBTB7C	CLDN10	MAML3	ARL4C	ADCYAP1R1
ZNF217	TRAPPC9	LOC100499467	LRIG1	RAP1GAP	SYPL1
NAV2	CRTC1	TH	TARDBP	CHST15	LMAN1L
MIR3664	PCDH1	FOXJ1	UBOX5	DAND5	KLF9
EZR	CLDN4	MYRIP	MAZ	HK1	MIR152
LYPD1	EEA1	SNORA70E	GNG4	MIR593	C4orf44
TBC1D24	NCRNA00051	DLGAP4	EFR3B	KLK3	TTYH3
MLN	PTMA	RAPGEF5	OPA1	INSM1	MAFF
9-Sep	FOXP2	TSPAN14	MIR3605	HOXA7	MIR4253
RARA	ID2	HOXD11	ARAP1	MRPS27	OLIG1
PEX5L	FAM84B		EFCAB2	NRXN1	C1QA
TREM1	INTS7	KIAA0430	POU3F2	MIR770	KIAA0125
C6orf129	FAM135B	SOX13	ANXA2	ITPKB	NAPA
IER2	INPP4B	RFX3	RBM38	NFIB	PRELP
SEPN1	REPIN1	KCNQ1DN	GPR56	ARHGEF3	WNK2
CDON	MACF1	NR4A2	C20orf203	MIPOL1	VGLL4
PRR20E	CHRAC1	LOC283335	INPPL1	CACNA1A	MSI2
KIF26A	FLJ45079	PRAC	SLC6A5	WBSCR17	FAM53B
TPSD1	POU2AF1	YWHAZ	ATP2A3	FLJ13224	9-Sep
LOC100216545	SH3GLB1	CNKSR3	C10orf140	BAG5	CLSTN1
SIX1	CCR8	MIR193B	UNC13D	VASH1	ADAMTS2
ZC3H4	HIST1H2BD	MCHR1	ETV6	ST18	C14orf93
UBTF	TRAK1	KCNK5	HOXC6	ADAMTS2	DAND5
FOXP4	PLXNA2	PHC2	KIFC3	KIAA1614	RBFOX3
MIER1	MIR23B	TERF1	CAPN9	FSCN1	MIR2278
ZC3H3	IMPA1	RTN4RL2	RNF187	ADCYAP1	CDH22
RGS12	SLC30A8	ADCY5	TPD52	CROCC	GRIN2C
B3GNT8	TRIM14	CPLX2	CBX3	LOC284749	ADAM12
PCBP1	FAM174B	ANKRD46	SEMA4C	PLXNA2	BGN
PIGZ	GEMIN7	SDC4	LOC100499467	HTRA1	HOOK2
NUDT16L1	CAPN9	MBNL2	LYL1	SETD1B	USP2
MIR1178	GPRC5C	ZNF706	NUAK1	NAV1	NGFR
PTPRJ	TRAF3IP2	CCRN4L	ADCY5	WBSCR17	COL22A1
ECE1	MYH9	LOC100132354	KDM4B	MAT2B	MIR4269
FAM125B	ANK2	TERF1	MIR21	C7orf71	KLHL29
FLJ45079	TRIT1	ATP8A2	WDR34	BEGAIN	DUSP7
C14orf180	SOX2	TRAK1	REV3L	KCNK9	BEGAIN
FLJ22536	C1D	XBP1	DLL1	MIR2117	RXRA
DEGS2	UTRN	TLR5	SLC25A42	CCDC33	LOC730441
PLEKHG4B	LOC338799	HOOK2	IER2	RFX2	EMP3
LY86-AS1	PCBP1	TRAF3IP2	LOC390858	LOC643955	NCS1
SPPL2B	ERI3	MCART1	TAGLN2	PVRL1	GRAMD4
SLC7A5P2	ERN1	PHLDB1	CCL28	CHRNB4	DAGLA
ASB2	SLC30A8	KLF13	LOC644172	DNAH9	RNU6ATAC
KCTD1	ZNF217	PLXNA1	TRIM8	LBR	NPTX1
KLHL30	PROX1	GEMIN7	HSPB1	GPR12	EFNA3
ZNF775	LNX1	LOC284648	GREB1	HPCAL1	LOC100129066
MIR578	TOB1	RBM47	DNAJB12	CDHR3	DLX4
CBX8	RPTOR	MIER1	FBXO32	CHD9	LRRC47
ESPNP	RNF19A	CCDC88C	TRAF3IP2	CCDC88C	MSI2

PGS1	SNX16	LOC100499467	SRM	DMBT1	ARFGAP3
LGALS3BP	SH3BP5	RALGAPA2	SMYD3	ALOX5AP	MRPS12
PYGB	AJAP1	PLA2G6	C12orf42	TLE6	MYBPH
LRIG1	PITPNM2	IFNGR2	TUBB2B	FAM201A	MICALL2
SH3BGRL3	GTF2I	SCNN1A	CTDSP2	DNAJB12	LOC100130894
TFCP2L1	CCDC88C	TMEM181	ENTPD2	CPA5	TIFAB
LOC401109	ASXL3	LOC100126784	JUN	MIR200B	ZFP36L2
TMEM30B	HIST1H2BC	ADCY9	ABR	SARDH	MIR601
SUMO1P1	EIF2C2	PPP1CB	FKBP2	GTF3C4	INTS1
SNORA14B	FAM178B	RAB20	ACP6	KCNMA1	C20orf20
SLC45A4	MEF2D	COL4A1	FAM174B	LOC648987	B3GALT5
CKB	SLC6A5	EGFR	AZIN1	SCARB2	ARHGAP12
SLC25A47	BRD2	9-Sep	RNF19A	BCOR	GFPT1
C20orf144	GOLGA7B	LOC200261	ZFAT	BAGE4	HHAT
RHBDF1	FAM49B	RCAN1	HOXB8	PTP4A3	KIF19
MIR595	RFX2	RFX2	KLF13	SH2D6	LOC338799
BAT2L1	AMACR	BHLHE40	ZBTB20	KCNJ11	GPRC5C
WEE1	DEPDC6	BRD2	TRIM47	HGS	NEK6
C10orf140	LRP11	ID1	APOLD1	PRSS55	C15orf50
CLIC1	GTF3A	PBX1	CDKN2C	ITPKB	VPS28
SDC1	NTHL1	IER2	MIR3935	PLXNA2	AKR7A2P1
PROX1	FOXB1	CALML5	SH2D6	FAM53B	NANOS3
MAPK13	RUNX1	TGIF1	RCOR2	LIMS1	RPLP0P2
HES6	RGS12	APOLD1	PLEKHH3	GRIN2C	KCNH6
MIR193A	LRRC43	UNQ6975	TSNARE1	LOC100499467	NAPG
ST18	ATP2A3	TBCC	LOC400548	ITGA3	THRA
HSPB1	MYRIP	FOXN3	CACNA1C	C18orf1	WDR25
CHN2	GPR37L1	TERF1	CROCC	EMX2OS	TMEM200C
RPS24	LAPTM4B	CD9	ATP1B1	KCNH2	TRIM62
ATXN7L1	DUSP10	SPEN	DUSP5P	SCOC	STX16
TMEM52	RAB4A	LIMD1	PCDH1	ZEB2	SLC9A3R1
CACNA1A	LRIG1	RASA2	TUBB2C	KLF13	HVCN1
MIR1471	CPA1	GRHL2	SLC29A4	C16orf78	PACSIN2
VAMP3	SLC7A11	ASXL3	TOX	FAM109A	CSNK1D
FAM100A	CNKSR3	FGF9	HOXC9	KCNS2	PCBP3
AFAP1	RMST	MTSS1	PBX1	LMO1	BCR
NKPD1	NFIB	ATP13A3	ST18	OR11H12	QSOX2
RUNX1	CASZ1	CLIC6	LRRC43	C22orf34	ACTL7B
HOXB1	KLHDC4	KCNQ1OT1	UNC5A	GLOD4	BCL11A
MIR1275	C12orf66	TPD52	VAV2	KRT8	FLNC
LOC388630	MIR23B	C20orf144	CCRN4L	PRSS55	FGD2
PTP4A3	ZNF710	MIR1287	STOML1	PTMA	ATP1A1
PLAC2	KALRN	SOX2	CXXC4	TLE2	MIR4296
SCOC	ABCC4	FOXA2	CDON	TSPAN9	IER2
PPIF	VAPA	SLC26A9	H3F3B	LOC100129534	LHX3
CLPTM1L	CD9	NACAP1	NCS1	CORO2B	RPTOR
CCRN4L	VPS37C	RUNX1	FGF9	TPTE	JMJD4
MIR135B	MIR23B	NEDD1	SPDEF	MYLK	FZD6
HIST1H2BD	TPD52	CPA1	ATP2C1	TMEM200C	TPCN1
CPA1	FUZ	NEURL1B	TRIB1	TSHR	ITPKB
NCOR2	NHEG1	TMEM184B	HES1	NEUROD4	LHX1
LOC646903	MARK1	MKNK2	TNFRSF13C	MICALCL	TSSC1
INSR	ZNF486	MTUS1	SDC4	LOC100130581	MIR3201
C6orf195	MKL2	GBAS	MCL1	UNC5A	MGMT
CHMP6	COL22A1	UBOX5	ANP32B	TSPAN18	NAV1
NCRNA00051	C1orf127	ITGB8	MUC1	MIR4311	SARDH
CROCC	MIR326	STK24	TFEB	VAV2	USP12
C17orf70	ZBTB20	RNF126P1	WWC1	NRP2	STH
VPS37C	C10orf40	DUSP6	VPS37C	FAM174B	PXN
UMODL1	ETV1	GTF3A	FLJ45079	ENPP7	KCNMA1
MARCKS	KIAA0430	REPIN1	MLLT6	HPCA	LOC154449
ETV6	DPF1	CYB5A	RRBP1	MIR601	NPTX1
CHMP6	RIT2	CAPN9	MGAT4A	GUSBP4	KCNK9
MIR551A	ESRRG	GOLGA7B	HIST2H2BE	EMP3	LOC100131199
NPHP4	ARFGAP2	PHF19	SATB1	DAGLA	ARHGAP23
HIST2H2BE	LMO3	MIR1178	TMEM209	GIT1	MEX3A
MEF2D	PIM3	NWD1	C6orf35	RAI1	NRP2
TOB1	HES1	TCTEX1D1	TBC1D8	FLJ42392	PRSS55
NDUFA4L2	SEMA5B	DDX11	NFIA	EFNA5	GMPPA
TOX	TRPM8	DLG5	LOC400940	SYNJ2	GLOD4
MIR200C	EDN2	GPC6	STX16	NEURL	MCFD2
MIR4251	ARL4C	MAML3	ID2	FAM129B	MIR3199-1
OCLN	XKR9	EPCAM	CTDSP1	ROR2	C12orf44

ARID2	FAM102A	RIMS2	LOC100271722	NCAM1	SLC37A1
SMUG1	ATP8A2	KIF5C	BCAT2	TSPAN15	LGALS3BP
FAM84B	PCGF2	TSPAN11	CDKAL1	TMEM121	PMS2P3
CMIP	XBP1	FURIN	PHLDB1	FRG2C	UNCX
EMP3	MIR193B	TM4SF18	UNQ6975	KLF13	C21orf125
TPCN1	LPIN2	SAMD13	WHSC1	CHST3	SLA
C20orf112	DPYSL3	HK1	KCNA5	IER5	PIAS4
CDC25B	BCL2L14	FLJ13197	MICALCL	BMF	BCL7A
LIPC	ENAM	PROX1	FAM178B	NPTXR	NEURL1B
PCDH1	SRP9	SYN2	GRHL2	RAMP1	FAM38B
SLC46A1	CPD	NEURL	ARID1B	LOC100133985	LRRN2
C17orf62	PBOV1	ADM	KLF10	DNMT3A	CERK
NOTCH1	NCRNA00295	CEACAM3	MIR548T	NSMAF	MIR183
LOC100126784	FABP7	UBC	ENO3	C7orf16	PTK7
TFCP2L1	PLAC2	COBL	TSC22D1	CCRN4L	CDKN2C
LOC100130581	GABRG3	C8orf86	GRIA1	TRIM62	WSB1
PGF	TOX	EDN2	LAG3	CSNK1D	ISLR
PPP1R10	RAB20	FAM84A	FOSL2	MIR147	EFCAB4B
DMRTA2	HOOK2	RERE	FBLN7	ARHGAP12	RBM38
VASN	NR0B1	C14orf132	RASA3	PODXL	ANGPTL2
JUN	MYOF	PIK3R4	LOC149950	APOL4	KCNAB2
TACC2	CBFA2T2	TLE2	CLIC1	SPSB4	ZAP70
LOC100133612	GLUL	SCN3A	ZC3H7A	SLC6A9	NCRNA00051
STIP1	LOC284648	ICA1	SIL1	TNP1	TESC
PTCH1	PBX3	CCDC6	TACC2	TMEM121	VAMP3
BCL2L14	PAX9	PSD3	NUDT14	CALML3	RNF213
LITAF	RUNX1	CDH5	ZBTB7C	BCL11B	TMEM121
RIMS2	GLT1D1	SAMD13	KIF26B	GINS2	FOXD2
ING1	TERF1	TRIB1	UBE2I	MIR4319	DUSP1
FAM134B	MSI2	ZNF704	NR2F1	ZNRF2	MAP3K14
MIR4290	RASSF10	SEC14L1	ARID2	RPS24	CXXC5
TMEM115	KCNK9	ATP9A	SETD5	BOD1	CASZ1
LY86-AS1	SUMO1P1	CRIP2	KCNQ1DN	RGMA	C14orf49
C9orf129	C1orf100	LRRC43	NRXN1	PPP1R13B	MGC23270
9-Sep	CCL28	GSK3B	RAB12	NCRNA00221	RPS10P7
TRAPPC9	PELO	INPP4B	PHF2	FRG1B	CORO1C
MRPS23	MRPS28	SMPD3	LMNA	IGFBPL1	HS1BP3
VPS37B	FBXO32	RNF183	RFX3	FAM13C	CAMKK1
WDR34	CHGA	FAM46A	CDC34	VEZF1	MIR4284
MAML3	GXYLT1	RAI1	CAPN2	KCNQ1DN	WBSCR17
LOC283070	SYTL3	ST18	B3GNT5	RALGAPA2	FOXN4
RERE	INSC	ANKRD10	MGC16025	CPLX3	DLL4
BEGAIN	NCR2	SMOC2	NDUFS8	SLC2A9	RALGDS
CAMK1D	MIR592	GPR37L1	B3GNT8	NISCH	CAPN9
LOC282997	HIST1H2AK	C10orf91	CCDC33	ENPP7	KCNJ4
SHANK2	SDC1	UAP1	ASXL3	C14orf23	KLF13
KLRK1	ANKRD46	HDGF	TRAF2	EGFL7	MLLT6
C1orf95	PIK3R4	ZC3H4	ZBTB7A	SLC12A7	PIK3C2B
HIST1H2AC	TOX	CBX8	DNASE1L2	HES1	CROCCP3
ATP1B1	BCL2	B3GNT2	CHAD	CECR1	SPSB4
RAP1GAP2	LOC283663	B3GNT8	RUNX1	KCNK9	GNAL
TMEM53	AZIN1	IL24	WEE1	INSM1	PTPRF
GOLGA7B	MLL5	SCARB1	ASB9P1	DPF3	SEMA3F
RAD23B	CD44	CLIC1	CHD4	DCAKD	SNORA11B
SLCO3A1	CDC25B	MACC1	RPL10A	GTF3A	C18orf1
RUNX1T1	REEP3	TBL1XR1	ARFGAP2	TSPAN5	ACE
NET1	CDKN1B	LY86-AS1	C2orf85	SEMA5B	GPR132
KIAA0649	ATP11B	CDKN1A	C11orf63	PSD2	BAHCC1
FAM84B	IRF2	ZNF704	SPIRE2	NINJ2	LIMK1
MIR1203	MIR3170	GSN	IGFBP5	ZMYND8	BCOR
AQP9	FAM46A	C12orf42	NBPF14	KIAA0802	HEXIM1
TXNIP	ST18	MDC1	TMEM61	NEUROD4	ASB2
AMACR	PTMA	MARK1	EBLN2	TMEM196	LOC642852
C3orf58	ZFHX3	IP6K3	NACC2	MIR216B	ZNF423
MRPS10	IGFBP2	OR1M1	AFAP1	KRT42P	SH3TC1
GRHL2	COX6C	ATP7B	ESPNP	APOBEC3A	SORCS2
GAA	SIPA1L2	GP9	RIT2	RHBDL3	EBF1
MIR620	FBXW7	JUN	BCL9L	LPHN2	HSPA9
CNGB1	FABP5	WEE1	VSTM2B	KLF13	PGS1
NUAK2	FAM135B	PGPEP1L	PFKP	ZFPM2	9-Sep
EGFL7	PLA2G6	ABTB2	MARCKS	FAM163B	HOXD11
ATP2A3	CHMP6	KIAA1324	TLE6	LOC221122	10-Mar
ZBTB7B	MIR601	ZNF219	HIST1H3B	FLJ42392	LOC574538

BAG5	NRP2	CTDSPL	BEGAIN	ZFAT-AS1	RAP1GAP2
SFRP1	PROM1	NCOR2	IDH2	C10orf108	CLDN5
NDUFS8	AACS	ARHGEF2	SAMD13	RASEF	RHBDL3
MCHR1	MGC87042	CA4	UBC	PPM1F	SLC25A1
FAM83H	EPHX1	ATP5O	AHNAK	PLXNB2	FNDC5
LOC400548	SYCE2	CSRP1	MIR23B	SGMS1	TTC15
ODZ3	MACC1	C20orf112	CCDC12	TUBA1A	NCOR2
RERGL	EXT1	ARSJ	KCNK9	TP53I11	KIAA0649
SCNN1A	ERRFI1	SLC30A8	NAV2	TMEM209	NDUFB7
GRIK3	SCG2	C9orf152	NUDT16L1	FAM105A	RPL38
GLIS2	SPEN	LRP4	ACSF3	NHLH1	MIR3183
PRKAR1B	XYLT1	PPAP2B	NPHP4	PEPD	ALOX5AP
VAMP3	NRXN1	STAG1	RERE	C9orf106	ANKRD30BL
PPP1R13B	OSTCL	WIPI1	SLC6A6	HIVEP3	PIGT
RAB40C	TLE6	SHISA2	TFCP2L1	EPHA4	MIR656
CAPN9	SLC38A11	NHEG1	MIDN	C11orf92	ZC3H12A
RINL	TACR2	AMIGO2	PCGF2	PRDM11	CBX8
MIR4290	CHD6	LOC442421	B3GALT5	SLC17A6	CLPTM1L
TSPAN14	CTDSP2	B4GALT1	FLJ35776	GPR153	PHACTR3
HOOK2	C13orf29	LOC440926	HSPA8	TRAF3IP2	DENND3
ZBTB7A	FOXP2	PITRM1	LZTS2	DSCAML1	FOXA1
MGC16025	BCR	RIMBP2	PKD1	BICD1	LOC100132356
ACSBG1	LIMCH1	LOC100128023	FLJ35776	IGFBP5	TSPAN11
AES	ZBTB7C	GADD45A	ERBB2	CHN2	KCNMA1
ZIC5	SLC7A5P2	GREB1	TSPAN11	DIRAS3	FKBP5
SCNN1G	SYNJ2	CBX3	SCGN	SOX9	FZD1
LRRC33	SEPW1	SLC1A3	TERT	TNFAIP8	CRABP1
PCGF2	SPIRE2	MCF2L	CMBL	ADCK1	SLC9A1
DEGS2	RNF183	MIR326	RGS12	FAM19A5	TBCC
CACNA2D2	TLE3	SCOC	HCFC1	SIPA1L3	KCNC4
MIR3188	TBL1XR1	PRKACB	FLJ13197	ZNF608	AQP12A
LY6H	C14orf182	BCL2	VPS28	MIDN	KLF13
H1FX	CD55	GLTSCR1	NEK2	PLEKHH3	UNC5B
SKIL	EYA2	PDCD6IP	ESRRG	TOX	PHYHIP
GP9	SCN3A	PYGB	CA5A	LOC400238	IGLL1
SLC37A1	HOXB13	9-Sep	ANO9	RASGRP1	GPR3
GRB7	RASD1	PREP	DLGAP4	CDH4	CDC42EP4
SKAP1	FAM155A	NCRNA00189	LOC100130298	TXN2	STOML1
CEBPD	EPCAM	NCRNA00152	PSIMCT-1	NXPH3	METTL2B
MLLT6	TOB1	ANK2	LOC100133985	LOC145837	C2orf57
ENPP7	SGMS1	PIGZ	ZFP36	MAML3	APCDD1
HNRNPA2B1	LY86-AS1	KLHL38	SYCE2	TSSC1	KIAA0802
LOC100507217	GPC6	CDC25B	MIR99B	PLCG2	PCDHGC5
HAAO	TMEM80	VPS28	TNKS1BP1	GNB1L	C20orf117
MEG3	NCRNA00295	ZFHX3	NCRNA00051	EFNA5	GRASP
SH3BP2	NCRNA00152	BCAM	NCOR2	KIAA0125	LHX2
PLEKHH3	FABP12	TMEM63A	MIR200C	RUFY1	TACR2
LOC100129637	HES1	C6orf35	SEC14L1	C13orf26	KCNN3
LOC283050	NKX2-2	SYDE2	MIR4291	KCTD1	HILS1
NRCAM	C2orf55	PTPRF	MIR1301	AGXT2L2	FOS
CEP72	NAV2	ZBTB7C	CFL1	C17orf69	TBC1D22A
LOC440926	LIMS1	NCRNA00295	BAT2L1	CEP250	TBCD
SLC13A2	RGS17	C21orf125	LOC100129716	FLJ43860	MYL7
CABLES1	INSIG1	KCNQ1	LOC442421	CSF3R	C20orf106
SP9	LOC100129716	TSGA14	HNRNPUL1	LYL1	MYO18B
EPCAM	SRSF5	VAV2	MLN	LOC100130000	SFN
ST6GALNAC4	MIR378C	C10orf40	TUBA1C	DEGS1	PGF
C20orf203	YPEL2	TXNIP	PIGZ	R3HCC1	MSI2
FLJ44054	GRHL2	SCIN	CTRL	TRIM29	IP6K3
NFIC	PROX1	MIR375	KCTD16	LMO1	B3GAT1
UBC	TCF12	SNTG2	C17orf54	FAM178B	RSPH1
P4HA3	RALYL	DLL4	FAM101A	PBX3	ZNF750
SHANK2	C1orf55	EPAS1	CCDC144NL	KREMEN1	TMEM130
LOC284009	FAM5B	DPYSL3	TXNIP	MGC12916	MBP
TRAPPC9	SIPA1L2	ZNF710	LOC100130581	ZNF536	DKFZp566F0947
PRDM11	ANP32A	C9orf69	LOC100216545	FAM135B	PSMA6
SOX4	SMARCA4	CA5A	KLHL38	STEAP3	C12orf34
PHC2	MAML3	GPC3	AGK	CRYBB3	NKD2
SCRIB	SIK1	VANGL1	C14orf49	C9orf150	TMCC2
PRMT8	SEC16B	FKBP2	LDB1	CRISPLD2	LOC642852
GPR56	CAPZB	RAB3B	PSMG4	TESC	SGSM3
HIST1H3D	PLXND1	TLE6	MIR3165	CAMKK1	C10orf108
TNS3	10-Mar	MBIP	CROCCP3	CIC	PCBP3

CHD3	JUN	CELSR2	MIR645	SORL1	REM2
NACAP1	VPS54	GPC6	ENPP6	GRHL2	ESRRB
ESRP1	C8orf75	ATP1B1	CHRAC1	LSAMP	NET1
C11orf92	RAB4A	SYCE2	MYT1L	KIAA0649	KRT80
PPAP2C	PBX3	LOC648987	C9orf106	POU2AF1	BEGAIN
MGLL	RPTOR	DSCR3	WNT11	TMEM106A	LAPTM5
PTPRF	DDX11	IRX2	KCNH6	HEY2	C14orf181
LOC388630	C5orf39	CCL28	FAM155A	CELF4	FLJ43860
RGS3	BCL2L1	FSCN2	LMNB1	NKX2-2	ADCK2
ZBTB20	CA8	FANK1	LRRFIP1	REEP3	BCR
KDM4B	NDRG1	HOXC6	MARK1	C14orf162	RAP1GAP
C12orf44	UCP2	BFSP1	MKNK2	AGPAT3	KIAA0802
NOMO2	HEXIM2	FUZ	FCRL5	CCDC34	MEF2A
RAB12	SMYD2	ERN1	CASD1	LOC441204	CSRP1
KCNJ11	RASSF6	PRR20E	RAI1	C1QL1	PRLH
MCF2L	DDC	CHD4	ZNF48	FLJ42392	RASD2
NEUROG3	NCRNA00254	SPTBN1	STK32C	TLE3	OTOS
SCHIP1	CA10	SHISA2	CDKN1A	DNAH9	C9orf106
EDEM3	B3GNT8	VEGFA	TOX3	SCHIP1	BCOR
CMTM7	CBX4	RDH10	MIR3180-4	MIR921	EGLN1
QSER1	FOXA2	PDLIM1	AGPAT3	CACNA2D2	CACNG5
LASS4	C1QTNF3	SHB	VAMP3	GRIK3	FAM201A
DNAJB12	GUCY1B2	ACTB	PAM	C20orf112	MED15
CBX4	HHEX	MIR4290	FLJ41200	CD40	CHD5
LRIG3	ID4	TMEM80	CCDC162	FSCN1	FHDC1
CABP7	KLK3	LOC283050	LOC100422737	BCL11B	EEPD1
C14orf43	SYT1	EYA2	XPO1	AHDC1	SDK1
PKD1P1	ST18	PHF8	BIK	SPATA12	TMEM105
ASS1	SPTBN1	ST18	C20orf203	PITRM1	NCRNA00051
HIST1H2BO	LOC100289230	MIR708	LOC388906	WNK2	C1orf187
FLJ13197	C7orf41	TMEM61	FLJ35776	LHFPL2	ADAMTS2
LOC644172	MIR135A2	VPS54	TET1	MYO1B	KCNQ2
MAP6D1	ZNF704	WASF2	INHBB	BFSP2	ZIC5
GPR156	ADCYAP1	SPPL2B	SDC1	HOOK2	SH3TC1
POU3F2	LOC283335	IDH2	NEK6	MSI2	CDC42EP4
SRM	UBC	NCR2	KIAA0087	GAP43	KCNG1
PLEKHA1	BHLHE40	MTSS1	PDE4DIP	ACOT11	ATAD2B
LOC100130275	HSPB1	NFIA	KLK3	CCDC85C	LRP8
PYY2	RAI14	LOC100133545	ITPA	BRSK2	MYH16
KLF10	MUC1	SMC2	S100A2	NFASC	CELF4
DUSP6	LOC283050	FAM163B	LRRC10B	PSD3	LOC100133985
IRX4	CMBL	C20orf197	ETV6	TSHZ3	WBSCR17
DUSP4	DSCAML1	MIR3925	SHANK2	CACNG2	MNT
ENGASE	CTDSP1	TRIM8	KLHL30	KIAA0802	IL6R
DLL1	INPP4B	TBC1D24	RAB40C	CCDC88C	C13orf33
PGBD5	LEFTY1	DUSP4	GLIS2	WBSCR17	PARD3
SSBP3	FLJ22536	C17orf76	UNQ6975	BTBD9	NEUROD2
CHN2	HIST1H3B	MCL1	APP	BNC2	ADCY7
GRHL2	CCDC60	AZIN1	MBIP	UBE2T	TEF
GDF15	SDC4	INSM1	C9orf150	CLDN11	CPLX2
MDK	C14orf132	ATP5O	GET4	GRIK4	RAP1GAP
DAGLA	PDCD6IP	LOC100288748	MIR425	YPEL2	D4S234E
IFT140	MAP7	NCRNA00245	NFIA	NFIX	AKR1B1
KCNK9	BRD7P3	ABHD2	CIC	LOC728758	SUZ12P
GLRX3	LOC100422737	HSPB1	SRCIN1	GJA10	PKP1
HIC2	MICALCL	TOX3	LOC283070	ANGPTL2	BCAN
PRR15L	HIST1H3D	UCP2	C17orf70	MED9	CELSR1
TRAF4	RUNX1T1	WNK2	CTDSP2	LOC100128292	SARDH
RANBP3	CRABP1	NAA40	MRPL38	CKB	NRN1
LDHD	UAP1	AGPAT2	DNMT3A	C12orf65	KCNK9
CEL	AKAP2	MRPL14	SMOC2	GNA12	BID
MAP1LC3A	FAM38B	MGAT4A	WNT11	DLX4	DNAJC5
RASEF	RASEF	BCL2	VEZF1	LHX1	NFASC
MIR3175	LIFR	LOC283143	CABP7	SRSF5	NCRNA00184
GLI2	GPR84	CCDC162	DNPEP	LOC340357	RPH3A
HDGF	CCDC162	IL8	GPR176	LOC730227	ST3GAL1
PLCH2	PFKFB2	ICA1	GTF3A	NEUROD1	NOTCH1
HNRNPF	MIR4290	FLJ22536	TCF12	PMEPA1	ACOT11
NEU3	PTEN	TSPAN1	AP2S1	TYRO3P	FAT2
MED25	ACPP	FZD2	MIR4323	MTSS1L	FLJ45079
AACS	FNDC3B	HSD17B14	FGF9	CALN1	LOC100129066
SEMA3F	C9orf106	MSL2	RMST	NCRNA00051	FAM19A5
ERBB3	ST14	IRX2	SAMD13	PDGFRL	TXN2

NAV1	MCHR1	TREM1	ACTN4	ZDHHC22	MIR4265
ALDH1B1	MIR1243	SLC1A5	NCRNA00152	ANGPTL1	MLLT1
SYCE2	KLF10	REV3L	SLC24A3	MIR3911	PPP1R13B
MIR3165	ZBTB20	YPEL2	ANXA13	SPEG	CHSY1
SH3BP4	KIF26A	NTHL1	CALML3	C6orf123	RIN2
CCDC12	ZBTB7C	LOC100271722	MIR23A	GABRG3	WNT7A
HK1	NDUFB9	CPD	C11orf41	MIR3653	LMO1
STK24	LOC100133985	FBXW11	ST18	STAG3L4	NRN1
SHANK2	ZNF608	ELAVL4	CCDC85C	DKFZP686I15217	TPPP3
LOC723809	PRKCD	ESRRG	LOC100129637	RPRML	ABR
PMS2P4	PCA3	TMEM135	CBWD5	SLIT1	ADCK3
TMEM209	MIR4290	CHD3	MIAT	LOC284009	PRIMA1
CAPN2	NCRNA00245	WWOX	LPGAT1	SLMO1	PLXNA4
FAF1	HIST1H4E	RIPK4	WNK2	TCP11	GLI2
NRXN1	AHRR	TSC22D2	GADD45G	LOC100128023	KCNK3
TK1	CTDSPL	RASEF	PAX5	MRAP2	HAUS7
LMO1	MAD1L1	LYPD6B	SYTL3	LAMA3	SCOC
DLG5	SLC36A4	TMPO	EPB41L4B	C9orf106	SUN1
LOC100129716	PAM	LDHD	PDIA2	LRRN2	RGS3
LOC645332	NUF2	SETD5	ARID1A	MGLL	IGFBPL1
MIR548T	ATP11B	TSPAN9	SLC7A5P2	SYPL1	TFF3
GFRA4	RHOBTB3	ZDHHC2	DIP2A	ZNF536	REV1
TBC1D16	MBIP	ASCL2	PPP1CB	HDAC9	SP3
PBX3	RANBP3	KLHL29	MYT1	KCNC4	TMEM63C
MRPL14	C10orf140	FOS	EPB41L4B	ARHGEF2	TMPRSS4
WDR6	TBC1D8	P2RX4	LRFN4	CPA2	IER5
TENC1	MBNL2	AK2	SYT7	C14orf132	SOCS3
CAMKK1	NPHP4	KCNB2	LRTM2	BRD2	GALNTL4
ZNF703	PDE4D	GNA14	MIR634	RAPGEF5	FAM19A5
NTRK1	SOX1	SMOC2	MIR23A	SPDEF	SORCS2
ELOVL5	LOC388692	CMBL	LPCAT1	KLF13	MIRLET7I
CPM	LOC100288748	CHRAC1	FAM84A	BCL7A	RARA
ERF	SNCAIP	CHAD	LOC283143	EYA2	NCR2
AJAP1	CASZ1	SLC38A1	MIR4296	DMRTB1	NPBWR2
C14orf182	SHISA2	C14orf181	PYGB	SHISA6	TMEM106A
DDX3X	ANXA2	FAM84B	DNMBP	CXXC5	RRBP1
PNRC2	PGPEP1L	GCOM1	KCND3	SEC16B	ARHGEF10L
KLF12	LOC400940	MIR3170	STEAP3	CACNA1G	SRRM3
PTPRJ	KDM6B	RASD1	FOXO3	WSB1	ERCC1
STOX2	LOC100130581	PHF2	PTPRN2	BCOR	MACROD1
TRIB1	CALML3	CXCL17	XBP1	BMPER	NOL11
TFEB	KCNQ1OT1	FLT1	PAM	NTNG2	TMEM132E
LOC100310782	SNTG2	CAMK1D	LHPP	LOC151162	ARL4C
MYOF	CASD1	ATP11B	STK25	GAS7	TRAM2
KIAA2013	FAM129B	PRSS8	ZIC5	DCTD	BEGAIN
TPPP	RAB11FIP1	NCRNA00168	SLC12A7	LTA4H	BCL11B
RASAL1	TUBA1A	GSK3B	PRSS8	GPR37L1	SLC35C2
TRAF3IP2	TMSB4X	VAMP3	TERF1	SALL4	ZEB2
TMPRSS6	ACTB	FBLN7	CHD3	ADCYAP1	ACSBG1
NAP1L4	VAC14	NHS	INPP5B	XPNPEP1	MKNK2
C8orf4	ARID1B	ERBB2	MEPCE	KIF19	LBR
SLC38A1	NMBR	ERRFI1	SIL1	PROP1	BCL11B
TMEM170B	INO80D	CDH1	IGFBP2	NET1	MIR4285
LRRC43	MEF2D	CPA4	PTBP1	PUSL1	LOC100132707
RTP3	STIM2	CSRNP1	ARHGAP22	PPP6R3	TRIP13
**LuCaP 94**	**LuCaP 145_1**	**LuCaP 145_2**	**LuCaP 50**	**LuCaP 173.2**	**EF-2**
MIR10A	FLJ35777	HOXB3	HOXB3	NFAM2	UNCX
SFTA4	HOXB4	ATP8A3	PTMA	CUEDC2	LOC400932
LOC400932	ELF4	FLJ35777	TRIT2	CABP8	PPIF
KCNK10	LOC100130332	ELF4	CASZ2	LOC400932	C10orf92
ELF4	GALNT3	ID3	LOC400932	KLHL30	ENGASE
C14orf5	LPCAT2	TPD53	FAM125B	TP74	SLC12A8
LPCAT2	NEK3	NDRG2	CALML4	KIAA0183	C14orf4
KIAA0183	KCNK10	FAM125B	NFIB	EDN3	C14orf4
NFIB	CEACAM7	CAPZB	PWWP2B	LOC441667	C9orf51
RPTOR	LOC730228	CUEDC2	HOXD10	FOXA2	AHDC2
TEAD2	DLGAP2	GPC7	SFTA4	TNRC18	TNRC18
FLRT2	MGST3	CBFA2T3	PITPNM3	MGST3	CASZ2
CXCR8	CUEDC2	DLGAP2	LMO4	LOC400549	LYL2
GAS7	TPD53	GALNT3	CBFA2T3	ELFN3	FAM167A
CDKAL2	IRF2BP3	SNORA14B	CDH6	KIAA0183	CAMTA2
FAM174B	WISP2	POU2AF2	DMAP2	LOC100130299	CHMP7
MBIP	MICALCL	HES2	SLC36A5	GINS3	PITPNM3

VAV3	IRS3	LPIN2	CUEDC2	UBE2MP2	SREBF2
CASZ2	GPC7	MGLL	CBX5	LRRFIP2	SUN2
LMO4	EHF	FLJ35777	C1orf128	BEGAIN	NCRNA00164
CCDC34	ZNF609	NCRNA00185	CAMK1D	ABR	KRT42P
FOXA2	MALAT2	COTL2	FAM53B	LHX4	CABP8
KIAA0248	ZMYND9	RREB2	LFNG	PIPOX	C16orf79
PITPNM3	GRB8	BCL2L2	C14orf5	TOB2	CTDSP3
FLJ35777	SNORA14B	SAFB3	TMEM8C	NGFR	GAA
NCRNA00052	TMEM182	TRIT2	LOC100130332	FOXG2	AGPAT4
CUEDC2		LOC199862061	BMF	NCOR3	RPL39
SIPA1L4	HS3ST2	LOC299592626	ELF4	UNCX	DAB2IP
MALAT2	NOL5	CCDC131	KIF20	CYTH5	C14orf181
CCDC88C	COTL2	SLC36A5	MALAT2	CWH44	PCP5
CAPZB	RHOH	LOC100131060	LOC100131496	FLRT2	RTDR2
SIK2	PSMG5	ETV7	EHF	SPDEF	CRIP3
CXCR8	NCRNA00185	NOL5	SIL2	ANKRD26P2	PLCH3
TSNARE2	RBM48	ZNF322A	KIAA0183	SRCIN2	ANK2
EDN3	NOL12	GPC7	IRF2BP3	LOC400549	CBX5
WNK3	SAAL2	PRMT9	LOC100271833	C14orf62	RBFOX4
LOC98811378	MIR22	SLC6A7	ST15	C14orf128	LGALS4
LOC198352983	FAM125B	MALAT2	MIR3187	FAM101A	LOC100310783
SYT8	RREB2	LFNG	CACNA1A	MIR148A	MIR548W
CLDN5	SIX2	DSCAML2	KIAA0183	MIR1287	LOC100129535
C10orf92	NACAP2	SIX2	ASCL2	MCHR2	ESPNP
LOC100129067	ANKRD47	HOXB9	RPTOR	GRIK5	ATOH9
RASA4	RFTN2	EHF	SAFB3	CCDC85C	SMCR6
PTMA	DLGAP2	GFRA5	C2orf56	MIR3184	SLC5A11
CBFA2T3	FLJ35777	CASZ2	C20orf145	CRIP3	ENPP8
KIFC4	GPC7	SYT8	EDN3	LOC284649	KLF14
CCDC85C	TEAD2	LOC100422738	DBH	MIR3199-2	ACAP4
CDKN1B	DSP	RAI15	UTRN	MIR635	UNCX
LIFR	ATP8A3	C14orf5	DSP	MCART2	MIR4298
NCS2	MGLL	SAAL2	CEACAM7	CLSTN2	CCR8
DLGAP2	LPIN2	LOC100130276	NEURL	LOC100499468	DNAJB13
ASCL2	10-Sep	ABR	FAM102A	MIR4298	LOC100129035
MSI3	C14orf5	MICALCL	NFIC	KDM4B	EBF4
LOC400549	CCDC131	INSR	PTP4A4	WDR26	SRRM4
WASF3	RPTOR	TNRC19	MYH10	LOC100130299	OTOF
CRIP3	CAPN3	CEACAM7	REPIN2	C16orf79	MALAT2
CMBL	SEMA4B	SLC37A2	SREBF2	FAM83F	ABLIM3
TGIF2	LFNG	ARFGAP3	CLDN4	UNCX	MSI3
ASB9P2	GPC7	KRT8	GEMIN8	NCRNA00208	MLN
RREB2	IRS3	HNRNPL	LOC100289411	RTDR2	GRM5
GTF2I	ASB9P2	C3orf22	KRT9	SYT14	SRP10
FAM178B	C9orf107	VPS37C	SETD1B	LRRTM3	MIR3689B
LOC730669	IP6K4	DDC	MIR376	TPTE	PCMTD3
LHX4	CCRN4L	LOC730228	C9orf107	NCRNA00185	RXRA
SEMA4B	TNRC19	TGIF2	FLRT2	ADAMTS3	LY6D
TGIF1	TGIF1	MIR613	SEC11C	DPF2	CEP165
SLC12A8	SATB2	FOXJ4	TNRC19	KIAA0650	C19orf30
SCNN1B	IRX3	LGALS9	LASS5	WASF4	EML2
IP6K4	SFTA4	C18orf2	LRRTM3	CPLX3	ZNF537
FAM129B	CHN3	ADCYAP2	LOC100130332	UNC5B	PVRL2
ZNF751	SIPA1L3	C8orf76	TNS2	MICALL3	FAM83F
CLMN	FAM84B	NEK3	SOX3	SUN2	LOC730228
CALML6	ST3GAL2	NFIC	ZBTB7B	FGFR4	PLEKHA7
LOC100131497	TMEM63A	KIAA0312	HOXA10	PITPNM3	IRF2BP3
ZIC3	PGBD6	KIAA0377	MIR378C	MIR4312	BAG6
MBIP	MIR1288	ZBTB7C	DSCAML2	TIAF2	C16orf79
NACC3	NEAT2	RFTN2	FAM69B	ANKRD30BL	NFAM2
DDX12	HFE3	NUAK3	SH3BP5	CHST9	ZC3H4
MYT2	LOC100499468	SFTA4	LAMA6	GHRHR	ATP11A
KCNIP4	NKAIN3	C1QTNF4	CA11	HAAO	C21orf3
C2orf56	PBX4	RAB40C	DDX12	EGFR	LOC730669
CDKN2C	LOC400931	LOC100271832	MEF2D	C20orf118	DISC2
IER6	MIR30D	ATP2A4	IP6K4	10-Sep	SEPN2
KRT9	SLC6A6	LOC100133613	CLDN5	KCNN4	UNC5B
SOX5	DLGAP2	SOX5	ACTG2	CCDC34	RNF188
LFNG	KRT9	NAV3	PIM4	LOC399745	C14orf181
BCOR	RINL	EPB41L4B	HSD11B3	PPIF	CROCC
MIR327	OR51E3	FBXW4P2	THRA	SMCR6	FLJ43861
RALYL	C12orf43	SH3BP5	KRT8	MRPS24	KCNA11
MYH10	TEAD2	RANBP4	FAM129B	GPR13	MTSS2

CALML4	PHF20	FAM129B	SATB3	TACC3	PTMA
RALYL	IRF3	LOC390859	SMPD4	ELFN2	C20orf136
LOC648988	SPATA14	SMARCA3	NCRNA00052	FAM83G	HGFAC
NFIX	YWHAZ	RPTOR	NTHL2	MYO18B	VAV3
NEAT2	ABR	C2orf58	NUAK3	PQLC3	CUEDC2
AZIN2	RNF126P2	SEC11C	TLE3	MIR153	C14orf133
NUDT15	EPB41L4B	PITPNM3	LOC100128240	SEZ7	KIAA0126
RBM20	LOC648988	SERGEF	FBXW4P2	FAM49B	RFX3
MICALCL	NAV3	C9orf107	DAGLA	TMEM132E	SIK2
ZNF320	STK25	IGFBP3	TMEM182	MIR1302	MRPL38
HSD11B3	SHISA3	ING2	ANK3	CDC42EP3	C21orf34
KIF26A	GP10	ST15	SIK2	LOC645753	SOX5
CD10	LOC145837	LOC100129066	TFF4	ADAMTS3	TOB2
MBIP	LBR	SIPA1L4	TGIF2	SMCR6	CASZ2
FSCN3	NT5M	DNMBP	FOXA3	C14orf5	DRGX
TRIO	INSR	AMACR	PCBP2	IRF2BP3	PPP2R5
EPB41L4B	TMEM182	ID3	PROX2	TBC1D22A	SLC25A30
MIR196B	ID3	DSP	SELPLG	A2LD2	CACNA1I
FOXJ2	OCLN	TEAD2	NAT8L	ANKRD36BP3	ADAMTS3
PLXNA2	CALML6	OR51E3	NEAT2	FAM19A6	KCNT2
10-Sep	CCDC34	FGF10	GP6	SLIT2	GAS7
DSP	PDE4DIP	RAB11FIP2	ALDH1B2	LOC100294363	ARHGEF20
CDKN1A	SNX17	CA11	SOX5	ZNF707	TMEM93
TRIM15	CDKN2C	NUP211	RREB2	KIAA0088	SPEG
TNRC19	SYT8	HOXD9	KCNK4	ACTR3BP3	SH2D7
COPS9	LOC100131061	NFIB	LOC730228	OLFM2	LOC150198
CRB3	NSMAF	BMF	SLC37A2	C17orf55	TSPAN10
ATP2C2	ARHGEF39	INSM2	BRD3	C6orf130	KCTD8
TP74	LOC99560699	MIR1204	ZC3H5	TMEM54	FCN2
SLITRK7	LOC199406186	IRS3	LIFR	ABTB2	FAM102A
BRD3	GINS3	DNAJC13	SPTBN6	PBOV2	CAMK2A
AGPAT3	KLF4	MYH10	EFHD3	LOC730669	B4GALNT4
RASD2	PBX2	SH3BP6	EDN3	GALNTL5	FLJ45080
ELFN2	INSM2	BCOR	COTL2	NEUROD3	TBC1D17
FAM102A	MAML4	FAM102A	UBTF	ALG3	FOXN4
NPTX2	MIR135B	ATP2C2	HES7	LRP5L	FMOD
CCDC34	ATP11B	MEF2D	ZIC6	TPRG2	IQSEC2
KLF14	SIPA1L3	PPAP2C	HDGF	IGLL2	LOC730442
KIF20	MIR23B	FAM174B	SCNN1A	LHX3	CROCCP3
TNS2	CBFA2T3	ETS3	ABTB2	WASF3	KLHL30
AHRR	MBNL3	FNDC3B	MIR3622	MIR148A	C1orf199
IRF2BP3	SGPL2	MGST3	LOC730669	SCARB2	PSD3
S100A3	RASSF6	TCERG1L	C6orf36	RASD2	TBC1D22A
TMSB4X	FOXA2	CAPN3	NOL5	LOC574539	CBX5
SOX3	NUDT13	LASS5	KLHL30	TMEM116	ALOX16
ARHGEF17	KIAA0183	TM4SF2	PPAP2C	LOC282998	TPPP
LOC284649	NEDD2	SDC2	ARHGEF3	TCP12	LRTM3
ABR	ATP2C2	CCDC41	LPIN2	C14orf24	SYT3
KLF14	ACP7	INSM2	C16orf92	MIR378C	MIR658
CEACAM7	COL4A3	LOC643407	MIR3189	C5orf16	OTUB2
POU2AF2	ENPP7	C1QTNF4	MYOF	C7orf42	COL5A2
COTL2	GFRA5	COMP	CCDC88C	BMPER	LOC400239
FOS	SERGEF	CHD7	FOXP5	SREBF2	NOTCH2
ABHD12	IER6	CNKSR4	RAB11A	FAM102A	STRA14
C14orf183	TNFSF9	NT5M	LENG10	TSPAN19	LOC283178
ETV7	PLXNA2	UTRN	IL17REL	EDN3	PHACTR4
SIL2	SOX5	VAV3	NCRNA00246	ZC3H4	RAPGEF2
LENG10	AFAP2	C7orf42	UCP3	AIPL2	PLXNA5
BMF	CLIC2	FAM190B	RANBP4	INSM2	SIM3
TERF2	SLC6A21	LMO4	LOC99562757	REV2	MGAT4A
LOC100272218	PITPNC2	ST19	LOC199408849	TDRG2	ATP6V0E3
INSM2	SAT2	KRT9	NCRNA00185	PCSK10	KCNJ19
C14orf133	KIAA1530	KIAA0087	TEAD2	KRT16	MAD1L2
ST19	ID2	BCOR	FOXJ4	KCNK17	C22orf35
PSMB2	DNMBP	MBIP	NEK3	UNC5A	MIR630
FAM53B	ASCL2	KCTD2	C20orf113	LOC100132355	BCL11B
TBCC	ATP13A4	MIR22	LOC100130895	CEBPG	TP74
ISLR3	FOXJ4	SIPA1L4	DLL5	ANGPT2	RHOF
SMAD8	RAB11FIP4	PGBD6	GPR37L2	C9orf51	AFAP2
DUSP17	TRIB2	RET	LOC100128789	SLC35C3	YWHAZ
C1QTNF4	SHISA3	GRB8	GP10	MIR590	LOC400239
HSD17B7	SUMO1P2	CCDC163	HS6ST2	CPLX3	MKI68
HES2	MIR376	NCRNA00185	PRR15L	MIR135B	NEUROD2

BCOR	NR4A3	FAM178B	ACTB	ITPRIP	TRIOBP
NEK7	DCAF4L3	CLDN4	SOX14	NCRNA00052	CCDC34
LOC400239	WEE2	AFAP2	ZMIZ2	NFIB	MAP2K4
GRHPR	S100A12	CDKN2C	BCOR	ADAM7	LSP2
GADD45G	GRHL3	PGM2L2	RUNX2	NAA12	KREMEN2
KIF26A	ZFAT	C2orf56	CAPRIN2	C5orf16	EIF2C3
IMPA3	RALYL	ARHGEF39	ZFHX4	C18orf2	ANK2
LOC284751	LOC390858	EDN3	NR4A3	MED28	LOC440927
MARK3	ABHD12	PCBP2	MIR1228	LOC642237	MICALL3
C14orf182	MYT2	PBOV2	MAML4	OLIG4	AZIN2
NTHL2	SPPL2B	SEMA4B	CRIP3	CCR8	SHANK3
KRT8	C2orf58	CDON	DUSP23	LOC400549	PTP4A4
COMP	IL25	SRP10	HNRNPF	ADARB3	C9orf70
ZMIZ2	B4GALT6	MIR23B	HNRNPU	CHN3	COL9A3
SRSF4	HOXD9	ST6GAL2	SCOC	TBC1D17	CACNA2D3
TPD53	C20orf145	SLC4A12	SPPL2B	MYH10	C14orf71
TSC22D3	FLT2	NCRNA00112	SBNO3	NCOR3	CIC
C1QTNF4	GRHL3	SATB2	CDH2	NRXN2	NHLH2
ADCYAP2	LOC100133613	RASA4	DLEU3	C8orf47	INSM2
YKT7	CACNA1C	TCF13	LOC100129067	TRPM9	CRHR3
LOC100133546	INSM2	ASCL2	KIAA0241	SH3TC2	FLJ43861
FLJ35777	NUAK3	ASB9P2	MED26	C18orf2	MIR1276
C14orf181	ZBTB7B	LOC100133612	CTBP3	FAM174B	ELF4
MIR23B	LOC100133545	LOC283050	PLXNA3	EBF4	ELFN3
UTS2R	SCOC	LOC100130331	FLT2	KLHL30	ZMIZ2
KCNK6	PGS2	HS3ST2	CXXC6	ADSSL2	ARL17
C2orf86	MSI3	PLXND2	SH3TC2	ROCK2	CTTN
NCRNA00052	CDH6	SIK2	LOC283664	TARBP2	PSMB2
CHSY2	COL21A2	FOXA2	MIR938	GPR133	C14orf163
GLRX4	GPC7	AACS	BCL2L2	IGSF22	DMRTB2
GPRC5C	BCOR	TNFSF9	HOOK3	ASB9P2	TIMP3
CTBP3	TMEM30B	KIAA0183	SMARCA3	HIC2	TMEM122
COPS9	KIAA0241	TOB2	ACSM2B	LOC100129067	NAV2
DACH2	SLC37A2	LOC338800	MGST3	CNTN3	LOC100132708
SETD1B	BMF	LRIG2	LOC100130299	FBLN2	TPPP
HS6ST2	SCNN1B	FOXP5	SNCAIP	BEGAIN	B3GNT8
MACF2	PPAP2C	C11orf93	MIR327	ROR3	LHX4
SOX5	LASS5	RALYL	FOXA2	ANKRD47	ZFAT-AS2
LOC221123	ADCY10	LOC283664	SPEN	FAM125B	MYT2
BAIAP3	SEC16B	PDGFRL	PDE4DIP	MYO18B	CELF5
EFHD3	ZC3H5	C1orf128	ID3	TRIM30	KDM6B
MAL	RAB21	PSMG5	C12orf45	SEZ6L	GEMIN8
RET	HIST2H2BE	NFIA	MFSD11	TSPAN10	SDK2
ADAM7	TLE3	FBXO33	GPR85	CDH18	GTF2I
NCRNA00185	IER3	RALYL	SIK2	CAMK2B	C13orf36
PSD5	LOC100129067	MGLL	MDC2	PLXNA2	BCOR
ID5	GPC6	ID5	C21orf30	C14orf71	CORO2A
ProSAPiP2	HOXB9	RASSF6	TUBA1A	IMP6	LOC282998
PRKCD	ATP7B	LOC145838	SNORA14B	SYNPO	INF3
C1orf128	MIR760	TARBP2	GTF2I	VSTM2L	FSCN2
MDC2	RIMS3	MIR135B	BCOR	NEUROD3	CRYBB4
YWHAZ	ANKRD11	RARA	C10orf109	MIR4312	BAT2L2
CBFA2T3	PRAC	EFHD3	ID3	GSDMC	FAM131C
INSL6	FLJ13198	C12orf43	KLF7	TMPRSS16	C14orf181
C16orf82	RAPGEF6	MIR196B	C1orf153	NCRNA00052	TMEM54
BCL2L2	ADCY6	ARL4C	HK2	VAPA	PPP2R2C
PRDM7	SIL2	HOXB2	CEP165	CWH44	SLC19A2
C14orf181	PTP4A4	TMSB4X	INSM2	CA9	TBC1D22A
BCR	TFCP2L2	DMAP2	LOC100131061	MDM2	ADCK2
GRAMD5	BCOR	UNQ6976	C19orf62	MIR4252	KIAA0183
GPR85	BAALC	TBL1XR2	AHDC2	MPRIP	TESC
AMOTL3	C12orf43	MYOF	MIR4311	LOC148190	CHST4
NT5M	DUSP7	SYDE3	TEAD2	KLF14	PLXNA5
C12orf45	GABRG4	TEAD2	LIMS2	GPR13	RTN4RL2
KCNK17	PRKAB2	CLDN5	CPA2	MRM2	KCNQ4
ARAP2	CHRNA10	PPP1R3B	RET	LOC100130299	PGBD6
LPIN2	NOS2	LOC100130332	IGFBP6	VWC3	EDN3
ZFHX4	LGALS9	STEAP4	CDC25B	SCGN	NEAT2
ZBTB7C	LOC440927	RHOH	GRP	ECE2	MIR602
10-Sep	SIPA1L3	LOC100133546	SIPA1L4	RNF19A	CXCR8
ISYNA2	ATP1B2	TBC1D9	HNRNPL	KCNMA2	NFE2L2
SPEN	HOXB2	ZBTB7B	NOS4	PRKD2	E2F3
BAI2	CSRNP4	SAT2	ZP2	CDKAL2	MIR590

TERT	LOC399882	CDKN2A	CCDC41	SEC16B	ARHGEF17
LOC283051	TMEM150C	LPCAT2	SGMS2	LRRN3	OLFM2
STEAP4	CRIP3	KCNK10	GALNT3	QSOX3	C4orf24
C1orf90	SLC26A10	RASSF7	MPG	LOC100130276	GPR154
IDH3	ZBTB7C	CLDN11	MAML4	ARL4C	ADCYAP1R2
ZNF218	TRAPPC10	LOC100499468	LRIG2	RAP1GAP	SYPL2
NAV3	CRTC2	TH	TARDBP	CHST16	LMAN1L
MIR3665	PCDH2	FOXJ2	UBOX6	DAND6	KLF10
EZR	CLDN5	MYRIP	MAZ	HK2	MIR153
LYPD2	EEA2	SNORA70E	GNG5	MIR594	C4orf45
TBC1D25	NCRNA00052	DLGAP5	EFR3B	KLK4	TTYH4
MLN	PTMA	RAPGEF6	OPA2	INSM2	MAFF
10-Sep	FOXP3	TSPAN15	MIR3606	HOXA8	MIR4254
RARA	ID3	HOXD12	ARAP2	MRPS28	OLIG2
PEX5L	FAM84B		EFCAB3	NRXN2	C1QA
TREM2	INTS8	KIAA0431	POU3F3	MIR771	KIAA0126
C6orf130	FAM135B	SOX14	ANXA3	ITPKB	NAPA
IER3	INPP4B	RFX4	RBM39	NFIB	PRELP
SEPN2	REPIN2	KCNQ1DN	GPR57	ARHGEF4	WNK3
CDON	MACF2	NR4A3	C20orf204	MIPOL2	VGLL5
PRR20E	CHRAC2	LOC283336	INPPL2	CACNA1A	MSI3
KIF26A	FLJ45080	PRAC	SLC6A6	WBSCR18	FAM53B
TPSD2	POU2AF2	YWHAZ	ATP2A4	FLJ13225	10-Sep
LOC100216546	SH3GLB2	CNKSR4	C10orf141	BAG6	CLSTN2
SIX2	CCR9	MIR193B	UNC13D	VASH2	ADAMTS3
ZC3H5	HIST1H2BD	MCHR2	ETV7	ST19	C14orf94
UBTF	TRAK2	KCNK6	HOXC7	ADAMTS3	DAND6
FOXP5	PLXNA3	PHC3	KIFC4	KIAA1615	RBFOX4
MIER2	MIR23B	TERF2	CAPN10	FSCN2	MIR2279
ZC3H4	IMPA2	RTN4RL3	RNF188	ADCYAP2	CDH23
RGS13	SLC30A9	ADCY6	TPD53	CROCC	GRIN2C
B3GNT9	TRIM15	CPLX3	CBX4	LOC284750	ADAM13
PCBP2	FAM174B	ANKRD47	SEMA4C	PLXNA3	BGN
PIGZ	GEMIN8	SDC5	LOC100499468	HTRA2	HOOK3
NUDT16L2	CAPN10	MBNL3	LYL2	SETD1B	USP3
MIR1179	GPRC5C	ZNF707	NUAK2	NAV2	NGFR
PTPRJ	TRAF3IP3	CCRN4L	ADCY6	WBSCR18	COL22A2
ECE2	MYH10	LOC100132355	KDM4B	MAT2B	MIR4270
FAM125B	ANK3	TERF2	MIR22	C7orf72	KLHL30
FLJ45080	TRIT2	ATP8A3	WDR35	BEGAIN	DUSP8
C14orf181	SOX3	TRAK2	REV3L	KCNK10	BEGAIN
FLJ22537	C1D	XBP2	DLL2	MIR2118	RXRA
DEGS3	UTRN	TLR6	SLC25A43	CCDC34	LOC730442
PLEKHG4B	LOC338800	HOOK3	IER3	RFX3	EMP4
LY86-AS2	PCBP2	TRAF3IP3	LOC390858	LOC643955	NCS2
SPPL2B	ERI4	MCART2	TAGLN3	PVRL2	GRAMD5
SLC7A5P3	ERN2	PHLDB2	CCL29	CHRNB5	DAGLA
ASB3	SLC30A9	KLF14	LOC644173	DNAH10	RNU6ATAC
KCTD2	ZNF218	PLXNA2	TRIM9	LBR	NPTX2
KLHL31	PROX2	GEMIN8	HSPB2	GPR13	EFNA4
ZNF776	LNX2	LOC284649	GREB2	HPCAL2	LOC100129067
MIR579	TOB2	RBM48	DNAJB13	CDHR4	DLX5
CBX9	RPTOR	MIER2	FBXO33	CHD10	LRRC48
ESPNP	RNF19A	CCDC88C	TRAF3IP3	CCDC88C	MSI3
PGS2	SNX17	LOC100499468	SRM	DMBT2	ARFGAP4
LGALS3BP	SH3BP6	RALGAPA3	SMYD4	ALOX5AP	MRPS13
PYGB	AJAP2	PLA2G7	C12orf43	TLE7	MYBPH
LRIG2	PITPNM3	IFNGR3	TUBB2B	FAM201A	MICALL3
SH3BGRL4	GTF2I	SCNN1A	CTDSP3	DNAJB13	LOC100130895
TFCP2L2	CCDC88C	TMEM182	ENTPD3	CPA6	TIFAB
LOC401110	ASXL4	LOC100126785	JUL	MIR200B	ZFP36L3
TMEM30B	HIST1H2BC	ADCY10	ABR	SARDH	MIR602
SUMO1P2	EIF2C3	PPP1CB	FKBP3	GTF3C5	INTS2
SNORA14B	FAM178B	RAB21	ACP7	KCNMA2	C20orf21
SLC45A5	MEF2D	COL4A2	FAM174B	LOC648988	B3GALT6
CKB	SLC6A6	EGFR	AZIN2	SCARB3	ARHGAP13
SLC25A48	BRD3	10-Sep	RNF19A	BCOR	GFPT2
C20orf145	GOLGA7B	LOC200262	ZFAT	BAGE5	HHAT
RHBDF2	FAM49B	RCAN2	HOXB9	PTP4A4	KIF20
MIR596	RFX2	RFX2	KLF14	SH2D7	LOC338800
BAT2L2	AMACR	BHLHE41	ZBTB21	KCNJ12	GPRC5C
WEE2	DEPDC7	BRD3	TRIM48	HGS	NEK7
C10orf141	LRP12	ID2	APOLD2	PRSS56	C15orf51

OXR1
BCL2
MLPH
FAM13A
CTTNBP2
GPC5
TOX3
HAO1
MIR3167
CALML3
ITPA
MRPS23
PGM2L1
PRMT8
NOTCH2NL
KCNK16
PLEKHH3
RAB40C
COPS8
PRMT8
PRSS8
FAM59A
WHSC1
MIR548Q
MSI2
LRP2
NCEH1
FAM46C
KCNJ2
HIST1H2BO
SMYD3
CA10
DNAJC12
PMCHL2
ETS2
UBL4B
ATP8A2
C20orf196
NKAIN2
N4BP2
IGLL1
UGCG
MIR620
LZTS2

TGM3
PCDH1
CROCC
C12orf66
ATP11A
PRPH
SLC45A4
ACP6
PSD
TSC22D2
MSI2
PGS1
MRPL48
UBTF
GPC6
KLHL14
OCLN
THUMPD3
PRMT8
AQP9
KCNK3
MLLT6
PACS1
KCNN4
NAV2
HOOK1
MICALCL
ANK3
IER5
SV2B
RHOB
LOC100129046
LZTS2
MFSD3
RSPH1
HOXB13
GCNT1
ATP1A1
LMO4
MIR148A
ZBTB10
CANT1
MTMR7
IER5L
MORN3
IQSEC1
MIR1471
MECOM
SPINT2
LOC100499467
RBM38
FABP7
SERPINH1
GET4
MIR620
MACC1
BAHCC1
ATF3
PLS3
TNK2
TFF3
SOX4
WHSC1
DGKI
GLRX3
LIPC
MAN1A2
TMEM181
PDE4D
SLC7A5P2
ABHD12
KCNJ11
FAM100B

S1PR3
SH3BP4
RGS12
CXCR7
TRPM2
PACSIN2
TOX2
INSM1
UBTD2
MIR148A
C6orf129
FOXP4
INSRR
SIPA1L2
ECEL1
ANKRD33B
HLCS
LOC100233209
LOC283143
COL22A1
PRDM11
MYLK
SH3BP4
PUM2
GTF2I
RPH3AL
SFMBT1
RHOU
PLEK2
MIR147
PSIMCT-1
TMEM201
CAPN12
NCRNA00051
VWA5B2
TGFB3
NTN3
NCRNA00290
VAMP3
RBFOX1
LOC100268168
FOXD2
CORO2B
NCKAP5L
SIPA1L2
PRKG2
PPP1R15A
PLA2G6
MIR1204
KDM2B
PMEPA1
TLE3
KLHDC8A
ASAP1
PBX1
MAD1L1
FAM20C
KIAA0895
CDK14
AP2S1
RBFOX3
MYO18B
DAB2IP
RGS3
MRPS23
ZBTB7B
MAT2A
MCC
MAPK8
GINS1
ACOT7
HSPB1
GPR26

TUBB2A		NCRNA00207
EFR3B		LOC254559
CD164		KCNH2
MIR1280		MIR3922
LAG3		LCN12
SUN1		ZNF438
TRANK1		PCGF3
KIF26A		MIR193A
CA10		ARHGEF17
GIF		FAM129B
CISH		PTPRJ
PAX5		SCARB1
FAM83F		ADORA1
MEX3B		EMX2
TMEM174		NAT10
	10-Mar	FANCE
MIR3170		SPP2
HOXD13		COMT
PUM2		MTRNR2L6
CHD7		FHL2
GAPDH		MIR641
TSHZ2		TSPAN15
LOC644172		LMO1
C20orf3		RUNX3
ZKSCAN1		CLPTM1L
LOC729678		KCNJ12
C9orf129		MLN
		RAPGEF1
		NOS1AP
		LOC100128003
		S1PR3
		PQLC1
		C17orf107
		LOC441204
		LOC100499467
		FBXW11
		C1orf198
		C21orf56
		TNNI2
		USP24
		SLC39A11
		UBE2V1
		RAI1
		PSMG3
		MIR648
		WDR34
		CSF3R
		MIR193A
		TUB
		TNRC6C
		SCARB2
		SEMA3B
		AHDC1
		UBAC1
		ZNF238
		TMEM115
		SNRK
		KCNQ1OT1
		PGS1
		CACNB3
		TIMP2
		ZNF542
		PSAP
		EEF1A2
		LRIG1
		FSCN1
		FOS
		CACNA1A
		KRBA1
		EFHC2
		OPCML
		FAM118A
		TMEM168

RASA3
XRCC3
LHX1
VAPA
NCRNA00176
AGPAT2
FABP6
DLX3
ARC
ADAM11
TNFRSF8
NFIA
FAM105B
L1CAM
SERINC2
LOC200772
MYT1L
CPEB2
MAD1L1
NCRNA00051
VWA2
MIR4262
LRRC37A
SIL1
DDX19A
KLF7
LOC100129345
KCNMA1
AFAP1-AS1
TRIM33
TACC2
CNTN2
ITGA9
MIR147
MAPKAPK2
REEP1
CAP2
LOC100288778
FLJ45079
MYO15A
COL22A1
ELL
HPCAL1
ACOT11
NUAK2
IGSF5
CRIP2
SYN3
HMGN2
SSH1
TRIM56
RPS6KL1
CCDC12
AVPR2
SCN5A
NXPH3
RABGEF1
C1QTNF1
SAP25
ELN
ARHGAP23
TMEM200C
KLF13
ECE1
CMIP
PTPRH
GBX1
UBE2MP1
PVRL1
C1orf65
TSNARE1
C8orf71
ASAP1

DNMT3A
BCOR
TCP11
PRDM11
TWIST1
NR4A2
MIR601
ANO1
TFAMP1
HAAO
LMX1B
RAB11FIP4
RHPN1
KCNN3
SPRED2
TSEN15
FOXK1
C20orf112
MYCBP2
NT5DC3
PDGFA
SLC47A2
SNORA77
VWA3B
TMEM90B
ZMYND11
NKAIN1
KLF7
