# Supplementary material for: Subtype heterogeneity and epigenetic convergence in neuroendocrine prostate cancer
Source: Nat Commun. 2021 Oct 1;12:5775. doi: 10.1038/s41467-021-26042-z (PMC8486778; doi:10.1038/s41467-021-26042-z)
Supplement: Supplementary file 5 — Reporting Summary [file 41467_2021_26042_MOESM5_ESM.pdf]

Corresponding author(s): Henry W. Long

Last updated by author(s): Aug 12, 2021

Reporting Summary

Nature Research wishes to improve the reproducibility of the work that we publish. This form provides structure for consistency and transparency in reporting. For further information on Nature Research policies, see our Editorial Policies and the Editorial Policy Checklist.

Statistics

For all statistical analyses, confirm that the following items are present in the figure legend, table legend, main text, or Methods section.

n/a	Confirmed	
☐	☒	The exact sample size (*n*) for each experimental group/condition, given as a discrete number and unit of measurement
☐	☒	A statement on whether measurements were taken from distinct samples or whether the same sample was measured repeatedly
☐	☒	The statistical test(s) used AND whether they are one- or two-sided<br>*Only common tests should be described solely by name; describe more complex techniques in the Methods section.*
☐	☒	A description of all covariates tested
☐	☒	A description of any assumptions or corrections, such as tests of normality and adjustment for multiple comparisons
☐	☒	A full description of the statistical parameters including central tendency (e.g. means) or other basic estimates (e.g. regression coefficient) AND variation (e.g. standard deviation) or associated estimates of uncertainty (e.g. confidence intervals)
☐	☒	For null hypothesis testing, the test statistic (e.g. *F*, *t*, *r*) with confidence intervals, effect sizes, degrees of freedom and *P* value noted<br>*Give P values as exact values whenever suitable.*
☒	☐	For Bayesian analysis, information on the choice of priors and Markov chain Monte Carlo settings
☒	☐	For hierarchical and complex designs, identification of the appropriate level for tests and full reporting of outcomes
☐	☒	Estimates of effect sizes (e.g. Cohen's *d*, Pearson's *r*), indicating how they were calculated

Our web collection on statistics for biologists contains articles on many of the points above.

Software and code

Policy information about availability of computer code

Data collection	No software was used for data collection.
Data analysis	Chilin Pipeline 2.0.0 (http://cistrome.org/chilin/)<br>BWA (version 0.7.17-r1188)<br>MACS2 (version v2.1.2)<br>ROSE (version 1.0, http://younglab.wi.mit.edu/super_enhancer_code.html)<br>Cistrome (http://cistrome.org/)<br>Integrative genomics viewer (IGV v2.3) (https://software.broadinstitute.org/software/igv/)<br>Deeptools (version 2.5.4)<br>Viper Pipeline (version f78c6b0) (https://bitbucket.org/cfce/viper/src/master/)<br>GREAT (version 4.0.4 http://great.stanford.edu/public/html/)<br>Cufflinks (version 2.2.1 http://cole-trapnell-lab.github.io/cufflinks/)<br>Cell Ranger (version 3.1.0)<br>Cell Ranger ATAC (version 1.1.0)<br>Seurat (version 3 https://satijalab.org/seurat/vignettes.html)<br>GenomicRanges (https://bioconductor.org/packages/release/bioc/html/GenomicRanges.html)<br>CGA WES Characterization pipeline (https://portal.firecloud.org/#methods/getzlab/CGA_WES_Characterization_Pipeline_v0.1_Dec2 018/)<br>ContEst version4 GATK (https://software.broadinstitute.org/cancer/cga/contest)<br>MuTect (version 1.1.6)<br>Strelka (version 1.0.11)<br>GSVA (version 1.40.1, https://www.bioconductor.org/packages/release/bioc/html/GSVA.html)<br>MuTect2.1 (https://gatk.broadinstitute.org/hc/en-us/articles/360037593851-Mutect2)

DeTiN (version 47efdff) (https://github.com/getzlab/deTiN)
VEP release 104 (https://www.ensembl.org/vep)
Oncotator version4 GATK (https://software.broadinstitute.org/cancer/cga/oncotator)
vcf2maf (version 1.6.17) (https://github.com/mskcc/vcf2maf)
ConcatRef v1.0 (https://zenodo.org/record/3465870#.XzCushNKi3I)
Absolute v1.0 (https://software.broadinstitute.org/cancer/cga/absolute)
COBRA v1.0 (https://bitbucket.org/cfce/cobra/src/master/)
scCNV v1.0 (https://qiuxintao101.github.io/scCNV/)

For manuscripts utilizing custom algorithms or software that are central to the research but not yet described in published literature, software must be made available to editors and reviewers. We strongly encourage code deposition in a community repository (e.g. GitHub). See the Nature Research guidelines for submitting code & software for further information.

Data

Policy information about availability of data

All manuscripts must include a data availability statement. This statement should provide the following information, where applicable:

- Accession codes, unique identifiers, or web links for publicly available datasets
- A list of figures that have associated raw data
- A description of any restrictions on data availability

Figures 1-4 and supplementary figures 1- 4 all have associated raw data. These ChIP-seq data, RNA-seq data and ATAC-seq data have been uploaded to GEO (GSE156292)
In Figure 1,2 we compare our data to previously published data available on the TCGA (https://portal.gdc.cancer.gov/) and GEO (GSE110853) and GSM3321000.

Field-specific reporting

Please select the one below that is the best fit for your research. If you are not sure, read the appropriate sections before making your selection.

- [x] Life sciences
- [ ] Behavioural & social sciences
- [ ] Ecological, evolutionary & environmental sciences

For a reference copy of the document with all sections, see nature.com/documents/nr-reporting-summary-flat.pdf

Life sciences study design

All studies must disclose on these points even when the disclosure is negative.

Sample size	We did not perform sample size calculation and used all available cases to maximize our power for comparison in our chromatin analyses.
Data exclusions	We excluded datasets that were less than 1000 peaks identified by MACS2.
Replication	A subset of the LuCaP samples were replicated twice to establish the consistency of our protocol.
Randomization	Not relevant as there were no clinical treatments being tested.
Blinding	Not relevant as there were no clinical treatments being tested.

Reporting for specific materials, systems and methods

We require information from authors about some types of materials, experimental systems and methods used in many studies. Here, indicate whether each material, system or method listed is relevant to your study. If you are not sure if a list item applies to your research, read the appropriate section before selecting a response.

Materials & experimental systems

n/a	Involved in the study	
[ ]	[x]	Antibodies
[ ]	[x]	Eukaryotic cell lines
[x]	[ ]	Palaeontology and archaeology
[x]	[ ]	Animals and other organisms
[ ]	[x]	Human research participants
[x]	[ ]	Clinical data
[x]	[ ]	Dual use research of concern

Methods

n/a	Involved in the study	
[ ]	[x]	ChIP-seq
[x]	[ ]	Flow cytometry
[x]	[ ]	MRI-based neuroimaging

Antibodies

Antibodies used	H3K27Ac antibody (Diagenode cat# C15410196), ASCL1 antibody (abcam ab74065), NEUROD1 antibody (Cell Signalling mAb #4373,

	clone D35G2)
Validation	H3K27Ac, ASCL1 and NEUROD1 are validated for ChIP applications by qPCR for enrichment at known binding sites in validated cell lines as described on the manufacturers website.

Eukaryotic cell lines

Policy information about cell lines

Cell line source(s)	H660 was obtained from ATCC.<br>EF-1 cells were obtained from Leigh Ellis (DFCI).
Authentication	Not authenticated.
Mycoplasma contamination	All cell lines tested negative for mycoplasma.
Commonly misidentified lines<br>(See ICLAC register)	no commonly misidentified cell lines were used in the study.

Human research participants

Policy information about studies involving human research participants

Population characteristics	Patients were all male with median age at diagnosis in years (SD) of 64 (8.3), median age at death in years (SD) was 70 (9.2), median PSA at death in ng/mL (range) was 288.1 (0.2-5690.8), Gleason score (range) was 6-10. All patients had received Androgen Ablation Therapy.
Recruitment	All patients signed informed consent for a rapid autopsy, under the aegis of the Prostate Cancer Donor Program at the University of Washington. Patients are all male with late stage disease and there are no known biases that may affect the results presented in this paper.
Ethics oversight	The Institutional Review Board of the University of Washington (IRB #2341) approved this study.

Note that full information on the approval of the study protocol must also be provided in the manuscript.

ChIP-seq

Data deposition

- [x] Confirm that both raw and final processed data have been deposited in a public database such as GEO.
- [x] Confirm that you have deposited or provided access to graph files (e.g. BED files) for the called peaks.

Data access links<br>*May remain private before publication.*	GEO access: data uploaded, GSE156292
Files in database submission	20190605_ASCL1_2Ab_145_1_PC7059_S2_R1_001.fastq.gz<br>20190605_ASCL1_2Ab_145_1_PC7059_S2_R2_001.fastq.gz<br>20190626_PC2_93_ASCL1_PC7165_S2_R1_001.fastq.gz<br>20190626_PC5_49_ASCL1_PC7165_S5_R1_001.fastq.gz<br>20190731_H660_ASCl1_KL7258_S7_R1_001.fastq.gz<br>20190828_Lucap173_1E_H3K27Ac_KL7362_KL7362_S7_R1_001.fastq.gz<br>20191030_MKCC_EF1-H3K27AC_KL7532_S9_R1_001.fastq.gz<br>20191030_MKCC_EF1-H3K27AC_KL7532_S9_R2_001.fastq.gz<br>20200116_LUCAP173_1_S1_NeuroD_SS7756_S1_R1_001.fastq.gz<br>K27PX1731_1.fq.gz<br>K27PX1731_2.fq.gz<br>ASCL1_2Ab_145_1.rep1_treat_pileup.bw<br>EF1_cfce.rep1_treat_pileup.bw<br>H660_ASCl1.rep1_treat_pileup.bw<br>K27ac_PDX_173_1_new.rep1_treat_pileup.bw<br>Lucap173_1E_H3K27Ac.rep1_treat_pileup.bw<br>K27ac_1452_LP_USPD16086066_HLLNWBBXX_L4_1.fq.gz<br>K27ac_1452_LP_USPD16086066_HLLNWBBXX_L4_2.fq.gz<br>S1451_LP_K27ac_USPD16084579_HJGLKBBXX_L7_1.fq.gz<br>S1451_LP_K27ac_USPD16084579_HJGLKBBXX_L7_2.fq.gz<br>S49_LP_K27ac_USPD16084583_HJGLKBBXX_L7_1.fq.gz<br>S49_LP_K27ac_USPD16084583_HJGLKBBXX_L7_2.fq.gz<br>s93_PDX_K27ac_USPD16086058_HLLNWBBXX_L3_1.fq.gz<br>s93_PDX_K27ac_USPD16086058_HLLNWBBXX_L3_2.fq.gz<br>K27ac_PDX_145_1.rep1_treat_pileup.bw<br>K27ac_PDX_145_2.rep1_treat_pileup.bw

	K27ac_PDX_49.rep1_treat_pileup.bw<br>K27ac_PDX_93.rep1_treat_pileup.bw<br>LUCAP173_1_S1_NeuroD.rep1_treat_pileup.bw<br>PC2_93_ASCL1.rep1_treat_pileup.bw<br>PC5_49_ASCL1.rep1_treat_pileup.bw
Genome browser session<br>(e.g. UCSC)	http://genome.ucsc.edu/cgi-bin/hgTracks?db=hg19&lastVirtModeType=default&lastVirtModeExtraState=&virtModeType=default&virtMode=0&nonVirtPosition=&position=chr2%3A121478088%2D121721286&hgsid=877034177_akxcOL5bINlaIoGMz2vnAjCkF2qs

Methodology

Replicates	Replicates were not used on PDX models after validation shown in Supplemental figure 1.
Sequencing depth	All libraries were sequenced to a minimum depth of 10M reads with exact numbers reported in Supplementary.
Antibodies	H3K27Ac antibody (Diagenode cat# C15410196), ASCL1 antibody (abcam ab74065), NEUROD1 antibody (Cell Signalling mAb #4373)
Peak calling parameters	Peaks were called with MACS2 using default parameters.<br>Super-enhancers were called using ROSE using default parameters.
Data quality	We required a FrIP > 2%; DNASE1 overlap > 85% and the number of peaks > 1,000 at an FDR of 0.001
Software	Chilin Pipeline 2.0.0 (http://cistrome.org/chilin/)<br>BWA (version 0.7.17-r1188)<br>MACS2 (version v2.1.2)<br>ROSE (http://younglab.wi.mit.edu/super_enhancer_code.html)<br>Cistrome (http://cistrome.org/)<br>Integrative genomics viewer (IGV v2.3) (https://software.broadinstitute.org/software/igv/)<br>Deeptools (version 2.5.4)<br>GREAT (http://great.stanford.edu/public/html/)<br>Cufflinks (http://cole-trapnell-lab.github.io/cufflinks/)<br>Cell Ranger (version 3.1.0)<br>Cell Ranger ATAC (version 1.2.0)<br>Seurat (https://satijalab.org/seurat/vignettes.html)<br>GenomicRanges (https://bioconductor.org/packages/release/bioc/html/GenomicRanges.html)<br>COBRA (https://bitbucket.org/cfce/cobra/src/master/)<br>GSVA (version 1.40.1)
